# Lengths for Which Fourth Degree PP Interleavers Lead to Weaker Performances Compared to Quadratic and Cubic PP Interleavers

**DOI:** 10.3390/e22010078

**Published:** 2020-01-08

**Authors:** Lucian Trifina, Daniela Tarniceriu, Jonghoon Ryu, Ana-Mirela Rotopanescu

**Affiliations:** 1 Department of Telecommunications and Information Technologies, “Gheorghe Asachi” Technical University, 700506 Iasi, Romania; tarniced@etti.tuiasi.ro (D.T.); mrotopanescu@etti.tuiasi.ro (A.-M.R.); 2 Samsung Electronics, Inc., Suwon 16677, Korea; jonghoon.ryu@samsung.com

**Keywords:** PP interleaver, 4-PP, minimum distance, upper bound, turbo codes

## Abstract

In this paper, we obtain upper bounds on the minimum distance for turbo codes using fourth degree permutation polynomial (4-PP) interleavers of a specific interleaver length and classical turbo codes of nominal 1/3 coding rate, with two recursive systematic convolutional component codes with generator matrix G=[1,15/13]. The interleaver lengths are of the form 16Ψ or 48Ψ, where Ψ is a product of different prime numbers greater than three. Some coefficient restrictions are applied when for a prime $p_{i}|\Psi$, condition $3∤(p_{i}-1)$ is fulfilled. Two upper bounds are obtained for different classes of 4-PP coefficients. For a 4-PP $f_{4}x^{4}+f_{3}x^{3}+f_{2}x^{2}+f_{1}x\;\left(\mathrm{mod}\;16k_{L}\Psi \right)$, $k_{L}\in \left\{1,3\right\}$, the upper bound of 28 is obtained when the coefficient $f_{3}$ of the equivalent 4-permutation polynomials (PPs) fulfills $f_{3}\in \left\{0,4\Psi \right\}$ or when $f_{3}\in \left\{2\Psi ,6\Psi \right\}$ and $f_{2}\in \left\{\left(4k_{L}-1\right)\cdot \Psi ,\left(8k_{L}-1\right)\cdot \Psi \right\}$, $k_{L}\in \left\{1,3\right\}$, for any values of the other coefficients. The upper bound of 36 is obtained when the coefficient $f_{3}$ of the equivalent 4-PPs fulfills $f_{3}\in \left\{2\Psi ,6\Psi \right\}$ and $f_{2}\in \left\{\left(2k_{L}-1\right)\cdot \Psi ,\left(6k_{L}-1\right)\cdot \Psi \right\}$, $k_{L}\in \left\{1,3\right\}$, for any values of the other coefficients. Thus, the task of finding out good 4-PP interleavers of the previous mentioned lengths is highly facilitated by this result because of the small range required for coefficients $f_{4},f_{3}$ and $f_{2}$. It was also proven, by means of nonlinearity degree, that for the considered inteleaver lengths, cubic PPs and quadratic PPs with optimum minimum distances lead to better error rate performances compared to 4-PPs with optimum minimum distances.

## 1. Introduction

Error correcting codes with very good performances are an essential component for modern digital communications systems [1,2]. There are three classes of capacity approaching codes—turbo codes [3], low density parity check codes [4], and polar codes [5]. As a class of capacity approaching error correcting codes, turbo codes have gained much interest since their invention. One of the important research directions is increasing their minimum distances by different approaches. For example, recent works that deal with this topic are [6,7,8,9]. In [6], some upper bounds on the minimum distance for 3-dimensional turbo codes (conventional turbo codes with an additional patch) with quadratic permutation polynomial (QPP) interleavers were derived. Some example of QPPs found by random search that lead to significantly improved minimum distances are given. In [7], 4-dimensional (4-D) turbo codes are proposed and upper bounds on bit error rate (BER) performances are derived. These upper bounds imply weight enumerating functions and are derived by a simplified, augmented state-diagram-based method. This method is used to select different parameters of 4-D turbo codes so that they lead to lower BER values or higher minimum distances. In [8], a moment based augmented state diagram method was proposed to derive tighter upper bounds on BER performance for 4-D turbo codes. It was used to design 4-D turbo codes in order to achieve improved BER performances. In [9], a modified interleaver for a new structure of 4-D turbo codes, based on superposition modulation and grouped power allocation, has been proposed to improve the minimum distance. An appropriate design of interleavers for turbo codes considers the approaches that can lead to higher minimum distances. In this respect, knowing the upper bounds on the minimum distances for different classes of interleavers is important from the perspective of the measurements of their performances or limitations.

Permutation polynomial (PP) interleavers for turbo codes were introduced by Sun and Takeshita in 2005 [10]. They are very attractive because of their fully algebraic description, low memory, and high performance if they are appropriately chosen. Other very high-performing interleavers that are not fully algebraic described, are dithered relative prime (DRP) interleavers [11] and almost regular permutation interleavers [12]. Many results have been obtained regarding QPP interleavers. They have been chosen as interleavers for turbo codes in the long term evolution (LTE) standard [13]. The most notable results regarding QPP interleavers are those from [14,15]. In the last years, analysis and design of PP interleavers of degree greater than two have gained interest. For example, good interleavers based on PPs of degree greater than two have been obtained in [16,17,18].

In [15], upper bounds of the minimum distance for turbo codes with QPP interleavers and different interleaver lengths were obtained. Some upper bounds for PP interleavers of any degree were obtained in [19]. Recently, some results regarding upper bounds of the minimum distance for turbo codes with cubic permutation polynomial (CPP) interleavers have been acquired [20,21]. In this paper, for the first time, upper bounds of the minimum distance for turbo codes with fourth degree permutation polynomial (4-PP) interleavers of a specific type of interleaver length and for classical turbo codes of nominal 1/3 coding rate, with two recursive systematic convolutional component codes with generator matrix G=[1,15/13], were obtained. Specifically, for interleaver lengths of the form 16Ψ or 48Ψ, with Ψ, a product of prime numbers greater than three, the minimum distance is upper bounded by the value of 36 or 28, depending on the 4-PP coefficients. Some coefficient restrictions are applied when for a prime $p_{i}|\Psi$, condition $3∤(p_{i}-1)$ is fulfilled. If Ψ is a product of prime numbers $p_{i}>7$ so that $3|(p_{i}-1)$, the result in the paper is fully general.

The paper is structured as follows. In Section 2, some preliminary results about 4-PPs are given. The main results are worked through in Section 3. Some remarks and examples are given in Section 4, and Section 5 concludes the paper.

## 2. Preliminaries

### 2.1. Notation

In the paper we use the following notation:

- (modL), with *L* a positive integer, denotes modulo *L* operation;
- a∣b, with *a* and *b* positive integers, denotes *a* dividing *b*;
- a∤b, with *a* and *b* positive integers, denotes that *a* does not divide; *b*
- gcd(a,b), with *a* and *b* positive integers, denotes the greatest common divisor of *a* and *b*;
- $\log_{10}\left(\cdot \right)$ denotes base 10 logarithm;
- $e^{x}$ is the natural exponential function of variable *x*.

### 2.2. Results Regarding 4-PPs

A 4-PP modulo *L* is a fourth degree polynomial

(1) $$\pi \left(x\right)=\left(f_{1}x+f_{2}x^{2}+f_{3}x^{3}+f_{4}x^{4}\right)\;\left(\mathrm{mod}\;L\right),$$

so that for x∈{0,1,…,L−1}, values π(x)(modL) perform a permutation of the set {0,1,⋯,L−1}.

A 4-PP is true if the permutation it performs cannot be performed by a permutation polynomial of degree smaller than four.

Two 4-PPs with different coefficients are different if they lead to different permutations.

Conditions on coefficients $f_{1}$, $f_{2}$, $f_{3}$, and $f_{4}$ so that the fourth degree polynomial in (1) is a 4-PP modulo *L* have been obtained in [22]. Because we are interested in interleaver lengths of the form $16\cdot \prod_{i=1}^{N_{p}}p_{i}$ or $48\cdot \prod_{i=1}^{N_{p}}p_{i}$, with $N_{p}$ a positive integer, in Table 1 we give the coefficient conditions only for the primes 2, 3, and $p_{i}$, $i=1,2,\ldots ,N_{p}$, when the interleaver length is of the form

(2) $$\begin{array}{c} L=2^{n_{L,2}}\cdot 3^{n_{L,3}}\cdot \prod_{i=1}^{N_{p}}p_{i},\;\mathrm{with}\;n_{L,2}>1,n_{L,3}\in \left\{0,1\right\},\\ p_{i}>3,i=1,2,\ldots ,N_{p},p_{1}<p_{2}<\cdots <p_{N_{p}}. \end{array}$$

A 4-PP modulo *L*

(3) $$\rho \left(x\right)=\left(\rho_{1}x+\rho_{2}x^{2}+\rho_{3}x^{3}+\rho_{4}x^{4}\right)\;\left(\mathrm{mod}\;L\right),$$

is an inverse of the 4-PP in (1) if

(4) π(ρ(x))=x(modL),∀x∈{0,1,⋯,L−1}.

## 3. Main Results

In this section, we consider the interleaver lengths of the form

(5) $$L=16\cdot \prod_{i=1}^{N_{p}}p_{i}=2^{4}\cdot \prod_{i=1}^{N_{p}}p_{i}\;\mathrm{or}\;L=48\cdot \prod_{i=1}^{N_{p}}p_{i}=2^{4}\cdot 3\cdot \prod_{i=1}^{N_{p}}p_{i},$$

with $p_{i}$ different prime numbers so that $p_{i}>3$, $\forall i=1,2,\ldots ,N_{p}$, and $p_{1}<p_{2}<\cdots <p_{N_{p}}$.

For $p_{i}$ a prime so that $3∤(p_{i}-1)$, $i\in \{1,2,\ldots ,N_{p}\}$, we will consider only the 4-PPs with coefficients fulfilling conditions

(6) $$f_{1}\neq 0,f_{2}=0,f_{3}=0,f_{4}=0\;\left(\mathrm{mod}\;p_{i}\right).$$

In the following, we denote

(7) $$\prod_{i=1}^{N_{p}}p_{i}=\Psi .$$

The reason for which we focus on the interleaver lengths of the form given in (5) is as follows. In [17], 4-PPs of several lengths that lead to the best minimum distance of 36 were reported. We wanted to see if this minimum distance is a general upper bound for a general form of interleaver lengths. From the lengths in [17] for which the best minimum distance of 4-PPs is 36, we restrict ourselves to those of the form given in (5) and also we restrict ourselves to the coefficients fulfilling conditions (6) when $3∤(p_{i}-1)$ because, in this case, the possible coefficients of a true 4-PP are limited to a few values (see Lemma 1). This simplifies finding the coefficients of the inverse 4-PPs, and thus, the proofs for upper bounds on minimum distance for 4-PPs of the focal interleaver lengths. We note that increasing the power of primes in the product Ψ leads to more values of the possible coefficients of 4-PPs, and thus, finding the inverse 4-PPs is more complicated.

### 3.1. Methodology

The research methodology is similar to that from [20,21] and it is described in this subsection. To find upper bounds on the minimum distance for turbo codes that have 4-PP interleavers of lengths of the form given in (5), the research methodology assumes the following steps:

(1) For the interleaver lengths of the form given in (5), we found all possible values for the coefficients of true different 4-PPs. Thus, every 4-PP will have the coefficients equivalent to these found values.
(2) We proved that for the interleaver lengths in question, every true 4-PP has an inversely true 4-PP, extending the result from [23].
(3) For some 4-PPs with particular minimum distances, we found the interleaver patterns that lead to these minimum distances. There are several methods to find minimum distance of turbo codes with particular interleavers. The method from [24] or its improved version from [25] allow the determination of the true minimum distance ($d_{\min}$), but their complexity increases rapidly when increasing $d_{\min}$. Methods based on impulses of high amplitude inserted in the all-zero codeword and then decoding this perturbed codeword to give a decoded codeword of low weigth, are faster for high values of $d_{\min}$ and useful for finding interleaver patterns. Double impulse method (DIM) and triple impulse method (TIM) [26] are more reliable among the impulse based methods. An alternative method of TIM is the full range double impulse method from [27] (denoted DIMK in [28]), wherein the reliability of DIM is improved by a full range for the second impulse, instead of a limited range search. The complexity of impulse based methods can be reduced for structured interleavers (such as 4-PP ones) [29]. We have made use of DIMK method for finding the interleaver patterns from Theorems 1 and 2.
(4) Finally, we proved that these critical interleaver patterns always appear for 4-PPs of the interleaver lengths in question and classes of their coefficients.

### 3.2. Coefficients of 4-PPs for the Interleaver Lengths of the Form 16Ψ or 48Ψ

In [23], we derived a pure mathematical result. For interleaver lengths of the form 16Ψ, in Lemma 3.1 from [23], the possible values of the coefficients of a true 4-PP were obtained. Lemma 3.2 provides an equation to determine the coefficients of an inverse true 4-PP without giving all its possible solutions. The next two lemmas are extensions of Lemmas 3.1 and 3.2 from [23]. Lemma 1 gives the coefficients of a true 4-PP and Lemma 2 gives the coefficients of an inverse true 4-PP of a true 4-PP, fulfilling conditions (6) when $3∤(p_{i}-1)$, the modulo of an integer of the form given in (5). These two lemmas are necessary to derive the upper bounds on the minimum distance from Section 3.3. We note that because of coefficient conditions 2) from Table 1, the extension of the results from [23] to the interleaver lengths of the form 48Ψ is not straightforward. Because 3∤Ψ, we can have any of the following combinations of $f_{4}$ and $f_{2}$ coefficients’ conditions: (1) $f_{4}=1\;\left(\mathrm{mod}\;3\right)$, $f_{2}=2\;\left(\mathrm{mod}\;3\right)$; (2) $f_{4}=2\;\left(\mathrm{mod}\;3\right)$, $f_{2}=1\;\left(\mathrm{mod}\;3\right)$, with any of the following combinations of $f_{3}$ and $f_{1}$ coefficients conditions: (1) $f_{3}=0\;\left(\mathrm{mod}\;3\right)$ and $f_{1}\neq 0\;\left(\mathrm{mod}\;3\right)$; (2) $f_{3}=1\;\left(\mathrm{mod}\;3\right)$, $f_{1}\neq 2\;\left(\mathrm{mod}\;3\right)$; (3) $f_{3}=2\;\left(\mathrm{mod}\;3\right)$, $f_{1}\neq 1\;\left(\mathrm{mod}\;3\right)$. Therefore, we will have more different cases to determine the coefficients of an inverse 4-PP, as Tables 5–8 show show.

**Lemma** **1.** *Let the interleaver length be of the form given in *(5)*. Then all true different 4-PPs fulfilling conditions *(6)* when $3∤(p_{i}-1)$, have possible values for coefficients $f_{4}$, $f_{3}$, and $f_{2}$ equivalent to those given in Table 2 from the second, third, and fourth columns, respectively. Coefficient $f_{1}$ has to always be odd.*

**Proof.**  For the interleaver length of the form L=16Ψ, a true 4-PP is equivalent to a 4-PP for which $f_{2}<L/2=8\Psi$, $f_{3}<L/2=8\Psi$, and $f_{4}<L/8=2\Psi$. For the interleaver length of the form L=48Ψ, a true 4-PP is equivalent to a 4-PP for which $f_{2}<L/2=24\Psi$, $f_{3}<L/6=8\Psi$, and $f_{4}<L/24=2\Psi$. Taking into account the coefficient conditions for a 4-PP given in Table 1 and that Ψ is odd, coefficients $f_{2}$, $f_{3}$, and $f_{4}$ from Table 2 follows.

We note that when L=16Ψ or L=48Ψ, (from condition 1 in Table 1) $f_{1}$ becomes odd. □

**Lemma** **2.** *Let the interleaver length be of the form $L=16\cdot k_{L}\cdot \Psi$, with $k_{L}\in \left\{1,3\right\}$ and Ψ given in *(7)*. Then, a true 4-PP $\pi \left(x\right)=f_{1}x+f_{2}x^{2}+f_{3}x^{3}+f_{4}x^{4}\;\left(\mathrm{mod}\;L\right)$, fulfilling conditions *(6)* when $3∤(p_{i}-1)$, has an inverse true 4-PP $\rho \left(x\right)=\rho_{1}x+\rho_{2}x^{2}+\rho_{3}x^{3}+\rho_{4}x^{4}\;\left(\mathrm{mod}\;L\right)$, with*

(8) $$\begin{array}{c} \rho_{4}=f_{4}, \end{array}$$

(9) $$\begin{array}{c} \rho_{3}=k_{3,\rho }\cdot 2\Psi , \end{array}$$

(10) $$\begin{array}{c} \rho_{2}=\left(2k_{2,\rho }\cdot k_{L}-1\right)\cdot \Psi . \end{array}$$

*$\rho_{1}$ is the unique modulo L solution of the congruence $f_{1}\rho_{1}=\Psi \cdot k+1\;\left(\mathrm{mod}\;L\right)$. k, $k_{3,\rho }$, and $k_{2,\rho }$ are given in Table 3, Table 4, Table 5, Table 6, Table 7 and Table 8, according to the values of $k_{3,f}=f_{3}/\left(2\Psi \right)$, $k_{2,f}=\left(f_{2}/\Psi +1\right)/\left(2k_{L}\right)$, and $f_{1}\;\left(\mathrm{mod}\;16k_{L}\right)$.*

**Proof.**  ρ(x) is an inverse 4-PP of π(x) if

(11) π(ρ(x))=x(modL),∀x∈{0,1,…,L−1}.

Taking into account Lemma 1, after some algebraic manipulations, Equation (11) is equivalent to

$$\left(f_{1}\rho_{1}-1\right)\cdot x+\left(f_{1}\rho_{2}+f_{2}\rho_{1}^{2}\right)\cdot x^{2}+\left(f_{1}\rho_{3}+2f_{2}\rho_{2}\rho_{1}+f_{3}\rho_{1}^{3}\right)\cdot x^{3}+$$

$$+\left(f_{4}\rho_{1}^{4}+3f_{3}\rho_{1}^{2}\rho_{2}+2f_{2}\rho_{3}\rho_{1}+f_{2}\rho_{2}^{2}+f_{1}\rho_{4}\right)\cdot x^{4}+$$

$$+\left(4f_{4}\rho_{1}^{3}\rho_{2}+3f_{3}\rho_{1}^{2}\rho_{3}+3f_{3}\rho_{1}\rho_{2}^{2}+2f_{2}\rho_{4}\rho_{1}+2f_{2}\rho_{3}\rho_{2}\right)\cdot x^{5}+$$

$$+\left(4f_{4}\rho_{1}^{3}\rho_{3}+6f_{4}\rho_{1}^{2}\rho_{2}^{2}+3f_{3}\rho_{4}\rho_{1}^{2}+6f_{3}\rho_{1}\rho_{2}\rho_{3}+f_{3}\rho_{2}^{3}+2f_{2}\rho_{4}\rho_{2}+f_{2}\rho_{3}^{2}\right)\cdot x^{6}+$$

$$+\left(4f_{4}\rho_{4}\rho_{1}^{3}+12f_{4}\rho_{1}^{2}\rho_{2}\rho_{3}+4f_{4}\rho_{1}\rho_{2}^{3}+6f_{3}\rho_{4}\rho_{1}\rho_{2}+3f_{3}\rho_{1}\rho_{3}^{2}+3f_{3}\rho_{2}^{2}\rho_{3}+2f_{2}\rho_{4}\rho_{3}\right)\cdot x^{7}+$$

$$+\left(12f_{4}\rho_{1}^{2}\rho_{2}\rho_{4}+6f_{4}\rho_{1}^{2}\rho_{3}^{2}+12f_{4}\rho_{1}\rho_{2}^{2}\rho_{3}+6f_{3}\rho_{1}\rho_{3}\rho_{4}+f_{4}\rho_{2}^{4}+3f_{3}\rho_{2}^{2}\rho_{4}+3f_{3}\rho_{2}\rho_{3}^{2}+f_{2}\rho_{4}^{2}\right)\cdot x^{8}+$$

$$+\left(12f_{4}\rho_{1}^{2}\rho_{3}\rho_{4}+12f_{4}\rho_{1}\rho_{2}^{2}\rho_{4}+12f_{4}\rho_{1}\rho_{2}\rho_{3}^{2}+3f_{3}\rho_{1}\rho_{4}^{2}+4f_{4}\rho_{2}^{3}\rho_{3}+6f_{3}\rho_{2}\rho_{3}\rho_{4}+f_{3}\rho_{3}^{3}\right)\cdot x^{9}+$$

$$+\left(6f_{4}\rho_{1}^{2}\rho_{4}^{2}+24f_{4}\rho_{1}\rho_{2}\rho_{3}\rho_{4}+4f_{4}\rho_{1}\rho_{3}^{3}+4f_{4}\rho_{2}^{3}\rho_{4}+6f_{4}\rho_{2}^{2}\rho_{3}^{2}+3f_{3}\rho_{2}\rho_{4}^{2}+3f_{3}\rho_{3}^{2}\rho_{4}\right)\cdot x^{10}+$$

$$+\left(12f_{4}\rho_{2}^{2}\rho_{3}\rho_{4}+4f_{4}\rho_{2}\rho_{3}^{3}+12f_{4}\rho_{1}\rho_{2}\rho_{4}^{2}+12f_{4}\rho_{1}\rho_{3}^{2}\rho_{4}+3f_{3}\rho_{3}\rho_{4}^{2}\right)\cdot x^{11}+$$

$$+\left(6f_{4}\rho_{2}^{2}\rho_{4}^{2}+12f_{4}\rho_{2}\rho_{3}^{2}\rho_{4}+f_{4}\rho_{3}^{4}+12f_{4}\rho_{1}\rho_{3}\rho_{4}^{2}+f_{3}\rho_{4}^{3}\right)\cdot x^{12}+$$

$$+\left(4f_{4}\rho_{3}^{3}\rho_{4}+12f_{4}\rho_{2}\rho_{3}\rho_{4}^{2}+4f_{4}\rho_{1}\rho_{4}^{3}\right)\cdot x^{13}+\left(6f_{4}\rho_{3}^{2}\rho_{4}^{2}+4f_{4}\rho_{2}\rho_{4}^{3}\right)\cdot x^{14}+$$

(12) $$\begin{array}{c} +\left(4f_{4}\rho_{3}\rho_{4}^{3}\right)\cdot x^{15}+\left(f_{4}\rho_{4}^{4}\right)\cdot x^{16}=0\;\left(\mathrm{mod}\;L\right),\forall x\in \left\{0,1,\ldots ,L-1\right\}. \end{array}$$

Because π(x) and ρ(x) are true 4-PPs, from Lemma 1 it results that $\rho_{4}=f_{4}=\Psi$, $\rho_{3}=k_{3,\rho }\cdot 2\Psi$, $f_{3}=k_{3,f}\cdot 2\Psi$, with $k_{3,\rho },k_{3,f}\in \left\{0,1,2,3\right\}$, $\rho_{2}=\left(2k_{2,\rho }\cdot k_{L}-1\right)\cdot \Psi$, and $f_{2}=\left(2k_{2,f}\cdot k_{L}-1\right)\cdot \Psi$, with $k_{2,\rho },k_{2,f}\in \left\{1,2,3,4\right\}$, $k_{L}\in \left\{1,3\right\}$. Because $p_{i}$ is odd $\forall i\in \{1,2,\ldots ,N_{p}\}$, Ψ from (7) is also odd. Then, we can have Ψ=1(mod8), Ψ=3(mod8), Ψ=5(mod8), or Ψ=7(mod8). Then, 2Ψ=2(mod8) or 2Ψ=6(mod8). Because every $p_{i}$ is odd and $3∤p_{i}$, we can have Ψ=1(mod24), Ψ=5(mod24), Ψ=7(mod24), Ψ=11(mod24), Ψ=13(mod24), Ψ=17(mod24), Ψ=19(mod24), or Ψ=23(mod24). Then, 2Ψ=2(mod24), 2Ψ=10(mod24), 2Ψ=14(mod24), or 2Ψ=22(mod24).

Thus, for $L=16k_{L}\Psi$, $k_{L}\in \left\{1,3\right\}$, $\rho_{3}=k_{3,\rho }\cdot 2\Psi$, $f_{3}=k_{3,f}\cdot 2\Psi$, with $k_{3,\rho },k_{3,f}\in \left\{0,1,2,3\right\}$, $\rho_{2}=\left(2k_{2,\rho }k_{L}-1\right)\cdot \Psi$, and $f_{2}=\left(2k_{2,f}k_{L}-1\right)\cdot \Psi$, with $k_{L}\in \left\{1,3\right\}$, $k_{2,\rho },k_{2,f}\in \left\{1,2,3,4\right\}$, (12) is equivalent to

$$\left(f_{1}\rho_{1}-1\right)\cdot x+\Psi \cdot \left(f_{1}\cdot \left(2k_{2,\rho }k_{L}-1\right)+\left(2k_{2,f}k_{L}-1\right)\cdot \rho_{1}^{2}\right)\cdot x^{2}+$$

$$+2\Psi \cdot \left(f_{1}k_{3,\rho }+\left(2k_{2,f}k_{L}-1\right)\cdot \left(2k_{2,\rho }k_{L}-1\right)\Psi \rho_{1}+k_{3,f}\rho_{1}^{3}\right)\cdot x^{3}+$$

$$+\Psi \cdot (\rho_{1}^{4}+6k_{3,f}\cdot \left(2k_{2,\rho }k_{L}-1\right)\cdot \Psi \cdot \rho_{1}^{2}+4k_{3,\rho }\cdot \left(2k_{2,f}k_{L}-1\right)\cdot \Psi \cdot \rho_{1}+$$

$$+\left(2k_{2,f}k_{L}-1\right)\cdot {\left(2k_{2,\rho }k_{L}-1\right)}^{2}\cdot \Psi^{2}+f_{1})\cdot x^{4}+$$

$$+2\Psi^{2}\cdot (2\cdot \left(2k_{2,\rho }k_{L}-1\right)\cdot \rho_{1}^{3}+6k_{3,f}k_{3,\rho }\rho_{1}^{2}+3k_{3,f}\cdot {\left(2k_{2,\rho }k_{L}-1\right)}^{2}\cdot \Psi \rho_{1}+\left(2k_{2,f}k_{L}-1\right)\cdot \rho_{1}+$$

$$+2\cdot \left(2k_{2,f}k_{L}-1\right)\cdot \left(2k_{2,\rho }k_{L}-1\right)\cdot k_{3,\rho }\Psi )\cdot x^{5}+$$

$$+2\Psi^{2}\cdot (4k_{3,\rho }\rho_{1}^{3}+3\cdot {\left(2k_{2,\rho }k_{L}-1\right)}^{2}\cdot \Psi \rho_{1}^{2}+3k_{3,f}\rho_{1}^{2}+12k_{3,f}k_{3,\rho }\cdot \left(2k_{2,\rho }k_{L}-1\right)\cdot \Psi \rho_{1}+$$

$$+k_{3,f}\cdot {\left(2k_{2,\rho }k_{L}-1\right)}^{3}\cdot \Psi^{2}+\left(2k_{2,f}k_{L}-1\right)\cdot \left(2k_{2,\rho }k_{L}-1\right)\cdot \Psi +2\cdot \left(2k_{2,f}k_{L}-1\right)\cdot k_{3,\rho }^{2}\Psi )\cdot x^{6}+$$

$$+4\Psi^{2}\cdot (\rho_{1}^{3}+6k_{3,\rho }\cdot \left(2k_{2,\rho }k_{L}-1\right)\cdot \Psi \rho_{1}^{2}+{\left(2k_{2,\rho }k_{L}-1\right)}^{3}\cdot \Psi^{2}\rho_{1}+3k_{3,f}\cdot \left(2k_{2,\rho }k_{L}-1\right)\cdot \Psi \rho_{1}+$$

$$+6k_{3,f}k_{3,\rho }^{2}\Psi \rho_{1}+3k_{3,f}k_{3,\rho }\cdot {\left(2k_{2,\rho }k_{L}-1\right)}^{2}\cdot \Psi^{2}+k_{3,\rho }\cdot \left(2k_{2,f}k_{L}-1\right)\cdot \Psi )\cdot x^{7}+$$

$$+\Psi^{3}\cdot (12\cdot \left(2k_{2,\rho }k_{L}-1\right)\cdot \rho_{1}^{2}+24k_{3,\rho }^{2}\rho_{1}^{2}+24k_{3,\rho }\cdot {\left(2k_{2,\rho }k_{L}-1\right)}^{2}\cdot \Psi \rho_{1}+24k_{3,f}k_{3,\rho }\rho_{1}+$$

$$+{\left(2k_{2,\rho }k_{L}-1\right)}^{4}\cdot \Psi^{2}+6k_{3,f}\cdot {\left(2k_{2,\rho }k_{L}-1\right)}^{2}\cdot \Psi +24k_{3,f}k_{3,\rho }^{2}\cdot \left(2k_{2,\rho }k_{L}-1\right)\cdot \Psi +\left(2k_{2,f}k_{L}-1\right))\cdot x^{8}+$$

$$+2\Psi^{3}\cdot (12k_{3,\rho }\rho_{1}^{2}+3k_{3,f}\rho_{1}+24k_{3,\rho }^{2}\cdot \left(2k_{2,\rho }k_{L}-1\right)\cdot \Psi \rho_{1}+6\cdot {\left(2k_{2,\rho }k_{L}-1\right)}^{2}\cdot \Psi \rho_{1}+$$

$$+4k_{3,\rho }\cdot {\left(2k_{2,\rho }k_{L}-1\right)}^{3}\cdot \Psi^{2}+12k_{3,f}k_{3,\rho }\cdot \left(2k_{2,\rho }k_{L}-1\right)\cdot \Psi +8k_{3,f}k_{3,\rho }^{3}\Psi )\cdot x^{9}+$$

$$+2\Psi^{3}\cdot (3\rho_{1}^{2}+16k_{3,\rho }^{3}\Psi \rho_{1}+24k_{3,\rho }\cdot \left(2k_{2,\rho }k_{L}-1\right)\cdot \Psi \rho_{1}+2\cdot {\left(2k_{2,\rho }k_{L}-1\right)}^{3}\cdot \Psi^{2}+$$

$$+12k_{3,\rho }^{2}\cdot {\left(2k_{2,\rho }k_{L}-1\right)}^{2}\cdot \Psi^{2}+3k_{3,f}\cdot \left(2k_{2,\rho }k_{L}-1\right)\cdot \Psi +12k_{3,f}k_{3,\rho }^{2}\Psi )\cdot x^{10}+$$

$$+4\Psi^{4}\cdot (12k_{3,\rho }^{2}\rho_{1}+3\cdot \left(2k_{2,\rho }k_{L}-1\right)\cdot \rho_{1}+8k_{3,\rho }^{3}\cdot \left(2k_{2,\rho }k_{L}-1\right)\cdot \Psi +$$

$$+6k_{3,\rho }\cdot {\left(2k_{2,\rho }k_{L}-1\right)}^{2}\cdot \Psi +3k_{3,f}k_{3,\rho })\cdot x^{11}+$$

$$+2\Psi^{4}\cdot \left(12k_{3,\rho }\rho_{1}+8k_{3,\rho }^{4}\Psi +3\cdot {\left(2k_{2,\rho }k_{L}-1\right)}^{2}\cdot \Psi +24k_{3,\rho }^{2}\cdot \left(2k_{2,\rho }k_{L}-1\right)\cdot \Psi +k_{3,f}\right)\cdot x^{12}+$$

$$+4\Psi^{4}\cdot \left(\rho_{1}+8k_{3,\rho }^{3}\Psi +6k_{3,\rho }\cdot \left(2k_{2,\rho }k_{L}-1\right)\cdot \Psi \right)\cdot x^{13}+4\Psi^{5}\cdot \left(6k_{3,\rho }^{2}+\left(2k_{2,\rho }k_{L}-1\right)\right)\cdot x^{14}+$$

(13) $$+\left(8k_{3,\rho }\Psi^{5}\right)\cdot x^{15}+\Psi^{5}\cdot x^{16}=0\;\left(\mathrm{mod}\;16k_{L}\Psi \right),\forall x\in \left\{0,1,\ldots ,16k_{L}\Psi -1\right\}.$$

Because Ψ∣L, from (13) we have

(14) $$\begin{array}{c} \left(f_{1}\rho_{1}-1\right)\cdot x=0\;\left(\mathrm{mod}\;\Psi \right),\forall x\in \left\{0,1,\ldots ,16k_{L}\Psi -1\right\}. \end{array}$$

Equation (14) is equivalent to

(15) $$\begin{array}{c} f_{1}\rho_{1}=1\;\left(\mathrm{mod}\;\Psi \right)\Leftrightarrow f_{1}\rho_{1}=\Psi \cdot k+1\;\left(\mathrm{mod}\;16k_{L}\Psi \right),\;\mathrm{with}\;k\in \left\{0,1,2,\ldots ,16k_{L}-1\right\}. \end{array}$$

We note that when $k_{L}=1$, we have $\gcd(f_{1},16\Psi )=1$. According to Theorem 57 from [30], in this case congruence (15) has only one solution in variable $\rho_{1}$. When $k_{L}=3$, we can have $\gcd(f_{1},48\Psi )=3$. Thus, congruence (15) has three solutions, but as we will see, only one solution from the three will be valid.

With (15) and denoting $\Psi \;\left(\mathrm{mod}\;16k_{L}\right)=k_{\Psi }$, (13) is fulfilled if and only if

$$k\cdot x+\left(f_{1}\cdot \left(2k_{2,\rho }k_{L}-1\right)+\left(2k_{2,f}k_{L}-1\right)\cdot \rho_{1}^{2}\right)\cdot x^{2}+$$

$$+2\cdot \left(f_{1}k_{3,\rho }+\left(2k_{2,f}k_{L}-1\right)\cdot \left(2k_{2,\rho }k_{L}-1\right)k_{\Psi }\rho_{1}+k_{3,f}\rho_{1}^{3}\right)\cdot x^{3}+$$

$$+(\rho_{1}^{4}+6k_{3,f}\cdot \left(2k_{2,\rho }k_{L}-1\right)\cdot k_{\Psi }\cdot \rho_{1}^{2}+4k_{3,\rho }\cdot \left(2k_{2,f}k_{L}-1\right)\cdot k_{\Psi }\cdot \rho_{1}+$$

$$+\left(2k_{2,f}k_{L}-1\right)\cdot {\left(2k_{2,\rho }k_{L}-1\right)}^{2}\cdot k_{\Psi }^{2}+f_{1})\cdot x^{4}+$$

$$+2k_{\Psi }\cdot (2\cdot \left(2k_{2,\rho }k_{L}-1\right)\cdot \rho_{1}^{3}+6k_{3,f}k_{3,\rho }\rho_{1}^{2}+3k_{3,f}\cdot {\left(2k_{2,\rho }k_{L}-1\right)}^{2}\cdot k_{\Psi }\rho_{1}+\left(2k_{2,f}k_{L}-1\right)\cdot \rho_{1}+$$

$$+2\cdot \left(2k_{2,f}k_{L}-1\right)\cdot \left(2k_{2,\rho }k_{L}-1\right)\cdot k_{3,\rho }k_{\Psi })\cdot x^{5}+$$

$$+2k_{\Psi }\cdot (4k_{3,\rho }\rho_{1}^{3}+3\cdot {\left(2k_{2,\rho }k_{L}-1\right)}^{2}\cdot k_{\Psi }\rho_{1}^{2}+3k_{3,f}\rho_{1}^{2}+12k_{3,f}k_{3,\rho }\cdot \left(2k_{2,\rho }k_{L}-1\right)\cdot k_{\Psi }\rho_{1}+$$

$$+k_{3,f}\cdot {\left(2k_{2,\rho }k_{L}-1\right)}^{3}\cdot k_{\Psi }^{2}+\left(2k_{2,f}k_{L}-1\right)\cdot \left(2k_{2,\rho }k_{L}-1\right)\cdot k_{\Psi }+2\cdot \left(2k_{2,f}k_{L}-1\right)\cdot k_{3,\rho }^{2}k_{\Psi })\cdot x^{6}+$$

$$+4k_{\Psi }\cdot (\rho_{1}^{3}+6k_{3,\rho }\cdot \left(2k_{2,\rho }k_{L}-1\right)\cdot k_{\Psi }\rho_{1}^{2}+{\left(2k_{2,\rho }k_{L}-1\right)}^{3}\cdot k_{\Psi }^{2}\rho_{1}+3k_{3,f}\cdot \left(2k_{2,\rho }k_{L}-1\right)\cdot k_{\Psi }\rho_{1}+$$

$$+6k_{3,f}k_{3,\rho }^{2}k_{\Psi }\rho_{1}+3k_{3,f}k_{3,\rho }\cdot {\left(2k_{2,\rho }k_{L}-1\right)}^{2}\cdot k_{\Psi }^{2}+k_{3,\rho }\cdot \left(2k_{2,f}k_{L}-1\right)\cdot k_{\Psi })\cdot x^{7}+$$

$$+k_{\Psi }^{2}\cdot (12\cdot \left(2k_{2,\rho }k_{L}-1\right)\cdot \rho_{1}^{2}+24k_{3,\rho }^{2}\rho_{1}^{2}+24k_{3,\rho }\cdot {\left(2k_{2,\rho }k_{L}-1\right)}^{2}\cdot k_{\Psi }\rho_{1}+24k_{3,f}k_{3,\rho }\rho_{1}+$$

$$+{\left(2k_{2,\rho }k_{L}-1\right)}^{4}\cdot k_{\Psi }^{2}+6k_{3,f}\cdot {\left(2k_{2,\rho }k_{L}-1\right)}^{2}\cdot k_{\Psi }+24k_{3,f}k_{3,\rho }^{2}\cdot \left(2k_{2,\rho }k_{L}-1\right)\cdot k_{\Psi }+\left(2k_{2,f}k_{L}-1\right))\cdot x^{8}+$$

$$+2k_{\Psi }^{2}\cdot (12k_{3,\rho }\rho_{1}^{2}+3k_{3,f}\rho_{1}+24k_{3,\rho }^{2}\cdot \left(2k_{2,\rho }k_{L}-1\right)\cdot k_{\Psi }\rho_{1}+6\cdot {\left(2k_{2,\rho }k_{L}-1\right)}^{2}\cdot k_{\Psi }\rho_{1}+$$

$$+4k_{3,\rho }\cdot {\left(2k_{2,\rho }k_{L}-1\right)}^{3}\cdot k_{\Psi }^{2}+12k_{3,f}k_{3,\rho }\cdot \left(2k_{2,\rho }k_{L}-1\right)\cdot k_{\Psi }+8k_{3,f}k_{3,\rho }^{3}k_{\Psi })\cdot x^{9}+$$

$$+2k_{\Psi }^{2}\cdot (3\rho_{1}^{2}+16k_{3,\rho }^{3}k_{\Psi }\rho_{1}+24k_{3,\rho }\cdot \left(2k_{2,\rho }k_{L}-1\right)\cdot k_{\Psi }\rho_{1}+$$

$$+2\cdot {\left(2k_{2,\rho }k_{L}-1\right)}^{3}\cdot k_{\Psi }^{2}+12k_{3,\rho }^{2}\cdot {\left(2k_{2,\rho }k_{L}-1\right)}^{2}\cdot k_{\Psi }^{2}+3k_{3,f}\cdot \left(2k_{2,\rho }k_{L}-1\right)\cdot k_{\Psi }+12k_{3,f}k_{3,\rho }^{2}k_{\Psi })\cdot x^{10}+$$

$$+4k_{\Psi }^{3}\cdot (12k_{3,\rho }^{2}\rho_{1}+3\cdot \left(2k_{2,\rho }k_{L}-1\right)\cdot \rho_{1}+8k_{3,\rho }^{3}\cdot \left(2k_{2,\rho }k_{L}-1\right)\cdot k_{\Psi }+$$

$$+6k_{3,\rho }\cdot {\left(2k_{2,\rho }k_{L}-1\right)}^{2}\cdot k_{\Psi }+3k_{3,f}k_{3,\rho })\cdot x^{11}+$$

$$+2k_{\Psi }^{3}\cdot \left(12k_{3,\rho }\rho_{1}+8k_{3,\rho }^{4}k_{\Psi }+3\cdot {\left(2k_{2,\rho }k_{L}-1\right)}^{2}\cdot k_{\Psi }+24k_{3,\rho }^{2}\cdot \left(2k_{2,\rho }k_{L}-1\right)\cdot k_{\Psi }+k_{3,f}\right)\cdot x^{12}+$$

$$+4k_{\Psi }^{3}\cdot \left(\rho_{1}+8k_{3,\rho }^{3}k_{\Psi }+6k_{3,\rho }\cdot \left(2k_{2,\rho }k_{L}-1\right)\cdot k_{\Psi }\right)\cdot x^{13}+4k_{\Psi }^{4}\cdot \left(6k_{3,\rho }^{2}+\left(2k_{2,\rho }k_{L}-1\right)\right)\cdot x^{14}+$$

(16) $$\begin{array}{c} +\left(8k_{3,\rho }k_{\Psi }^{4}\right)\cdot x^{15}+k_{\Psi }^{4}\cdot x^{16}=0\;\left(\mathrm{mod}\;16k_{L}\right),\forall x\in \left\{0,1,\ldots ,16k_{L}-1\right\}. \end{array}$$

We note that the values of $k_{L}$ and $k_{\Psi }$ are given by the interleaver length.

Because Ψ is odd, when $k_{L}=1$ we can have $k_{\Psi }\in \left\{1,3,5,7,9,11,13,15\right\}$.

For $k_{L}=1$ we denote $k_{2,f}^{\prime }=2k_{2,f}-1$ and $k_{2,\rho }^{\prime }=2k_{2,\rho }-1$.

In this case, the variables from Equation (16) are $f_{1}\;\left(\mathrm{mod}\;16\right),\rho_{1}\;\left(\mathrm{mod}\;16\right)\in \left\{1,3,5,\ldots ,15\right\}$, $k_{3,\rho },k_{3,f}\in \left\{0,1,2,3\right\}$, $k_{2,\rho }^{\prime },k_{2,f}^{\prime }\in \left\{1,3,5,7\right\}$, *k*, and $k_{\Psi }$. The values of $k_{3,f}$, $k_{2,f}^{\prime }$, and $f_{1}\;\left(\mathrm{mod}\;16\right)$ are given by the true 4-PP, for which we want to find the inverse 4-PP; and $k_{\Psi }$ is given by the interleaver length. Given the values of $k_{\Psi }$, $k_{3,f}$, $k_{2,f}^{\prime }$, and $f_{1}\;\left(\mathrm{mod}\;16\right)$, we can find the coefficients of the inverse 4-PP by exhaustive searching for the rest of variables, $k_{3,\rho }$, $k_{2,\rho }^{\prime }$, $\rho_{1}\;\left(\mathrm{mod}\;16\right)$, and *k*. For each $k_{3,\rho }\in \left\{0,1,2,3\right\}$, $k_{2,\rho }^{\prime }\in \left\{1,3,5,7\right\}$, $\rho_{1}\;\left(\mathrm{mod}\;16\right)\in \left\{1,3,5,\ldots ,15\right\}$, and k∈{0,1,…,15}, we test if the left hand side term from (16), evaluated modulo 16, is equal to 0 for each x∈{0,1,…,15}. For a combination of variables $k_{\Psi }$, $k_{3,f}$, $k_{2,f}^{\prime }$, and $f_{1}\;\left(\mathrm{mod}\;16\right)$, only a combination of $k_{3,\rho }$, $k_{2,\rho }^{\prime }$, $\rho_{1}\;\left(\mathrm{mod}\;16\right)$, and *k* results in a solution of (16). In this way, using Matlab environment we found all the solutions of Equation (16). Solutions in variables *k*, $f_{1}\;\left(\mathrm{mod}\;16\right),\rho_{1}\;\left(\mathrm{mod}\;16\right)$, $k_{3,\rho },k_{3,f}$, and $k_{2,\rho }^{\prime },k_{2,f}^{\prime }$ are the same $\forall k_{\Psi }\in \left\{1,5,9,13\right\}$, and also solutions in the previously mentioned variables are the same $\forall k_{\Psi }\in \left\{3,7,11,15\right\}$. For every $k_{\Psi }\in \left\{1,3,5,\ldots ,15\right\}$, solutions of Equation (16) in variables *k*, $k_{3,\rho },k_{3,f}$, and $k_{2,\rho }^{\prime },k_{2,f}^{\prime }$ are the same $\forall f_{1}\;\left(\mathrm{mod}\;16\right)\in \left\{1,9\right\}$, or $\forall f_{1}\;\left(\mathrm{mod}\;16\right)\in \left\{3,11\right\}$, or $\forall f_{1}\;\left(\mathrm{mod}\;16\right)\in \left\{5,13\right\}$, or $\forall f_{1}\;\left(\mathrm{mod}\;16\right)\in \left\{7,15\right\}$. If the solution of Equation (16) in variable $\rho_{1}\;\left(\mathrm{mod}\;16\right)$ for $f_{1}\;\left(\mathrm{mod}\;16\right)=f_{1}\;\left(\mathrm{mod}\;8\right)=f_{1,8}\in \left\{1,3,5,7\right\}$ and the other variables with fixed values, is $\rho_{1,f_{1,8}}\;\left(\mathrm{mod}\;16\right)$, then solution of the same equation in variable $\rho_{1}\;\left(\mathrm{mod}\;16\right)$, for $f_{1}\;\left(\mathrm{mod}\;16\right)=f_{1,8}+8$, is $\left(\rho_{1,f_{1,8}}+8\right)\;\left(\mathrm{mod}\;16\right)$. Thus, for $k_{L}=1$, we can summarize the solutions of (16) for every $k_{\Psi }\;\left(\mathrm{mod}\;4\right)=k_{\Psi ,4}\in \left\{1,3\right\}$ and for every $f_{1}\;\left(\mathrm{mod}\;8\right)\in \left\{1,3,5,7\right\}$. These solutions are given in Table 3 and Table 4.

When $k_{L}=3$, because 3∤Ψ, we can have $k_{\Psi }\in \{1,5,7,11,13,17,19,23,25,29,31,35,$37,41,43,47}. We note that in this case, because of condition $\left(f_{1}+f_{3}\right)\neq 0\;\left(\mathrm{mod}\;3\right)$, we can have only some values for $f_{1}\;\left(\mathrm{mod}\;48\right)$, not every odd number. Because 3∤Ψ, we can have Ψ(mod3)∈{1,2}. Because $f_{3}=k_{3,f}\cdot 2\Psi$, with $k_{3,f}\in \left\{0,1,2,3\right\}$, we can have:

(1) $f_{1}\;\left(\mathrm{mod}\;48\right)\in \left\{1,5,7,11,13,17,19,23,25,29,31,35,37,41,43,47\right\}$, when Ψ(mod3)∈{1,2} and $k_{3,f}\in \left\{0,3\right\}$;
(2) $f_{1}\;\left(\mathrm{mod}\;48\right)\in \left\{1,3,7,9,13,15,19,21,25,27,31,33,37,39,43,45\right\}$, when Ψ(mod3)=1 and $k_{3,f}=2$ or when Ψ(mod3)=2 and $k_{3,f}=1$;
(3) $f_{1}\;\left(\mathrm{mod}\;48\right)\in \left\{3,5,9,11,15,17,21,23,27,29,33,35,39,41,45\right\}$, when Ψ(mod3)=1 and $k_{3,f}=1$ or when Ψ(mod3)=2 and $k_{3,f}=2$.

Solutions of Equation (16) for $k_{L}=3$ were found in the same way as for $k_{L}=1$, as it was previously explained. Solutions in variables *k*, $f_{1}\;\left(\mathrm{mod}\;48\right),\rho_{1}\;\left(\mathrm{mod}\;48\right)$, $k_{3,\rho },k_{3,f}$, and $k_{2,\rho },k_{2,f}$ are the same $\forall k_{\Psi }\in \left\{1,13,25,37\right\}$, or $\forall k_{\Psi }\in \left\{5,17,29,41\right\}$, or $\forall k_{\Psi }\in \left\{7,19,31,43\right\}$, or $\forall k_{\Psi }\in \left\{11,23,35,47\right\}$. For every $k_{\Psi }\in \left\{1,5,7,11,\ldots ,47\right\}$, solutions of Equation (16) in variables *k*, $k_{3,\rho },k_{3,f}$, and $k_{2,\rho },k_{2,f}$ are the same $f_{1}\;\left(\mathrm{mod}\;48\right)$ and for $\left(f_{1}+24\right)\;\left(\mathrm{mod}\;48\right)$. If the solution of Equation (16) in variable $\rho_{1}\;\left(\mathrm{mod}\;48\right)$ for $f_{1}\;\left(\mathrm{mod}\;48\right)=f_{1}\;\left(\mathrm{mod}\;24\right)=f_{1,24}$ and with the other variables with fixed values, is $\rho_{1,f_{1,24}}\;\left(\mathrm{mod}\;48\right)$, then solution of the same equation in variable $\rho_{1}\;\left(\mathrm{mod}\;48\right)$, for $f_{1}\;\left(\mathrm{mod}\;48\right)=f_{1,24}+24$, is $\left(\rho_{1,f_{1,24}}+24\right)\;\left(\mathrm{mod}\;48\right)$. Thus, for $k_{L}=3$, we can summarize the solutions of (16) for every $k_{\Psi }\;\left(\mathrm{mod}\;12\right)=k_{\Psi ,12}\in \left\{1,5,7,11\right\}$ and for every $f_{1}\;\left(\mathrm{mod}\;24\right)$. These solutions, found by means of Matlab software, are given in Table 5, Table 6, Table 7 and Table 8. □

We note that the inverse 4-PP from Lemma 2 is a true 4-PP, and thus the 4-PP π(x) does not admit an inverse QPP or CPP.

### 3.3. Upper Bounds on the Minimum Distances for 4-PP-Based Turbo Codes for Interleaver Lengths of the Form 16Ψ or 48Ψ

In this subsection, we prove that for the interleaver lengths of the form given in Equation (5), a true 4-PP leads to a minimum distance which is upper bounded by the value of 36 or 28, depending on the classes of coefficients, for a classical 1/3 rate turbo code with two recursive systematic convolutional (RSC) component codes having generator matrix G=[1,15/13] in octal form.

**Theorem** **1.** *Let the interleaver length be of the form given in *(5)*. Then, the minimum distance of the classical nominal 1/3 rate turbo code with two RSC codes parallel concatenated having the generator matrix G=[1,15/13] (in octal form) and 4-PP interleavers—fulfilling conditions (6) when $3∤(p_{i}-1)$, with coefficients $f_{4}=\Psi$, $f_{3}=k_{3,f}\cdot 2\Psi$, $k_{3,f}\in \left\{1,3\right\}$, $f_{2}=\left(2k_{2,f}\cdot k_{L}-1\right)\cdot \Psi$, $k_{2,f}\in \left\{1,2,3,4\right\}$, and $k_{L}\in \left\{1,3\right\}$—is upper bounded by the value of 36.*

**Figure 1:**
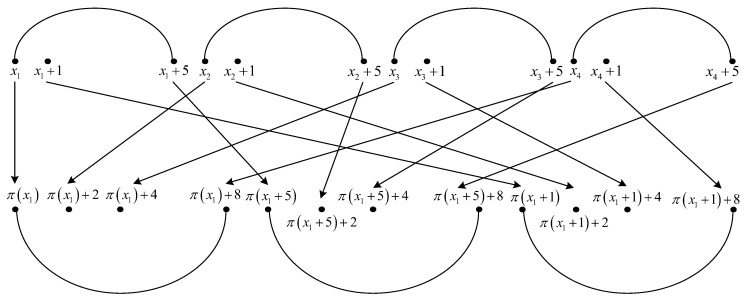
Critical interleaver pattern of size twelve for 4-PP-based interleavers.

**Proof.**  We consider the interleaver pattern of size twelve shown in Figure 1.

The twelve elements of permutation π(·) indicated in Figure 1 are written in detail below.

(17) $$\begin{array}{c} \left\{\begin{array}{c} x_{1}\to \pi \left(x_{1}\right)\\ x_{1}+1\to \pi \left(x_{1}+1\right)\;\left(\mathrm{mod}\;L\right)\\ x_{1}+5\to \pi \left(x_{1}+5\right)\;\left(\mathrm{mod}\;L\right)\\ x_{2}\to \pi \left(x_{2}\right)=\pi \left(x_{1}\right)+2\;\left(\mathrm{mod}\;L\right)\\ x_{2}+1\to \pi \left(x_{2}+1\right)=\pi \left(x_{1}+1\right)+2\;\left(\mathrm{mod}\;L\right)\\ x_{2}+5\to \pi \left(x_{2}+5\right)=\pi \left(x_{1}+5\right)+2\;\left(\mathrm{mod}\;L\right)\\ x_{3}\to \pi \left(x_{3}\right)=\pi \left(x_{1}\right)+4\;\left(\mathrm{mod}\;L\right)\\ x_{3}+1\to \pi \left(x_{3}+1\right)=\pi \left(x_{1}+1\right)+4\;\left(\mathrm{mod}\;L\right)\\ x_{3}+5\to \pi \left(x_{3}+5\right)=\pi \left(x_{1}+5\right)+4\;\left(\mathrm{mod}\;L\right)\\ x_{4}\to \pi \left(x_{4}\right)=\pi \left(x_{1}\right)+8\;\left(\mathrm{mod}\;L\right)\\ x_{4}+1\to \pi \left(x_{4}+1\right)=\pi \left(x_{1}+1\right)+8\;\left(\mathrm{mod}\;L\right)\\ x_{4}+5\to \pi \left(x_{4}+5\right)=\pi \left(x_{1}+5\right)+8\;\left(\mathrm{mod}\;L\right) \end{array}\right. \end{array}$$

Writing $x_{2}=\rho \left(\pi \left(x_{2}\right)\right)=\rho \left(\pi \left(x_{1}\right)+2\right)$ in the fifth and sixth equations from (17), $x_{3}=\rho \left(\pi \left(x_{3}\right)\right)=\rho \left(\pi \left(x_{1}\right)+4\right)$ in the eighth and ninth equations from (17), and $x_{4}=\rho \left(\pi \left(x_{4}\right)\right)=\rho \left(\pi \left(x_{1}\right)+8\right)$ in the eleventh and twelfth equation from (17), with $x_{1}=x$, we have

(18) $$\begin{array}{c} \left\{\begin{array}{c} \pi (\rho (\pi (x)+2)+1)=\pi (x+1)+2\;(\mathrm{mod}\;L)\\ \pi (\rho (\pi (x)+2)+5)=\pi (x+5)+2\;(\mathrm{mod}\;L)\\ \pi (\rho (\pi (x)+4)+1)=\pi (x+1)+4\;(\mathrm{mod}\;L)\\ \pi (\rho (\pi (x)+4)+5)=\pi (x+5)+4\;(\mathrm{mod}\;L)\\ \pi (\rho (\pi (x)+8)+1)=\pi (x+1)+8\;(\mathrm{mod}\;L)\\ \pi (\rho (\pi (x)+8)+5)=\pi (x+5)+8\;(\mathrm{mod}\;L) \end{array}\right. \end{array}$$

Taking into account that

(19) $$\begin{array}{c} \pi \left(a+b\right)=\pi \left(a\right)+\pi \left(b\right)+a\cdot b\cdot \left(2f_{4}\cdot \left(2a^{2}+3ab+2b^{2}\right)+3f_{3}\cdot \left(a+b\right)+2f_{2}\right), \end{array}$$

equations from (18) are equivalent to

(20) $$\left\{\begin{array}{c} \rho \left(\pi \left(x\right)+2\right)\cdot (2f_{4}\cdot \left(2\rho^{2}\left(\pi \left(x\right)+2\right)+3\rho \left(\pi \left(x\right)+2\right)+2\right)+\\ +3f_{3}\cdot \left(\rho \left(\pi \left(x\right)+2\right)+1\right)+2f_{2})=x\cdot \left(2f_{4}\cdot \left(2x^{2}+3x+2\right)+3f_{3}\cdot \left(x+1\right)+2f_{2}\right)\;\left(\mathrm{mod}\;L\right)\\ 5\cdot \rho \left(\pi \left(x\right)+2\right)\cdot (2f_{4}\cdot \left(2\rho^{2}\left(\pi \left(x\right)+2\right)+3\cdot 5\cdot \rho \left(\pi \left(x\right)+2\right)+2\cdot 5^{2}\right)+\\ +3f_{3}\cdot \left(\rho \left(\pi \left(x\right)+2\right)+5\right)+2f_{2})=\\ =5\cdot x\cdot \left(2f_{4}\cdot \left(2x^{2}+3\cdot 5\cdot x+2\cdot 5^{2}\right)+3f_{3}\cdot \left(x+5\right)+2f_{2}\right)\;\left(\mathrm{mod}\;L\right)\\ \rho \left(\pi \left(x\right)+4\right)\cdot (2f_{4}\cdot \left(2\rho^{2}\left(\pi \left(x\right)+4\right)+3\rho \left(\pi \left(x\right)+4\right)+2\right)+\\ +3f_{3}\cdot \left(\rho \left(\pi \left(x\right)+4\right)+1\right)+2f_{2})=x\cdot \left(2f_{4}\cdot \left(2x^{2}+3x+2\right)+3f_{3}\cdot \left(x+1\right)+2f_{2}\right)\;\left(\mathrm{mod}\;L\right)\\ 5\cdot \rho \left(\pi \left(x\right)+4\right)\cdot (2f_{4}\cdot \left(2\rho^{2}\left(\pi \left(x\right)+4\right)+3\cdot 5\cdot \rho \left(\pi \left(x\right)+4\right)+2\cdot 5^{2}\right)+\\ +3f_{3}\cdot \left(\rho \left(\pi \left(x\right)+4\right)+5\right)+2f_{2})=\\ =5\cdot x\cdot \left(2f_{4}\cdot \left(2x^{2}+3\cdot 5\cdot x+2\cdot 5^{2}\right)+3f_{3}\cdot \left(x+5\right)+2f_{2}\right)\;\left(\mathrm{mod}\;L\right)\\ \rho \left(\pi \left(x\right)+8\right)\cdot (2f_{4}\cdot \left(2\rho^{2}\left(\pi \left(x\right)+8\right)+3\rho \left(\pi \left(x\right)+8\right)+2\right)+\\ +3f_{3}\cdot \left(\rho \left(\pi \left(x\right)+8\right)+1\right)+2f_{2})=x\cdot \left(2f_{4}\cdot \left(2x^{2}+3x+2\right)+3f_{3}\cdot \left(x+1\right)+2f_{2}\right)\;\left(\mathrm{mod}\;L\right)\\ 5\cdot \rho \left(\pi \left(x\right)+8\right)\cdot (2f_{4}\cdot \left(2\rho^{2}\left(\pi \left(x\right)+8\right)+3\cdot 5\cdot \rho \left(\pi \left(x\right)+8\right)+2\cdot 5^{2}\right)+\\ +3f_{3}\cdot \left(\rho \left(\pi \left(x\right)+8\right)+5\right)+2f_{2})=\\ =5\cdot x\cdot \left(2f_{4}\cdot \left(2x^{2}+3\cdot 5\cdot x+2\cdot 5^{2}\right)+3f_{3}\cdot \left(x+5\right)+2f_{2}\right)\;\left(\mathrm{mod}\;L\right) \end{array}\right.$$

or

(21) $$\begin{array}{c} \left\{\begin{array}{c} 4f_{4}\cdot \rho^{3}\left(\pi \left(x\right)+2\right)+\left(6f_{4}+3f_{3}\right)\cdot \rho^{2}\left(\pi \left(x\right)+2\right)+\left(4f_{4}+3f3+2f_{2}\right)\cdot \rho \left(\pi \left(x\right)+2\right)=\\ =2f_{4}\cdot \left(2x^{3}+3x^{2}+2x\right)+3f_{3}\cdot \left(x^{2}+x\right)+2f_{2}\cdot x\;\left(\mathrm{mod}\;L\right)\\ 20f_{4}\cdot \rho^{3}\left(\pi \left(x\right)+2\right)+\left(150f_{4}+15f_{3}\right)\cdot \rho^{2}\left(\pi \left(x\right)+2\right)+\left(500f_{4}+75f3+10f_{2}\right)\cdot \rho \left(\pi \left(x\right)+2\right)=\\ =10f_{4}\cdot \left(2x^{3}+15x^{2}+50x\right)+15f_{3}\cdot \left(x^{2}+5x\right)+10f_{2}\cdot x\;\left(\mathrm{mod}\;L\right)\\ 4f_{4}\cdot \rho^{3}\left(\pi \left(x\right)+4\right)+\left(6f_{4}+3f_{3}\right)\cdot \rho^{2}\left(\pi \left(x\right)+4\right)+\left(4f_{4}+3f3+2f_{2}\right)\cdot \rho \left(\pi \left(x\right)+4\right)=\\ =2f_{4}\cdot \left(2x^{3}+3x^{2}+2x\right)+3f_{3}\cdot \left(x^{2}+x\right)+2f_{2}\cdot x\;\left(\mathrm{mod}\;L\right)\\ 20f_{4}\cdot \rho^{3}\left(\pi \left(x\right)+4\right)+\left(150f_{4}+15f_{3}\right)\cdot \rho^{2}\left(\pi \left(x\right)+4\right)+\left(500f_{4}+75f3+10f_{2}\right)\cdot \rho \left(\pi \left(x\right)+4\right)=\\ =10f_{4}\cdot \left(2x^{3}+15x^{2}+50x\right)+15f_{3}\cdot \left(x^{2}+5x\right)+10f_{2}\cdot x\;\left(\mathrm{mod}\;L\right)\\ 4f_{4}\cdot \rho^{3}\left(\pi \left(x\right)+8\right)+\left(6f_{4}+3f_{3}\right)\cdot \rho^{2}\left(\pi \left(x\right)+8\right)+\left(4f_{4}+3f3+2f_{2}\right)\cdot \rho \left(\pi \left(x\right)+8\right)=\\ =2f_{4}\cdot \left(2x^{3}+3x^{2}+2x\right)+3f_{3}\cdot \left(x^{2}+x\right)+2f_{2}\cdot x\;\left(\mathrm{mod}\;L\right)\\ 20f_{4}\cdot \rho^{3}\left(\pi \left(x\right)+8\right)+\left(150f_{4}+15f_{3}\right)\cdot \rho^{2}\left(\pi \left(x\right)+8\right)+\left(500f_{4}+75f3+10f_{2}\right)\cdot \rho \left(\pi \left(x\right)+8\right)=\\ =10f_{4}\cdot \left(2x^{3}+15x^{2}+50x\right)+15f_{3}\cdot \left(x^{2}+5x\right)+10f_{2}\cdot x\;\left(\mathrm{mod}\;L\right). \end{array}\right. \end{array}$$

For $L=16\cdot k_{L}\cdot \Psi$, $f_{4}=\Psi$, $f_{3}=k_{3,f}\cdot 2\Psi$, $k_{3,f}\in \left\{0,1,2,3\right\}$, $f_{2}=\left(2k_{2,f}\cdot k_{L}-1\right)\cdot \Psi$, $k_{2,f}\in \left\{1,2,3,4\right\}$, $k_{L}\in \left\{1,3\right\}$, equations from (21) become

(22) $$\begin{array}{c} \left\{\begin{array}{c} 4\Psi \cdot \rho^{3}\left(\pi \left(x\right)+2\right)+2\Psi \cdot \left(3+3k_{3,f}\right)\cdot \rho^{2}\left(\pi \left(x\right)+2\right)+2\Psi \cdot \left(2+3k_{3,f}+2k_{2,f}\cdot k_{L}-1\right)\cdot \rho \left(\pi \left(x\right)+2\right)=\\ =2\Psi \cdot \left(2x^{3}+3x^{2}+2x\right)+2\Psi \cdot 3k_{3,f}\cdot \left(x^{2}+x\right)+2\Psi \cdot \left(2k_{2,f}\cdot k_{L}-1\right)\cdot x\;\left(\mathrm{mod}\;16\cdot k_{L}\cdot \Psi \right)\\ 20\Psi \cdot \rho^{3}\left(\pi \left(x\right)+2\right)+2\Psi \cdot \left(75+15k_{3,f}\right)\cdot \rho^{2}\left(\pi \left(x\right)+2\right)+2\Psi \cdot (250+75k_{3,f}+\\ +5\cdot \left(2k_{2,f}\cdot k_{L}-1\right))\cdot \rho \left(\pi \left(x\right)+2\right)=10\Psi \cdot \left(2x^{3}+15x^{2}+50x\right)+2\Psi \cdot 15k_{3,f}\cdot \left(x^{2}+5x\right)+\\ +5\cdot \left(2k_{2,f}\cdot k_{L}-1\right)\cdot 2\Psi \cdot x\;\left(\mathrm{mod}\;16\cdot k_{L}\cdot \Psi \right)\\ 4\Psi \cdot \rho^{3}\left(\pi \left(x\right)+4\right)+2\Psi \cdot \left(3+3k_{3,f}\right)\cdot \rho^{2}\left(\pi \left(x\right)+4\right)+2\Psi \cdot \left(2+3k_{3,f}+2f_{2}\right)\cdot \rho \left(\pi \left(x\right)+4\right)=\\ =2\Psi \cdot \left(2x^{3}+3x^{2}+2x\right)+2\Psi \cdot 3k_{3,f}\cdot \left(x^{2}+x\right)+2\Psi \cdot \left(2k_{2,f}\cdot k_{L}-1\right)\cdot x\;\left(\mathrm{mod}\;16\cdot k_{L}\cdot \Psi \right)\\ 20\Psi \cdot \rho^{3}\left(\pi \left(x\right)+4\right)+2\Psi \cdot \left(75+15k_{3,f}\right)\cdot \rho^{2}\left(\pi \left(x\right)+4\right)+2\Psi \cdot \left(250+75k_{3,f}+5\cdot \left(2k_{2,f}\cdot k_{L}-1\right)\right)\cdot \\ \rho \left(\pi \left(x\right)+4\right)=10\Psi \cdot \left(2x^{3}+15x^{2}+50x\right)+2\Psi \cdot 15k_{3,f}\cdot \left(x^{2}+5x\right)+\\ +5\cdot \left(2k_{2,f}\cdot k_{L}-1\right)\cdot 2\Psi \cdot x\;\left(\mathrm{mod}\;16\cdot k_{L}\cdot \Psi \right)\\ 4\Psi \cdot \rho^{3}\left(\pi \left(x\right)+8\right)+2\Psi \cdot \left(3+3k_{3,f}\right)\cdot \rho^{2}\left(\pi \left(x\right)+8\right)+2\Psi \cdot \left(2+3k_{3,f}+2f_{2}\right)\cdot \rho \left(\pi \left(x\right)+8\right)=\\ =2\Psi \cdot \left(2x^{3}+3x^{2}+2x\right)+2\Psi \cdot 3k_{3,f}\cdot \left(x^{2}+x\right)+2\Psi \cdot \left(2k_{2,f}\cdot k_{L}-1\right)\cdot x\;\left(\mathrm{mod}\;16\cdot k_{L}\cdot \Psi \right)\\ 20\Psi \cdot \rho^{3}\left(\pi \left(x\right)+8\right)+2\Psi \cdot \left(75+15k_{3,f}\right)\cdot \rho^{2}\left(\pi \left(x\right)+8\right)+2\Psi \cdot \left(250+75k_{3,f}+5\cdot \left(2k_{2,f}\cdot k_{L}-1\right)\right)\cdot \\ \rho \left(\pi \left(x\right)+8\right)=10\Psi \cdot \left(2x^{3}+15x^{2}+50x\right)+2\Psi \cdot 15k_{3,f}\cdot \left(x^{2}+5x\right)+\\ +5\cdot \left(2k_{2,f}\cdot k_{L}-1\right)\cdot 2\Psi \cdot x\;\left(\mathrm{mod}\;16\cdot k_{L}\cdot \Psi \right). \end{array}\right. \end{array}$$

Equations from (22) are fulfilled if and only if

(23) $$\left\{\begin{array}{c} 2\cdot \rho^{3}\left(\pi \left(x\right)+2\right)+3\cdot \left(k_{3,f}+1\right)\cdot \rho^{2}\left(\pi \left(x\right)+2\right)+\left(3k_{3,f}+2k_{2,f}k_{L}+1\right)\cdot \rho \left(\pi \left(x\right)+2\right)=\\ =3k_{3,f}\cdot \left(x^{2}+x\right)+\left(2k_{2,f}\cdot k_{L}-1\right)\cdot x+\left(2x^{3}+3x^{2}+2x\right)\;\left(\mathrm{mod}\;8\cdot k_{L}\right)\\ 10\cdot \rho^{3}\left(\pi \left(x\right)+2\right)+15\cdot \left(k_{3,f}+3\right)\cdot \rho^{2}\left(\pi \left(x\right)+2\right)+5\cdot \left(15k_{3,f}+2k_{2,f}\cdot k_{L}-1+50\right)\cdot \rho \left(\pi \left(x\right)+2\right)=\\ =15k_{3,f}\cdot \left(x^{2}+5x\right)+5\cdot \left(2k_{2,f}\cdot k_{L}-1\right)\cdot x+5\cdot \left(2x^{3}+15x^{2}+50x\right)\;\left(\mathrm{mod}\;8\cdot k_{L}\right)\\ 2\cdot \rho^{3}\left(\pi \left(x\right)+4\right)+3\cdot \left(k_{3,f}+1\right)\cdot \rho^{2}\left(\pi \left(x\right)+4\right)+\left(3k_{3,f}+2k_{2,f}\cdot k_{L}+1\right)\cdot \rho \left(\pi \left(x\right)+4\right)=\\ =3k_{3,f}\cdot \left(x^{2}+x\right)+\left(2k_{2,f}\cdot k_{L}-1\right)\cdot x+\left(2x^{3}+3x^{2}+2x\right)\;\left(\mathrm{mod}\;8\cdot k_{L}\right)\\ 10\cdot \rho^{3}\left(\pi \left(x\right)+4\right)+15\cdot \left(k_{3,f}+3\right)\cdot \rho^{2}\left(\pi \left(x\right)+4\right)+\left(250+75k_{3,f}+5\cdot \left(2k_{2,f}\cdot k_{L}-1\right)\right)\cdot \rho \left(\pi \left(x\right)+4\right)=\\ =15k_{3,f}\cdot \left(x^{2}+5x\right)+5\cdot \left(2k_{2,f}\cdot k_{L}-1\right)\cdot x+5\cdot \left(2x^{3}+15x^{2}+50x\right)\;\left(\mathrm{mod}\;8\cdot k_{L}\right)\\ 2\cdot \rho^{3}\left(\pi \left(x\right)+8\right)+3\cdot \left(k_{3,f}+1\right)\cdot \rho^{2}\left(\pi \left(x\right)+8\right)+\left(3k_{3,f}+2k_{2,f}\cdot k_{L}+1\right)\cdot \rho \left(\pi \left(x\right)+8\right)=\\ =3k_{3,f}\cdot \left(x^{2}+x\right)+\left(2k_{2,f}\cdot k_{L}-1\right)\cdot x+\left(2x^{3}+3x^{2}+2x\right)\;\left(\mathrm{mod}\;8\cdot k_{L}\right)\\ 10\cdot \rho^{3}\left(\pi \left(x\right)+8\right)+15\cdot \left(k_{3,f}+3\right)\cdot \rho^{2}\left(\pi \left(x\right)+8\right)+5\cdot \left(15k_{3,f}+2k_{2,f}\cdot k_{L}-1+50\right)\cdot \rho \left(\pi \left(x\right)+8\right)=\\ =15k_{3,f}\cdot \left(x^{2}+5x\right)+5\cdot \left(2k_{2,f}\cdot k_{L}-1\right)\cdot x+5\cdot \left(2x^{3}+15x^{2}+50x\right)\;\left(\mathrm{mod}\;8\cdot k_{L}\right). \end{array}\right.$$

For x=0, equations from (20) become

(24) $$\begin{array}{c} \left\{\begin{array}{c} 2\cdot \rho^{3}\left(2\right)+3\cdot \left(k_{3,f}+1\right)\cdot \rho^{2}\left(2\right)+\left(3k_{3,f}+2k_{2,f}k_{L}+1\right)\cdot \rho \left(2\right)=0\;\left(\mathrm{mod}\;8\cdot k_{L}\right)\\ 10\cdot \rho^{3}\left(2\right)+15\cdot \left(k_{3,f}+3\right)\cdot \rho^{2}\left(2\right)+5\cdot \left(15k_{3,f}+2k_{2,f}\cdot k_{L}-1+50\right)\cdot \rho \left(2\right)=0\;\left(\mathrm{mod}\;8\cdot k_{L}\right)\\ 2\cdot \rho^{3}\left(4\right)+3\cdot \left(k_{3,f}+1\right)\cdot \rho^{2}\left(4\right)+\left(3k_{3,f}+2k_{2,f}\cdot k_{L}+1\right)\cdot \rho \left(4\right)=0\;\left(\mathrm{mod}\;8\cdot k_{L}\right)\\ 10\cdot \rho^{3}\left(4\right)+15\cdot \left(k_{3,f}+3\right)\cdot \rho^{2}\left(4\right)+\left(250+75k_{3,f}+5\cdot \left(2k_{2,f}\cdot k_{L}-1\right)\right)\cdot \rho \left(4\right)=0\;\left(\mathrm{mod}\;8\cdot k_{L}\right)\\ 2\cdot \rho^{3}\left(8\right)+3\cdot \left(k_{3,f}+1\right)\cdot \rho^{2}\left(8\right)+\left(3k_{3,f}+2k_{2,f}\cdot k_{L}+1\right)\cdot \rho \left(8\right)=0\;\left(\mathrm{mod}\;8\cdot k_{L}\right)\\ 10\cdot \rho^{3}\left(8\right)+15\cdot \left(k_{3,f}+3\right)\cdot \rho^{2}\left(8\right)+5\cdot \left(15k_{3,f}+2k_{2,f}\cdot k_{L}-1+50\right)\cdot \rho \left(8\right)=0\;\left(\mathrm{mod}\;8\cdot k_{L}\right) \end{array}\right. \end{array}$$

or

(25) $$\begin{array}{c} \left\{\begin{array}{c} \rho \left(2\right)/2\cdot \left(2\rho^{2}\left(2\right)+3\rho \left(2\right)+1+3k_{3,f}\cdot \left(\rho \left(2\right)+1\right)+2k_{2,f}\cdot k_{L}\right)=0\;\left(\mathrm{mod}\;4\cdot k_{L}\right)\\ 5\cdot \rho \left(2\right)/2\cdot \left(2\rho^{2}\left(2\right)+3\cdot 5\cdot \rho \left(2\right)+49+3k_{3,f}\cdot \left(\rho \left(2\right)+5\right)+2k_{2,f}\cdot k_{L}\right)=0\;\left(\mathrm{mod}\;4\cdot k_{L}\right)\\ \rho \left(4\right)/4\cdot \left(2\rho^{2}\left(4\right)+3\rho \left(4\right)+1+3k_{3,f}\cdot \left(\rho \left(4\right)+1\right)+2k_{2,f}\cdot k_{L}\right)=0\;\left(\mathrm{mod}\;2\cdot k_{L}\right)\\ 5\cdot \rho \left(4\right)/4\cdot \left(2\rho^{2}\left(4\right)+3\cdot 5\cdot \rho \left(4\right)+49+3k_{3,f}\cdot \left(\rho \left(4\right)+5\right)+2k_{2,f}\cdot k_{L}\right)=0\;\left(\mathrm{mod}\;2\cdot k_{L}\right)\\ \rho \left(8\right)/8\cdot \left(2\rho^{2}\left(8\right)+3\rho \left(8\right)+1+3k_{3,f}\cdot \left(\rho \left(8\right)+1\right)+2k_{2,f}\cdot k_{L}\right)=0\;\left(\mathrm{mod}\;k_{L}\right)\\ 5\cdot \rho \left(8\right)/8\cdot \left(2\rho^{2}\left(8\right)+3\cdot 5\cdot \rho \left(8\right)+49+3k_{3,f}\cdot \left(\rho \left(8\right)+5\right)+2k_{2,f}\cdot k_{L}\right)=0\;\left(\mathrm{mod}\;k_{L}\right). \end{array}\right. \end{array}$$

For $k_{L}=1$ and $k_{2,f}^{\prime }=2k_{2,f}-1$, equations from (25) are fulfilled if and only if

(26) $$\begin{array}{c} \left\{\begin{array}{c} \left(2\rho^{2}\left(2\right)+3\rho \left(2\right)+2\right)+3k_{3,f}\cdot \left(\rho \left(2\right)+1\right)+k_{2,f}^{\prime }=0\;\left(\mathrm{mod}\;4\right)\\ \left(2\rho^{2}\left(2\right)+3\cdot 5\cdot \rho \left(2\right)+2\cdot 5^{2}\right)+3k_{3,f}\cdot \left(\rho \left(2\right)+5\right)+k_{2,f}^{\prime }=0\;\left(\mathrm{mod}\;4\right)\\ \left(2\rho^{2}\left(4\right)+3\rho \left(4\right)+2\right)+3k_{3,f}\cdot \left(\rho \left(4\right)+1\right)+k_{2,f}^{\prime }=0\;\left(\mathrm{mod}\;2\right)\\ \left(2\rho^{2}\left(4\right)+3\cdot 5\cdot \rho \left(4\right)+2\cdot 5^{2}\right)+3k_{3,f}\cdot \left(\rho \left(4\right)+5\right)+k_{2,f}^{\prime }=0\;\left(\mathrm{mod}\;2\right) \end{array}\right. \end{array}$$

or

(27) $$\begin{array}{c} \left\{\begin{array}{c} 2\rho_{1}+2+2k_{3,f}\cdot \rho_{1}+3k_{3,f}+k_{2,f}^{\prime }=0\;\left(\mathrm{mod}\;4\right)\\ k_{3,f}+k_{2,f}^{\prime }=0\;\left(\mathrm{mod}\;2\right) \end{array}\right. \end{array}$$

or

(28) $$\begin{array}{c} \left\{\begin{array}{c} 2\rho_{1}\cdot \left(k_{3,f}+1\right)+2+3k_{3,f}+k_{2,f}^{\prime }=0\;\left(\mathrm{mod}\;4\right)\\ k_{3,f}+k_{2,f}^{\prime }=0\;\left(\mathrm{mod}\;2\right). \end{array}\right. \end{array}$$

Equations from (28) are fulfilled if and only if $k_{3,f}=1$ and $k_{2,f}^{\prime }\in \left\{3,7\right\}$, or $k_{3,f}=3$ and $k_{2,f}^{\prime }\in \left\{1,5\right\}$.

For $k_{L}=3$, equations from (25) are fulfilled if and only if

(29) $$\begin{array}{c} \left\{\begin{array}{c} \rho \left(2\right)/2\cdot \left(2\rho^{2}\left(2\right)+3\rho \left(2\right)+1+3k_{3,f}\cdot \left(\rho \left(2\right)+1\right)+6k_{2,f}\right)=0\;\left(\mathrm{mod}\;12\right)\\ \rho \left(4\right)/4\cdot \left(2\rho^{2}\left(4\right)+3\rho \left(4\right)+1+3k_{3,f}\cdot \left(\rho \left(4\right)+1\right)+6k_{2,f}\right)=0\;\left(\mathrm{mod}\;6\right)\\ \rho \left(8\right)/8\cdot \left(2\rho^{2}\left(8\right)+3\rho \left(8\right)+1+3k_{3,f}\cdot \left(\rho \left(8\right)+1\right)+6k_{2,f}\right)=0\;\left(\mathrm{mod}\;3\right) \end{array}\right. \end{array}$$

or

(30) $$\left\{\begin{array}{c} \rho \left(2\right)/2\cdot \left(2\rho^{2}\left(2\right)+3\rho \left(2\right)+1+3k_{3,f}\cdot \left(\rho \left(2\right)+1\right)+6k_{2,f}\right)=0\;\left(\mathrm{mod}\;12\right)\\ \rho \left(4\right)/4\cdot \left(2\rho^{2}\left(4\right)+1+3k_{3,f}\right)=0\;\left(\mathrm{mod}\;6\right)\\ \rho \left(8\right)/8\cdot \left(2\rho^{2}\left(8\right)+1\right)=0\;\left(\mathrm{mod}\;3\right). \end{array}\right.$$

With $\rho_{2}=\left(6k_{2,\rho }-1\right)\cdot \Psi$, $\rho_{3}=k_{3,\rho }\cdot 2\Psi$, and $\rho_{4}=\Psi$, we have

(31) $$\left\{\begin{array}{c} \rho \left(2\right)/2=\rho_{1}+8\cdot k_{3,\rho }\cdot \Psi +6\cdot \Psi \;\left(\mathrm{mod}\;12\right)\\ \rho \left(4\right)/4=\rho_{1}+2\cdot k_{3,\rho }\cdot \Psi \;\left(\mathrm{mod}\;6\right)\\ \rho \left(8\right)/8=\rho_{1}+2\cdot k_{3,\rho }\cdot \Psi \;\left(\mathrm{mod}\;3\right) \end{array}\right.$$

and

(32) $$\left\{\begin{array}{c} 2\cdot \rho^{2}\left(2\right)=8\cdot {\left(\rho_{1}+8\cdot k_{3,\rho }\cdot \Psi +6\cdot \Psi \right)}^{2}\;\left(\mathrm{mod}\;12\right)\\ 2\cdot \rho^{2}\left(4\right)=2\cdot {\left(\rho_{1}+2\cdot k_{3,\rho }\cdot \Psi \right)}^{2}\;\left(\mathrm{mod}\;6\right)\\ 2\cdot \rho^{2}\left(8\right)=2\cdot {\left(\rho_{1}+2\cdot k_{3,\rho }\cdot \Psi \right)}^{2}\;\left(\mathrm{mod}\;3\right). \end{array}\right.$$

With (31) and (32), (30) is equivalent to

(33) $$\left\{\begin{array}{c} 8\cdot {\left(\rho_{1}+8\cdot k_{3,\rho }\cdot \Psi +6\cdot \Psi \right)}^{3}+6\cdot \left(k_{3,f}+1\right)\cdot {\left(\rho_{1}+8\cdot k_{3,\rho }\cdot \Psi +6\cdot \Psi \right)}^{2}+\\ +\left(3k_{3,f}+6k_{2,f}+1\right)\cdot \left(\rho_{1}+8\cdot k_{3,\rho }\cdot \Psi +6\cdot \Psi \right)=0\;\left(\mathrm{mod}\;12\right)\\ 2\cdot {\left(\rho_{1}+2\cdot k_{3,\rho }\cdot \Psi \right)}^{3}+\left(3k_{3,f}+1\right)\cdot \left(\rho_{1}+2\cdot k_{3,\rho }\cdot \Psi \right)=0\;\left(\mathrm{mod}\;6\right)\\ 2\cdot {\left(\rho_{1}+2\cdot k_{3,\rho }\cdot \Psi \right)}^{3}+\left(\rho_{1}+2\cdot k_{3,\rho }\cdot \Psi \right)=0\;\left(\mathrm{mod}\;3\right). \end{array}\right.$$

By exhaustive searching by means software programs, it can be verified that equations from (33) are fulfilled if and only if $k_{3,f}=1$ and $k_{2,f}\in \left\{2,4\right\}$, or $k_{3,f}=3$ and $k_{2,f}\in \left\{1,3\right\}$.

For x=1, equations from (20) become

(34) $$\begin{array}{c} \left\{\begin{array}{c} \rho \left(\pi \left(1\right)+2\right)\cdot (2f_{4}\cdot \left(2\rho^{2}\left(\pi \left(1\right)+2\right)+3\rho \left(\pi \left(1\right)+2\right)+2\right)+\\ +3f_{3}\cdot \left(\rho \left(\pi \left(1\right)+2\right)+1\right)+2f_{2})=14f_{4}+6f_{3}+2f_{2}\;\left(\mathrm{mod}\;L\right)\\ 5\cdot \rho \left(\pi \left(1\right)+2\right)\cdot (2f_{4}\cdot \left(2\rho^{2}\left(\pi \left(1\right)+2\right)+3\cdot 5\cdot \rho \left(\pi \left(1\right)+2\right)+2\cdot 5^{2}\right)+\\ +3f_{3}\cdot \left(\rho \left(\pi \left(1\right)+2\right)+5\right)+2f_{2})=5\cdot \left(134f_{4}+18f_{3}+2f_{2}\right)\;\left(\mathrm{mod}\;L\right)\\ \rho \left(\pi \left(1\right)+4\right)\cdot (2f_{4}\cdot \left(2\rho^{2}\left(\pi \left(1\right)+4\right)+3\rho \left(\pi \left(1\right)+4\right)+2\right)+\\ +3f_{3}\cdot \left(\rho \left(\pi \left(1\right)+4\right)+1\right)+2f_{2})=14f_{4}+6f_{3}+2f_{2}\;\left(\mathrm{mod}\;L\right)\\ 5\cdot \rho \left(\pi \left(1\right)+4\right)\cdot (2f_{4}\cdot \left(2\rho^{2}\left(\pi \left(1\right)+4\right)+3\cdot 5\cdot \rho \left(\pi \left(1\right)+4\right)+2\cdot 5^{2}\right)+\\ +3f_{3}\cdot \left(\rho \left(\pi \left(1\right)+4\right)+5\right)+2f_{2})=5\cdot \left(134f_{4}+18f_{3}+2f_{2}\right)\;\left(\mathrm{mod}\;L\right)\\ \rho \left(\pi \left(1\right)+8\right)\cdot (2f_{4}\cdot \left(2\rho^{2}\left(\pi \left(1\right)+8\right)+3\rho \left(\pi \left(1\right)+8\right)+2\right)+\\ +3f_{3}\cdot \left(\rho \left(\pi \left(1\right)+8\right)+1\right)+2f_{2})=14f_{4}+6f_{3}+2f_{2}\;\left(\mathrm{mod}\;L\right)\\ 5\cdot \rho \left(\pi \left(1\right)+8\right)\cdot (2f_{4}\cdot \left(2\rho^{2}\left(\pi \left(1\right)+8\right)+3\cdot 5\cdot \rho \left(\pi \left(1\right)+8\right)+2\cdot 5^{2}\right)+\\ +3f_{3}\cdot \left(\rho \left(\pi \left(1\right)+8\right)+5\right)+2f_{2})=5\cdot \left(134f_{4}+18f_{3}+2f_{2}\right)\;\left(\mathrm{mod}\;L\right). \end{array}\right. \end{array}$$

For $L=16\cdot k_{L}\cdot \Psi$, $f_{4}=\Psi$, $f_{3}=k_{3,f}\cdot 2\Psi$, $k_{3,f}\in \left\{0,1,2,3\right\}$, $f_{2}=\left(2k_{2,f}\cdot k_{L}-1\right)\cdot \Psi$, $k_{2,f}\in \left\{1,2,3,4\right\}$, $k_{L}\in \left\{1,3\right\}$, equations from (34) become

(35) $$\begin{array}{c} \left\{\begin{array}{c} \rho \left(\pi \left(1\right)+2\right)\cdot 2\Psi \cdot (2\rho^{2}\left(\pi \left(1\right)+2\right)+3\rho \left(\pi \left(1\right)+2\right)+1+\\ +3k_{3,f}\cdot \left(\rho \left(\pi \left(1\right)+2\right)+1\right)+2k_{2,f}k_{L})-2\Psi \cdot \left(6+6k_{3,f}+2k_{2,f}k_{L}\right)=0\;\left(\mathrm{mod}\;16\cdot k_{L}\cdot \Psi \right)\\ 5\cdot 2\Psi \cdot \rho \left(\pi \left(1\right)+2\right)\cdot (2\rho^{2}\left(\pi \left(1\right)+2\right)+3\cdot 5\cdot \rho \left(\pi \left(1\right)+2\right)+2\cdot 5^{2}-1+\\ +3k_{3,f}\cdot \left(\rho \left(\pi \left(1\right)+2\right)+5\right)+2k_{2,f}k_{L})-5\cdot 2\Psi \cdot \left(66+18k_{3,f}+2k_{2,f}k_{L}\right)=0\;\left(\mathrm{mod}\;16\cdot k_{L}\cdot \Psi \right)\\ \rho \left(\pi \left(1\right)+4\right)\cdot 2\Psi \cdot (2\rho^{2}\left(\pi \left(1\right)+4\right)+3\rho \left(\pi \left(1\right)+4\right)+1+\\ +3k_{3,f}\cdot \left(\rho \left(\pi \left(1\right)+4\right)+1\right)+2k_{2,f}k_{L})-2\Psi \cdot \left(6+6k_{3,f}+2k_{2,f}k_{L}\right)=0\;\left(\mathrm{mod}\;16\cdot k_{L}\cdot \Psi \right)\\ 5\cdot \rho \left(\pi \left(1\right)+4\right)\cdot 2\Psi \cdot (2\rho^{2}\left(\pi \left(1\right)+4\right)+3\cdot 5\cdot \rho \left(\pi \left(1\right)+4\right)+2\cdot 5^{2}-1+\\ +3k_{3,f}\cdot \left(\rho \left(\pi \left(1\right)+4\right)+5\right)+2k_{2,f}k_{L})-5\cdot 2\Psi \cdot \left(66+18k_{3,f}+2k_{2,f}k_{L}\right)=0\;\left(\mathrm{mod}\;16\cdot k_{L}\cdot \Psi \right)\\ \rho \left(\pi \left(1\right)+8\right)\cdot 2\Psi \cdot (2\rho^{2}\left(\pi \left(1\right)+8\right)+3\rho \left(\pi \left(1\right)+8\right)+1+\\ +3k_{3,f}\cdot \left(\rho \left(\pi \left(1\right)+8\right)+1\right)+2k_{2,f}k_{L})-2\Psi \cdot \left(6+6k_{3,f}+2k_{2,f}k_{L}\right)=0\;\left(\mathrm{mod}\;16\cdot k_{L}\cdot \Psi \right)\\ 5\cdot \rho \left(\pi \left(1\right)+8\right)\cdot 2\Psi \cdot (2\rho^{2}\left(\pi \left(1\right)+8\right)+3\cdot 5\cdot \rho \left(\pi \left(1\right)+8\right)+2\cdot 5^{2}-1+\\ +3k_{3,f}\cdot \left(\rho \left(\pi \left(1\right)+8\right)+5\right)+2k_{2,f}k_{L})-5\cdot 2\Psi \cdot \left(66+18k_{3,f}+2k_{2,f}k_{L}\right)=0\;\left(\mathrm{mod}\;16\cdot k_{L}\cdot \Psi \right). \end{array}\right. \end{array}$$

Equations from (35) are fulfilled if and only if

(36) $$\begin{array}{c} \left\{\begin{array}{c} \rho \left(\pi \left(1\right)+2\right)\cdot \left(2\rho^{2}\left(\pi \left(1\right)+2\right)+3\rho \left(\pi \left(1\right)+2\right)+1+3k_{3,f}\cdot \left(\rho \left(\pi \left(1\right)+2\right)+1\right)+2k_{2,f}k_{L}\right)-\\ -\left(6+6k_{3,f}+2k_{2,f}k_{L}\right)=0\;\left(\mathrm{mod}\;8\cdot k_{L}\right)\\ 5\cdot \rho \left(\pi \left(1\right)+2\right)\cdot (2\rho^{2}\left(\pi \left(1\right)+2\right)+3\cdot 5\cdot \rho \left(\pi \left(1\right)+2\right)+2\cdot 5^{2}-1+\\ +3k_{3,f}\cdot \left(\rho \left(\pi \left(1\right)+2\right)+5\right)+2k_{2,f}k_{L})-5\cdot \left(66+18k_{3,f}+2k_{2,f}k_{L}\right)=0\;\left(\mathrm{mod}\;8\cdot k_{L}\right)\\ \rho \left(\pi \left(1\right)+4\right)\cdot \left(2\rho^{2}\left(\pi \left(1\right)+4\right)+3\rho \left(\pi \left(1\right)+4\right)+1+3k_{3,f}\cdot \left(\rho \left(\pi \left(1\right)+4\right)+1\right)+2k_{2,f}k_{L}\right)-\\ -\left(6+6k_{3,f}+2k_{2,f}k_{L}\right)=0\;\left(\mathrm{mod}\;8\cdot k_{L}\right)\\ 5\cdot \rho \left(\pi \left(1\right)+4\right)\cdot (2\rho^{2}\left(\pi \left(1\right)+4\right)+3\cdot 5\cdot \rho \left(\pi \left(1\right)+4\right)+2\cdot 5^{2}-1+\\ +3k_{3,f}\cdot \left(\rho \left(\pi \left(1\right)+4\right)+5\right)+2k_{2,f}k_{L})-5\cdot \left(66+18k_{3,f}+2k_{2,f}k_{L}\right)=0\;\left(\mathrm{mod}\;8\cdot k_{L}\right)\\ \rho \left(\pi \left(1\right)+8\right)\cdot \left(2\rho^{2}\left(\pi \left(1\right)+8\right)+3\rho \left(\pi \left(1\right)+8\right)+1+3k_{3,f}\cdot \left(\rho \left(\pi \left(1\right)+8\right)+1\right)+2k_{2,f}k_{L}\right)-\\ -\left(6+6k_{3,f}+2k_{2,f}k_{L}\right)=0\;\left(\mathrm{mod}\;8\cdot k_{L}\right)\\ 5\cdot \rho \left(\pi \left(1\right)+8\right)\cdot (2\rho^{2}\left(\pi \left(1\right)+8\right)+3\cdot 5\cdot \rho \left(\pi \left(1\right)+8\right)+2\cdot 5^{2}-1+\\ +3k_{3,f}\cdot \left(\rho \left(\pi \left(1\right)+8\right)+5\right)+2k_{2,f}k_{L})-5\cdot \left(66+18k_{3,f}+2k_{2,f}k_{L}\right)=0\;\left(\mathrm{mod}\;8\cdot k_{L}\right). \end{array}\right. \end{array}$$

For $k_{L}=1$ and $k_{2,f}^{\prime }=2k_{2,f}-1$, equations from (36) are fulfilled if and only if

(37) $$\begin{array}{c} \left\{\begin{array}{c} \rho \left(\pi \left(1\right)+2\right)\cdot (2\rho^{2}\left(\pi \left(1\right)+2\right)+3\rho \left(\pi \left(1\right)+2\right)+2+\\ +3k_{3,f}\cdot \left(\rho \left(\pi \left(1\right)+2\right)+1\right)+k_{2,f}^{\prime })+\left(1+2k_{3,f}+7k_{2,f}^{\prime }\right)=0\;\left(\mathrm{mod}\;8\right)\\ 5\cdot \rho \left(\pi \left(1\right)+2\right)\cdot (2\rho^{2}\left(\pi \left(1\right)+2\right)+7\cdot \rho \left(\pi \left(1\right)+2\right)+2+\\ +3k_{3,f}\cdot \left(\rho \left(\pi \left(1\right)+2\right)+5\right)+k_{2,f}^{\prime })+\left(1+6k_{3,f}+3k_{2,f}^{\prime }\right)=0\;\left(\mathrm{mod}\;8\right)\\ \rho \left(\pi \left(1\right)+4\right)\cdot (2\rho^{2}\left(\pi \left(1\right)+4\right)+3\rho \left(\pi \left(1\right)+4\right)+2+\\ +3k_{3,f}\cdot \left(\rho \left(\pi \left(1\right)+4\right)+1\right)+k_{2,f}^{\prime })+\left(1+2k_{3,f}+7k_{2,f}^{\prime }\right)=0\;\left(\mathrm{mod}\;8\right)\\ 5\cdot \rho \left(\pi \left(1\right)+4\right)\cdot (2\rho^{2}\left(\pi \left(1\right)+4\right)+7\cdot \rho \left(\pi \left(1\right)+4\right)+2+\\ +3k_{3,f}\cdot \left(\rho \left(\pi \left(1\right)+4\right)+5\right)+k_{2,f}^{\prime })+\left(1+6k_{3,f}+3k_{2,f}^{\prime }\right)=0\;\left(\mathrm{mod}\;8\right)\\ \rho \left(\pi \left(1\right)+8\right)\cdot (2\rho^{2}\left(\pi \left(1\right)+8\right)+3\rho \left(\pi \left(1\right)+8\right)+2+\\ +3k_{3,f}\cdot \left(\rho \left(\pi \left(1\right)+8\right)+1\right)+k_{2,f}^{\prime })+\left(1+2k_{3,f}+7k_{2,f}^{\prime }\right)=0\;\left(\mathrm{mod}\;8\right)\\ 5\cdot \rho \left(\pi \left(1\right)+8\right)\cdot (2\rho^{2}\left(\pi \left(1\right)+8\right)+7\cdot \rho \left(\pi \left(1\right)+8\right)+2+\\ +3k_{3,f}\cdot \left(\rho \left(\pi \left(1\right)+8\right)+5\right)+k_{2,f}^{\prime })+\left(1+6k_{3,f}+3k_{2,f}^{\prime }\right)=0\;\left(\mathrm{mod}\;8\right). \end{array}\right. \end{array}$$

We have

$$\rho \left(\pi \left(1\right)+2\right)\;\left(\mathrm{mod}\;8\right)=1+\rho \left(2\right)+2\cdot \pi \left(1\right)\cdot (2\rho_{4}\cdot \left(2\pi^{2}\left(1\right)+2\cdot 2^{2}+3\cdot 2\cdot \pi \left(1\right)\right)+$$

$$+3\rho_{3}\cdot \left(\pi \left(1\right)+2\right)+2\rho_{2})\;\left(\mathrm{mod}\;8\right)=$$

$$=1+2\cdot \left(\rho_{1}+2\rho_{2}\right)+6\rho_{3}\cdot {\left(f_{1}+f_{2}+f_{3}+f_{4}\right)}^{2}+\left(4\rho_{3}+4\rho_{2}\right)\cdot \left(f_{1}+f_{2}+f_{3}+f_{4}\right)\;\left(\mathrm{mod}\;8\right)=$$

$$=1+2\cdot \left(\rho_{1}+2k_{2,\rho }k_{\Psi }\right)+4k_{3,\rho }k_{\Psi }\cdot {\left(f_{1}+k_{2,f}^{\prime }k_{\Psi }+k_{\Psi }\right)}^{2}+4k_{2,\rho }k_{\Psi }\cdot \left(f_{1}+k_{2,f}^{\prime }k_{\Psi }+k_{\Psi }\right)\;\left(\mathrm{mod}\;8\right)=$$

$$=1+2\cdot \left(\rho_{1}+2k_{2,\rho }k_{\Psi }\right)+4k_{3,\rho }k_{\Psi }\cdot \left(1+{\left(k_{2,f}^{\prime }\right)}^{2}+1+2f_{1}k_{2,f}^{\prime }k_{\Psi }+2f_{1}k_{\Psi }+2k_{2,f}^{\prime }\right)+$$

$$+4f_{1}k_{2,\rho }k_{\Psi }+4k_{2,\rho }\cdot \left(k_{2,f}^{\prime }+1\right)\;\left(\mathrm{mod}\;8\right)=$$

(38) $$\begin{array}{c} =1+2\rho_{1}+4k_{3,\rho }{\left(k_{2,f}^{\prime }\right)}^{2}k_{\Psi }+4k_{2,\rho }\cdot \left(k_{2,f}^{\prime }+1\right)\;\left(\mathrm{mod}\;8\right), \end{array}$$

(39) $$\begin{array}{c} \rho^{2}\left(\pi \left(1\right)+2\right)\;\left(\mathrm{mod}\;8\right)=5+4\rho_{1}\;\left(\mathrm{mod}\;8\right), \end{array}$$

(40) $$\begin{array}{c} \rho^{3}\left(\pi \left(1\right)+2\right)\;\left(\mathrm{mod}\;8\right)=5+6\rho_{1}+4k_{3,\rho }{\left(k_{2,f}^{\prime }\right)}^{2}k_{\Psi }+4k_{2,\rho }\cdot \left(k_{2,f}^{\prime }+1\right)\;\left(\mathrm{mod}\;8\right), \end{array}$$

$$\rho \left(\pi \left(1\right)+4\right)\;\left(\mathrm{mod}\;8\right)=1+\rho \left(4\right)+4\cdot \pi \left(1\right)\cdot (2\rho_{4}\cdot \left(2\pi^{2}\left(1\right)+2\cdot 4^{2}+3\cdot 4\cdot \pi \left(1\right)\right)+$$

(41) $$\begin{array}{c} +3\rho_{3}\cdot \left(\pi \left(1\right)+4\right)+2\rho_{2})\;\left(\mathrm{mod}\;8\right)=1+4\rho_{1}\;\left(\mathrm{mod}\;8\right), \end{array}$$

(42) $$\begin{array}{c} \rho^{2}\left(\pi \left(1\right)+4\right)\;\left(\mathrm{mod}\;8\right)=1\;\left(\mathrm{mod}\;8\right), \end{array}$$

$$\rho \left(\pi \left(1\right)+8\right)\;\left(\mathrm{mod}\;8\right)=1+\rho \left(8\right)+8\cdot \pi \left(1\right)\cdot (2\rho_{4}\cdot \left(2\pi^{2}\left(1\right)+2\cdot 8^{2}+3\cdot 8\cdot \pi \left(1\right)\right)+$$

(43) $$\begin{array}{c} +3\rho_{3}\cdot \left(\pi \left(1\right)+8\right)+2\rho_{2})\;\left(\mathrm{mod}\;8\right)=1\;\left(\mathrm{mod}\;8\right). \end{array}$$

Thus, equations from (36) are equivalent to

(44) $$\begin{array}{c} \left\{\begin{array}{c} 2\rho^{3}\left(\pi \left(1\right)+2\right)+3\cdot \left(k_{3,f}+1\right)\cdot \rho^{2}\left(\pi \left(1\right)+2\right)+\\ +\left(2+3k_{3,f}+k_{2,f}^{\prime }\right)\cdot \rho \left(\pi \left(1\right)+2\right)+\left(1+2k_{3,f}+7k_{2,f}^{\prime }\right)=0\;\left(\mathrm{mod}\;8\right)\\ 2\rho^{3}\left(\pi \left(1\right)+2\right)+\left(7k_{3,f}+3\right)\cdot \rho^{2}\left(\pi \left(1\right)+2\right)+\\ +\left(2+3k_{3,f}+k_{2,f}^{\prime }\right)\cdot \rho \left(\pi \left(1\right)+2\right)+\left(1+6k_{3,f}+3k_{2,f}^{\prime }\right)=0\;\left(\mathrm{mod}\;8\right)\\ \left(1+4\rho_{1}\right)\cdot \left(2+3k_{3,f}+k_{2,f}^{\prime }\right)+\left(6+5k_{3,f}+7k_{2,f}^{\prime }\right)=0\;\left(\mathrm{mod}\;8\right)\\ \left(1+4\rho_{1}\right)\cdot \left(2+3k_{3,f}+5k_{2,f}^{\prime }\right)+\left(6+5k_{3,f}+3k_{2,f}^{\prime }\right)=0\;\left(\mathrm{mod}\;8\right) \end{array}\right. \end{array}$$

or

(45) $$\begin{array}{c} \left\{\begin{array}{c} 2\rho^{3}\left(\pi \left(1\right)+2\right)+3\cdot \left(k_{3,f}+1\right)\cdot \rho^{2}\left(\pi \left(1\right)+2\right)+\\ +\left(2+3k_{3,f}+k_{2,f}^{\prime }\right)\cdot \rho \left(\pi \left(1\right)+2\right)+\left(1+2k_{3,f}+7k_{2,f}^{\prime }\right)=0\;\left(\mathrm{mod}\;8\right)\\ 2\rho^{3}\left(\pi \left(1\right)+2\right)+\left(7k_{3,f}+3\right)\cdot \rho^{2}\left(\pi \left(1\right)+2\right)+\\ +\left(2+3k_{3,f}+k_{2,f}^{\prime }\right)\cdot \rho \left(\pi \left(1\right)+2\right)+\left(1+6k_{3,f}+3k_{2,f}^{\prime }\right)=0\;\left(\mathrm{mod}\;8\right)\\ 4\rho_{1}\cdot \left(k_{3,f}+k_{2,f}^{\prime }\right)=0\;\left(\mathrm{mod}\;8\right). \end{array}\right. \end{array}$$

The third equation from (45) is fulfilled if and only if $k_{3,f}=1$ and $k_{2,f}^{\prime }\in \left\{1,5\right\}$, or $k_{3,f}=3$ and $k_{2,f}^{\prime }\in \left\{3,7\right\}$. It can be verified that these values also fulfill the first two equations from (45).

For $k_{L}=3$, equations from (36) are fulfilled if and only if

(46) $$\left\{\begin{array}{c} \rho \left(\pi \left(1\right)+2\right)\cdot \left(2\rho^{2}\left(\pi \left(1\right)+2\right)+3\rho \left(\pi \left(1\right)+2\right)+1+3k_{3,f}\cdot \left(\rho \left(\pi \left(1\right)+2\right)+1\right)+6k_{2,f}\right)+\\ +18\cdot \left(1+k_{3,f}+k_{2,f}\right)=0\;\left(\mathrm{mod}\;24\right)\\ 5\cdot \rho \left(\pi \left(1\right)+2\right)\cdot (2\rho^{2}\left(\pi \left(1\right)+2\right)+15\cdot \rho \left(\pi \left(1\right)+2\right)+1+\\ +3k_{3,f}\cdot \left(\rho \left(\pi \left(1\right)+2\right)+5\right)+6k_{2,f})+6\cdot \left(1+k_{3,f}+3k_{2,f}\right)=0\;\left(\mathrm{mod}\;24\right)\\ \rho \left(\pi \left(1\right)+4\right)\cdot \left(2\rho^{2}\left(\pi \left(1\right)+4\right)+3\rho \left(\pi \left(1\right)+4\right)+1+3k_{3,f}\cdot \left(\rho \left(\pi \left(1\right)+4\right)+1\right)+6k_{2,f}\right)+\\ +18\cdot \left(1+k_{3,f}+k_{2,f}\right)=0\;\left(\mathrm{mod}\;24\right)\\ 5\cdot \rho \left(\pi \left(1\right)+4\right)\cdot (2\rho^{2}\left(\pi \left(1\right)+4\right)+15\cdot \rho \left(\pi \left(1\right)+4\right)+1+\\ +3k_{3,f}\cdot \left(\rho \left(\pi \left(1\right)+4\right)+5\right)+6k_{2,f})+6\cdot \left(1+k_{3,f}+3k_{2,f}\right)=0\;\left(\mathrm{mod}\;24\right)\\ \rho \left(\pi \left(1\right)+8\right)\cdot \left(2\rho^{2}\left(\pi \left(1\right)+8\right)+3\rho \left(\pi \left(1\right)+8\right)+1+3k_{3,f}\cdot \left(\rho \left(\pi \left(1\right)+8\right)+1\right)+6k_{2,f}\right)+\\ +18\cdot \left(1+k_{3,f}+k_{2,f}\right)=0\;\left(\mathrm{mod}\;24\right)\\ 5\cdot \rho \left(\pi \left(1\right)+8\right)\cdot (2\rho^{2}\left(\pi \left(1\right)+8\right)+15\cdot \rho \left(\pi \left(1\right)+8\right)+1+\\ +3k_{3,f}\cdot \left(\rho \left(\pi \left(1\right)+8\right)+5\right)+6k_{2,f})+6\cdot \left(1+k_{3,f}+3k_{2,f}\right)=0\;\left(\mathrm{mod}\;24\right). \end{array}\right.$$

With

(47) $$\pi \left(1\right)\;\left(\mathrm{mod}\;24\right)=\left(f_{1}+6k_{2,f}k_{\Psi }+2k_{3,f}k_{\Psi }\right)\;\left(\mathrm{mod}\;24\right),$$

we have

$$\rho \left(\pi \left(1\right)+2\right)\;\left(\mathrm{mod}\;24\right)=1+\rho \left(2\right)+2\cdot \pi \left(1\right)\cdot (2\rho_{4}\cdot \left(2\pi^{2}\left(1\right)+2\cdot 2^{2}+3\cdot 2\cdot \pi \left(1\right)\right)+$$

$$+3\rho_{3}\cdot \left(\pi \left(1\right)+2\right)+2\rho_{2})\;\left(\mathrm{mod}\;24\right)=$$

$$=1+2\cdot \left(\rho_{1}+2\rho_{2}+4\rho_{3}+8\rho_{4}\right)+8\rho_{4}\cdot \pi^{3}\left(1\right)+6\rho_{3}\pi^{2}\left(1\right)+\left(8\rho_{4}+12\rho_{3}+4\rho_{2}\right)\cdot \pi \left(1\right)=$$

(48) $$=1+2\rho_{1}+16k_{3,\rho }k_{\Psi }+12k_{\Psi }+8k_{\Psi }\cdot \pi^{3}\left(1\right)+12k_{3,\rho }k_{\Psi }\cdot \pi^{2}\left(1\right)+4k_{\Psi }\cdot \left(f_{1}+2k_{3,f}k_{\Psi }\right)\;\left(\mathrm{mod}\;24\right),$$

$$\rho^{2}\left(\pi \left(1\right)+2\right)\;\left(\mathrm{mod}\;24\right)=1+4\rho_{1}\cdot \left(\rho_{1}+1\right)+8k_{3,\rho }k_{\Psi }\cdot \left(2k_{3,\rho }k_{\Psi }+2\rho_{1}+1\right)+$$

$$+16k_{3,\rho }^{2}\cdot \pi^{6}\left(1\right)+16k_{\Psi }\cdot \pi^{4}\left(1\right)+8k_{\Psi }\cdot \left(2+\rho_{1}+2k_{3,\rho }k_{\Psi }\right)\cdot \pi^{3}\left(1\right)+16k_{\Psi }\cdot \pi^{2}\left(1\right)+$$

(49) $$+8k_{\Psi }\cdot \left(1+2\rho_{1}+k_{3,\rho }k_{\Psi }\right)\cdot \left(f_{1}+2k_{3,f}k_{\Psi }\right)\;\mathrm{mod}\;24),$$

$$\rho^{3}\left(\pi \left(1\right)+2\right)\;\left(\mathrm{mod}\;24\right)=1+2\rho_{1}\cdot \left(4\rho_{1}^{2}+6\rho_{1}+3\right)+4k_{\Psi }\cdot \left(4k_{3,\rho }^{3}k_{\Psi }^{2}+3\right)+$$

(50) $$+8k_{\Psi }^{3}\cdot \pi^{9}\left(1\right)+16k_{\Psi }^{3}\cdot \pi^{3}\left(1\right)+12k_{3,\rho }k_{\Psi }\cdot \pi^{2}\left(1\right)+12k_{\Psi }f_{1}\;\left(\mathrm{mod}\;24\right),$$

$$\rho \left(\pi \left(1\right)+4\right)\;\left(\mathrm{mod}\;24\right)=1+\rho \left(4\right)+4\cdot \pi \left(1\right)\cdot (2\rho_{4}\cdot \left(2\pi^{2}\left(1\right)+2\cdot 4^{2}+3\cdot 4\cdot \pi \left(1\right)\right)+$$

$$+3\rho_{3}\cdot \left(\pi \left(1\right)+4\right)+2\rho_{2})\;\left(\mathrm{mod}\;24\right)=$$

$$=1+\rho \left(4\right)+16\rho_{4}\cdot \pi^{3}\left(1\right)+12\rho_{3}\cdot \pi^{2}\left(1\right)+\left(16\rho_{4}+8\rho_{2}\right)\cdot \pi \left(1\right)\;\left(\mathrm{mod}\;24\right)=$$

$$=1+4\cdot \left(\rho_{1}+4\rho_{2}+16\rho_{3}+16\rho_{4}\right)+16\rho_{4}\cdot {\left(f_{1}+f_{2}+f_{3}+f_{4}\right)}^{3}+12\rho_{3}\cdot {\left(f_{1}+f_{2}+f_{3}+f_{4}\right)}^{2}+$$

$$+\left(16\rho_{4}+8\rho_{2}\right)\cdot \left(f_{1}+f_{2}+f_{3}+f_{4}\right)\;\left(\mathrm{mod}\;24\right)=$$

$$=1+4\cdot \left(\rho_{1}+8k_{3,\rho }k_{\Psi }\right)+16k_{\Psi }\cdot {\left(f_{1}+6k_{2,f}k_{\Psi }+2k_{3,f}k_{\Psi }\right)}^{3}+$$

(51) $$+8k_{\Psi }\cdot \left(f_{1}+2k_{3,f}k_{\Psi }\right)\;\left(\mathrm{mod}\;24\right),$$

$$\rho^{2}\left(\pi \left(1\right)+4\right)\;\left(\mathrm{mod}\;24\right)=1+8\rho_{1}\cdot \left(2\rho_{1}+1\right)+16k_{3,\rho }k_{\Psi }\cdot \left(k_{3,\rho }k_{\Psi }+\rho_{1}+1\right)+$$

$$+16k_{3,\rho }^{2}\cdot \pi^{6}\left(1\right)+16k_{\Psi }^{2}\cdot \pi^{4}\left(1\right)+8k_{\Psi }\cdot \left(1+\rho_{1}+2k_{3,\rho }k_{\Psi }\right)\cdot \pi^{3}\left(1\right)+16k_{\Psi }^{2}\cdot \pi^{2}\left(1\right)+$$

(52) $$+8k_{\Psi }\cdot \left(2+2\rho_{1}+k_{3,\rho }k_{\Psi }\right)\cdot \left(f_{1}+2k_{3,f}k_{\Psi }\right)\;\mathrm{mod}\;24),$$

(53) $$\rho^{3}\left(\pi \left(1\right)+4\right)\;\left(\mathrm{mod}\;24\right)=1+4\rho_{1}\cdot \left(4\rho_{1}^{2}+3\right)+8k_{\Psi }^{3}k_{3,\rho }^{3}+16k_{\Psi }^{3}\cdot \pi^{9}\left(1\right)+8k_{\Psi }^{3}\cdot \pi^{3}\left(1\right)\;\left(\mathrm{mod}\;24\right),$$

$$\rho \left(\pi \left(1\right)+8\right)\;\left(\mathrm{mod}\;24\right)=1+\rho \left(8\right)+8\cdot \pi \left(1\right)\cdot (2\rho_{4}\cdot \left(2\pi^{2}\left(1\right)+2\cdot 8^{2}+3\cdot 8\cdot \pi \left(1\right)\right)+$$

$$+3\rho_{3}\cdot \left(\pi \left(1\right)+8\right)+2\rho_{2})\;\left(\mathrm{mod}\;24\right)=1+\rho \left(8\right)+8\rho_{4}\cdot \pi^{3}\left(1\right)+8\cdot \left(2\rho_{2}+\rho_{4}\right)\cdot \pi \left(1\right)=$$

$$=1+8\cdot \left(\rho_{1}+2k_{3,\rho }k_{\Psi }\right)+8k_{\Psi }\cdot {\left(f_{1}+6k_{2,f}k_{\Psi }+2k_{3,f}k_{\Psi }\right)}^{3}+$$

(54) $$+16k_{\Psi }\cdot \left(f_{1}+2k_{3,f}k_{\Psi }\right)\;\left(\mathrm{mod}\;24\right),$$

$$\rho^{2}\left(\pi \left(1\right)+4\right)\;\left(\mathrm{mod}\;24\right)=1+16\rho_{1}\cdot \left(\rho_{1}+1\right)+8k_{3,\rho }k_{\Psi }\cdot \left(2k_{3,\rho }k_{\Psi }+2\rho_{1}+1\right)+$$

$$+16k_{3,\rho }^{2}\cdot \pi^{6}\left(1\right)+16k_{\Psi }^{2}\cdot \pi^{4}\left(1\right)+8k_{\Psi }\cdot \left(2+\rho_{1}+2k_{3,\rho }k_{\Psi }\right)\cdot \pi^{3}\left(1\right)+16k_{\Psi }^{2}\cdot \pi^{2}\left(1\right)+$$

(55) $$+8k_{\Psi }\cdot \left(1+2\rho_{1}+k_{3,\rho }k_{\Psi }\right)\cdot \left(f_{1}+2k_{3,f}k_{\Psi }\right)\;\mathrm{mod}\;24),$$

(56) $$\rho^{3}\left(\pi \left(1\right)+8\right)\;\left(\mathrm{mod}\;24\right)=1+8\rho_{1}^{3}+16k_{\Psi }^{3}k_{3,\rho }^{3}+8k_{\Psi }^{3}\cdot \pi^{9}\left(1\right)+16k_{\Psi }^{3}\cdot \pi^{3}\left(1\right)\;\left(\mathrm{mod}\;24\right).$$

Taking into account Equations (47)–(56), it can be verified, by exhaustive searching by means of Matlab that equations from system (46) are fulfilled if and only if $k_{3,f}=1$ and $k_{2,f}\in \left\{1,3\right\}$, or $k_{3,f}=3$ and $k_{2,f}\in \left\{2,4\right\}$.

From solutions of (28), (33), (45), and (46), it results that the interleaver pattern from Figure 1 always appears for $x_{1}=0$ or $x_{1}=1$, when $k_{3,f}\in \left\{1,3\right\}$ and $k_{2,f}\in \left\{1,2,3,4\right\}$. For an interleaver pattern as in Figure 1, the weight of the codeword for classical nominal 1/3 rate turbo codes with two RSC codes having generator matrix G=[1,15/13], is equal to 12+4·3+3·4=36, because each of the four error patterns with a weight of three leads to a parity weight of three, and each of the three error patterns with a weight of four leads to a parity weight of four. Because the interleaver pattern from Figure 1 always appears in the previous conditions, it results that the minimum distance is upper bounded by the value of 36. □

**Theorem** **2.** *Let the interleaver length be of the form given in *(5)*. Then, the minimum distance of the classical nominal 1/3 rate turbo code—with two RSC codes concatenated in parallel, having the generator matrix G=[1,15/13] (in octal form), 4-PP interleavers, and fulfilling conditions (6) when $3∤(p_{i}-1)$, with coefficients $f_{4}=\Psi$, $f_{3}=k_{3,f}\cdot 2\Psi$, $f_{2}=\left(2k_{2,f}\cdot k_{L}-1\right)\cdot \Psi$, $k_{L}\in \left\{1,3\right\}$, when $k_{3,f}\in \left\{0,2\right\}$ and $k_{2,f}\in \left\{1,2,3,4\right\}$ or when $k_{3,f}\in \left\{1,3\right\}$ and $k_{2,f}\in \left\{2,4\right\}$—is upper bounded by the value of 28.*

**Figure 2:**
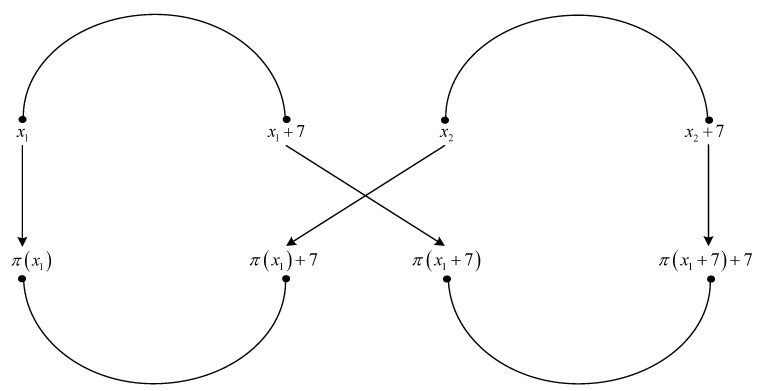
Critical interleaver pattern of size four for 4-PP-based interleavers.

**Figure 3:**
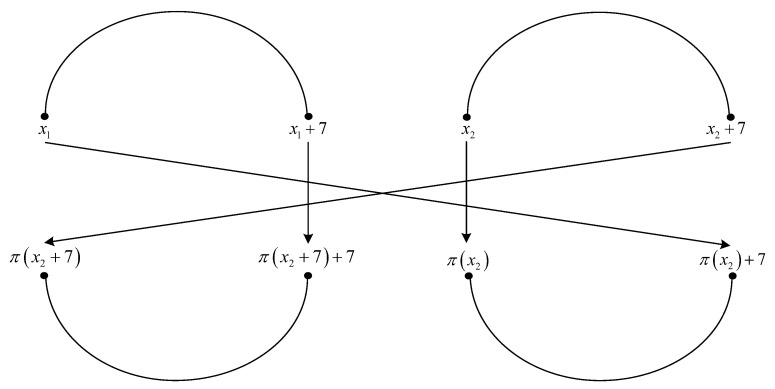
Critical interleaver pattern of size four for 4-PP-based interleavers.

**Proof.**  We consider the interleaver patterns of size four shown in Figure 2 and Figure 3.

The four elements of permutation π(·) indicated in Figure 2 are written in detail below.

(57) $$\begin{array}{c} \left\{\begin{array}{c} x_{1}\to \pi \left(x_{1}\right)\\ x_{1}+7\to \pi \left(x_{1}+7\right)\;\left(\mathrm{mod}\;L\right)\\ x_{2}\to \pi \left(x_{2}\right)=\pi \left(x_{1}\right)+7\;\left(\mathrm{mod}\;L\right)\\ x_{2}+7\to \pi \left(x_{2}+7\right)=\pi \left(x_{1}+7\right)+7\;\left(\mathrm{mod}\;L\right). \end{array}\right. \end{array}$$

Writing $x_{2}=\rho \left(\pi \left(x_{2}\right)\right)=\rho \left(\pi \left(x_{1}\right)+7\right)$ in the fourth equation from (57), with $x_{1}=x$, we have

(58) $$\begin{array}{c} \pi (\rho (\pi (x)+7)+7)=\pi (x+7)+7\;(\mathrm{mod}\;L). \end{array}$$

Equation (58) is equivalent to

$$7\cdot \rho \left(\pi \left(x\right)+7\right)\cdot \left(2f_{4}\cdot \left(2\rho^{2}\left(\pi \left(x\right)+7\right)+3\cdot 7\cdot \rho \left(\pi \left(x\right)+7\right)+2\cdot 7^{2}\right)+3f_{3}\cdot \left(\rho \left(\pi \left(x\right)+7\right)+7\right)+2f_{2}\right)=$$

(59) $$\begin{array}{c} =7\cdot x\cdot \left(2f_{4}\cdot \left(2x^{2}+3\cdot 7\cdot x+2\cdot 7^{2}\right)+3f_{3}\cdot \left(x+7\right)+2f_{2}\right)\;\left(\mathrm{mod}\;L\right) \end{array}$$

or

$$28f_{4}\cdot \rho^{3}\left(\pi \left(x\right)+7\right)+\left(294f_{4}+21f_{3}\right)\cdot \rho^{2}\left(\pi \left(x\right)+7\right)+\left(1372f_{4}+147f_{3}+14f_{2}\right)\cdot \rho \left(\pi \left(x\right)+7\right)=\;$$

(60) $$\begin{array}{c} =14x\cdot \left(2x^{2}+21x+98\right)\cdot f_{4}+21x\cdot \left(x+7\right)\cdot f_{3}+14x\cdot f_{2}\;\left(\mathrm{mod}\;L\right). \end{array}$$

For $L=16\cdot k_{L}\cdot \Psi$, $f_{4}=\Psi$, $f_{3}=k_{3,f}\cdot 2\Psi$, $k_{3,f}\in \left\{0,1,2,3\right\}$, $f_{2}=\left(2k_{2,f}\cdot k_{L}-1\right)\cdot \Psi$, $k_{2,f}\in \left\{1,2,3,4\right\}$, $k_{L}\in \left\{1,3\right\}$, Equation (60) becomes

$$14\cdot 2\Psi \cdot \rho^{3}\left(\pi \left(x\right)+7\right)+2\Psi \cdot \left(147+21k_{3,f}\right)\cdot \rho^{2}\left(\pi \left(x\right)+7\right)+$$

$$+2\Psi \cdot \left(686+147k_{3,f}+7\cdot \left(2k_{2,f}\cdot k_{L}-1\right)\right)\cdot \rho \left(\pi \left(x\right)+7\right)=\;$$

$$=7x\cdot \left(2x^{2}+21x+98\right)\cdot 2\Psi +21x\cdot \left(x+7\right)\cdot k_{3,f}\cdot 2\Psi +$$

(61) $$\begin{array}{c} +7x\cdot \left(2k_{2,f}\cdot k_{L}-1\right)\cdot 2\Psi \;\left(\mathrm{mod}\;16\cdot k_{L}\cdot \Psi \right). \end{array}$$

Equation (61) is fulfilled if and only if

$$14\cdot \rho^{3}\left(\pi \left(x\right)+7\right)+\left(147+21k_{3,f}\right)\cdot \rho^{2}\left(\pi \left(x\right)+7\right)+$$

$$+\left(686+147k_{3,f}+7\cdot \left(2k_{2,f}\cdot k_{L}-1\right)\right)\cdot \rho \left(\pi \left(x\right)+7\right)=\;$$

(62) $$\begin{array}{c} =7x\cdot \left(2x^{2}+21x+98\right)+21x\cdot \left(x+7\right)\cdot k_{3,f}+7x\cdot \left(2k_{2,f}\cdot k_{L}-1\right)\;\left(\mathrm{mod}\;8\cdot k_{L}\right), \end{array}$$

where

$$\rho \left(\pi \left(x\right)+7\right)\;\left(\mathrm{mod}\;8\cdot k_{L}\right)=x+\rho \left(7\right)+7\cdot \pi \left(x\right)\cdot (2\rho_{4}\cdot \left(2\pi^{2}\left(x\right)+2\cdot 7^{2}+3\cdot 7\cdot \pi \left(x\right)\right)+$$

$$+3\rho_{3}\cdot \left(\pi \left(x\right)+7\right)+2\rho_{2})\;\left(\mathrm{mod}\;8\cdot k_{L}\right)=x+\rho \left(7\right)+28\rho_{4}\cdot \pi^{3}\left(x\right)+$$

(63) $$\begin{array}{c} +21\cdot \left(14\rho_{4}+\rho_{3}\right)\cdot \pi^{2}\left(x\right)+7\cdot \left(196\rho_{4}+21\rho_{3}+2\rho_{2}\right)\cdot \pi \left(x\right)\;\left(\mathrm{mod}\;8\cdot k_{L}\right). \end{array}$$

For x=0, x=1, and x=3, Equation (62) becomes

$$14\cdot \rho^{3}\left(7\right)+\left(147+21k_{3,f}\right)\cdot \rho^{2}\left(7\right)+$$

(64) $$+\left(686+147k_{3,f}+7\cdot \left(2k_{2,f}\cdot k_{L}-1\right)\right)\cdot \rho \left(7\right)=0\;\left(\mathrm{mod}\;8\cdot k_{L}\right),$$

$$14\cdot \rho^{3}\left(\pi \left(1\right)+7\right)+\left(147+21k_{3,f}\right)\cdot \rho^{2}\left(\pi \left(1\right)+7\right)+$$

$$+\left(686+147k_{3,f}+7\cdot \left(2k_{2,f}\cdot k_{L}-1\right)\right)\cdot \rho \left(\pi \left(1\right)+7\right)=$$

(65) $$=847+168\cdot k_{3,f}+7\cdot \left(2k_{2,f}\cdot k_{L}-1\right)\;\left(\mathrm{mod}\;8\cdot k_{L}\right),$$

and

$$14\cdot \rho^{3}\left(\pi \left(3\right)+7\right)+\left(147+21k_{3,f}\right)\cdot \rho^{2}\left(\pi \left(3\right)+7\right)+$$

$$+\left(686+147k_{3,f}+7\cdot \left(2k_{2,f}\cdot k_{L}-1\right)\right)\cdot \rho \left(\pi \left(3\right)+7\right)=\;$$

(66) $$=3759+630\cdot k_{3,f}+21\cdot \left(2k_{2,f}\cdot k_{L}-1\right)\;\left(\mathrm{mod}\;8\cdot k_{L}\right),$$

respectively.

For $k_{L}=1$, $k_{2,f}^{\prime }=2k_{2,f}-1$ and $k_{2,\rho }^{\prime }=2k_{2,\rho }-1$, Equation (63) becomes

$$\rho \left(\pi \left(x\right)+7\right)\;\left(\mathrm{mod}\;8\right)=x+\rho \left(7\right)+4\rho_{4}\cdot \pi^{3}\left(x\right)+\left(6\rho_{4}+5\rho_{3}\right)\cdot \pi^{2}\left(x\right)+\left(4\rho_{4}+3\rho_{3}+6\rho_{2}\right)\cdot \pi \left(x\right)\;\left(\mathrm{mod}\;8\right)=$$

$$=x+7\rho_{1}+k_{\Psi }\cdot \left(6k_{3,\rho }+k_{2,\rho }^{\prime }+1\right)+4k_{\Psi }\cdot \pi^{3}\left(x\right)+$$

(67) $$+2k_{\Psi }\cdot \left(k_{3,\rho }+3\right)\cdot \pi^{2}\left(x\right)+2k_{\Psi }\cdot \left(3k_{3,\rho }+3k_{2,\rho }^{\prime }+2\right)\cdot \pi \left(x\right)\;\left(\mathrm{mod}\;8\right),$$

and Equations (64)–(66) become

(68) $$6\cdot \rho^{3}\left(7\right)+\left(5k_{3,f}+3\right)\cdot \rho^{2}\left(7\right)+\left(3k_{3,f}+7k_{2,f}^{\prime }+6\right)\cdot \rho \left(7\right)=0\;\left(\mathrm{mod}\;8\right),$$

where

(69) $$\rho \left(7\right)\;\left(\mathrm{mod}\;8\right)=7\rho_{1}+k_{\Psi }\cdot \left(6k_{3,\rho }+k_{2,\rho }^{\prime }+1\right)\;\left(\mathrm{mod}\;8\right),$$

$$6\cdot \rho^{3}\left(\pi \left(1\right)+7\right)+\left(5k_{3,f}+3\right)\cdot \rho^{2}\left(\pi \left(1\right)+7\right)+$$

(70) $$+\left(3k_{3,f}+7k_{2,f}^{\prime }+6\right)\cdot \rho \left(\pi \left(1\right)+7\right)+k_{2,f}^{\prime }+1=0\;\left(\mathrm{mod}\;8\right),$$

where

$$\rho \left(\pi \left(1\right)+7\right)\;\left(\mathrm{mod}\;8\right)=1+7\rho_{1}+k_{\Psi }\cdot \left(6k_{3,\rho }+k_{2,\rho }^{\prime }+1\right)+$$

$$+4k_{\Psi }\cdot {\left(f_{1}+k_{2,f}^{\prime }k_{\Psi }+k_{3,f}\cdot 2k_{\Psi }+k_{\Psi }\right)}^{3}+2k_{\Psi }\cdot \left(k_{3,\rho }+3\right)\cdot {\left(f_{1}+k_{2,f}^{\prime }k_{\Psi }+k_{3,f}\cdot 2k_{\Psi }+k_{\Psi }\right)}^{2}+$$

(71) $$+2k_{\Psi }\cdot \left(3k_{3,\rho }+3k_{2,\rho }^{\prime }+2\right)\cdot \left(f_{1}+k_{2,f}^{\prime }k_{\Psi }+k_{3,f}\cdot 2k_{\Psi }+k_{\Psi }\right)\;\left(\mathrm{mod}\;8\right),$$

and

$$6\cdot \rho^{3}\left(\pi \left(3\right)+7\right)+\left(5k_{3,f}+3\right)\cdot \rho^{2}\left(\pi \left(3\right)+7\right)+$$

(72) $$+\left(3k_{3,f}+7k_{2,f}^{\prime }+6\right)\cdot \rho \left(\pi \left(3\right)+7\right)+2k_{3,f}+3k_{2,f}^{\prime }+1=0\;\left(\mathrm{mod}\;8\right),$$

where

$$\rho \left(\pi \left(3\right)+7\right)\;\left(\mathrm{mod}\;8\right)=3+7\rho_{1}+k_{\Psi }\cdot \left(6k_{3,\rho }+k_{2,\rho }^{\prime }+1\right)+$$

$$+4k_{\Psi }\cdot {\left(3f_{1}+k_{2,f}^{\prime }k_{\Psi }+6k_{3,f}k_{\Psi }+k_{\Psi }\right)}^{3}+2k_{\Psi }\cdot \left(k_{3,\rho }+3\right)\cdot {\left(3f_{1}+k_{2,f}^{\prime }k_{\Psi }+6k_{3,f}k_{\Psi }+k_{\Psi }\right)}^{2}+$$

(73) $$+2k_{\Psi }\cdot \left(3k_{3,\rho }+3k_{2,\rho }^{\prime }+2\right)\cdot \left(3f_{1}+k_{2,f}^{\prime }k_{\Psi }+6k_{3,f}k_{\Psi }+k_{\Psi }\right)\;\left(\mathrm{mod}\;8\right),$$

respectively.

For $k_{L}=3$, Equation (63) becomes

$$\rho \left(\pi \left(x\right)+7\right)\;\left(\mathrm{mod}\;24\right)=x+\rho \left(7\right)+4\rho_{4}\cdot \pi^{3}\left(x\right)+3\cdot \left(2\rho_{4}+7\rho_{3}\right)\cdot \pi^{2}\left(x\right)+$$

$$+\left(4\rho_{4}+3\rho_{3}+14\rho_{2}\right)\cdot \pi \left(x\right)\;\left(\mathrm{mod}\;24\right)=$$

$$=x+7\rho_{1}+2k_{\Psi }\cdot \left(7k_{3,\rho }+3k_{2,\rho }\right)+4k_{\Psi }\cdot \pi^{3}\left(x\right)+6k_{\Psi }\cdot \left(7k_{3,\rho }+1\right)\cdot \pi^{2}\left(x\right)+$$

(74) $$+2k_{\Psi }\cdot \left(3k_{3,\rho }+6k_{2,\rho }+7\right)\cdot \pi \left(x\right)\;\left(\mathrm{mod}\;24\right)$$

and Equations (64)–(66) become

(75) $$\begin{array}{c} 14\cdot \rho^{3}\left(7\right)+3\cdot \left(7k_{3,f}+1\right)\cdot \rho^{2}\left(7\right)+\left(3k_{3,f}+18k_{2,f}+7\right)\cdot \rho \left(7\right)=0\;\left(\mathrm{mod}\;24\right), \end{array}$$

where

(76) $$\rho \left(7\right)\;\left(\mathrm{mod}\;24\right)=7\rho_{1}+2k_{\Psi }\cdot \left(7k_{3,\rho }+3k_{2,\rho }\right)\;\left(\mathrm{mod}\;24\right),$$

$$14\cdot \rho^{3}\left(\pi \left(1\right)+7\right)+3\cdot \left(7k_{3,f}+1\right)\cdot \rho^{2}\left(\pi \left(1\right)+7\right)+$$

(77) $$+\left(3k_{3,f}+18k_{2}+7\right)\cdot \rho \left(\pi \left(1\right)+7\right)+6k_{2,f}=0\;\left(\mathrm{mod}\;24\right),$$

where

$$\rho \left(\pi \left(1\right)+7\right)\;\left(\mathrm{mod}\;24\right)=1+7\rho_{1}+2k_{\Psi }\cdot \left(7k_{3,\rho }+3k_{2,\rho }\right)+$$

$$+4k_{\Psi }\cdot {\left(f_{1}+6k_{2,f}k_{\Psi }+2k_{3,f}k_{\Psi }\right)}^{3}+6k_{\Psi }\cdot \left(7k_{3,\rho }+1\right)\cdot {\left(f_{1}+6k_{2,f}k_{\Psi }+2k_{3,f}k_{\Psi }\right)}^{2}+$$

(78) $$+2k_{\Psi }\cdot \left(3k_{3,\rho }+6k_{2,\rho }+7\right)\cdot \left(f_{1}+6k_{2,f}k_{\Psi }+2k_{3,f}k_{\Psi }\right)\;\left(\mathrm{mod}\;24\right),$$

and

$$14\cdot \rho^{3}\left(\pi \left(3\right)+7\right)+3\cdot \left(7k_{3,f}+1\right)\cdot \rho^{2}\left(\pi \left(3\right)+7\right)+$$

(79) $$+\left(3k_{3,f}+18k_{2}+7\right)\cdot \rho \left(\pi \left(3\right)+7\right)+6\cdot \left(3k_{3,f}+3k_{2,f}+1\right)=0\;\left(\mathrm{mod}\;24\right),$$

where

$$\rho \left(\pi \left(3\right)+7\right)\;\left(\mathrm{mod}\;24\right)=3+7\rho_{1}+2k_{\Psi }\cdot \left(7k_{3,\rho }+3k_{2,\rho }\right)+$$

$$+4k_{\Psi }\cdot {\left(3f_{1}+6k_{2,f}k_{\Psi }+6k_{3,f}k_{\Psi }\right)}^{3}+6k_{\Psi }\cdot \left(7k_{3,\rho }+1\right)\cdot {\left(3f_{1}+6k_{2,f}k_{\Psi }+6k_{3,f}k_{\Psi }\right)}^{2}+$$

(80) $$+2k_{\Psi }\cdot \left(3k_{3,\rho }+6k_{2,\rho }+7\right)\cdot \left(3f_{1}+6k_{2,f}k_{\Psi }+6k_{3,f}k_{\Psi }\right)\;\left(\mathrm{mod}\;24\right),$$

respectively.

The four elements of permutation π(·) indicated in Figure 3 are written in detail below

(81) $$\begin{array}{c} \left\{\begin{array}{c} x_{2}\to \pi \left(x_{2}\right)\\ x_{2}+7\to \pi \left(x_{2}+7\right)\;\left(\mathrm{mod}\;L\right)\\ x_{1}\to \pi \left(x_{1}\right)=\pi \left(x_{2}\right)+7\;\left(\mathrm{mod}\;L\right)\\ x_{1}+7\to \pi \left(x_{1}+7\right)=\pi \left(x_{2}+7\right)+7\;\left(\mathrm{mod}\;L\right). \end{array}\right. \end{array}$$

Writing $x_{2}=\rho \left(\pi \left(x_{2}\right)\right)=\rho \left(\pi \left(x_{1}\right)-7\right)$ in the fourth equation from (81), with $x_{1}=x$, we have

(82) π(ρ(π(x)−7)+7)=π(x+7)−7(modL).

For $L=16\cdot k_{L}\cdot \Psi$, $f_{4}=\Psi$, $f_{3}=k_{3,f}\cdot 2\Psi$, $k_{3,f}\in \left\{0,1,2,3\right\}$, $f_{2}=\left(2k_{2,f}\cdot k_{L}-1\right)\cdot \Psi$, $k_{2,f}\in \left\{1,2,3,4\right\}$, $k_{L}\in \left\{1,3\right\}$, Equation (82) is fulfilled if and only if

$$14\cdot \rho^{3}\left(\pi \left(x\right)-7\right)+\left(147+21k_{3,f}\right)\cdot \rho^{2}\left(\pi \left(x\right)-7\right)+$$

$$+\left(686+147k_{3,f}+7\cdot \left(2k_{2,f}\cdot k_{L}-1\right)\right)\cdot \rho \left(\pi \left(x\right)-7\right)=$$

(83) $$=7x\cdot \left(2x^{2}+21x+98\right)+21x\cdot \left(x+7\right)\cdot k_{3,f}+7x\cdot \left(2k_{2,f}\cdot k_{L}-1\right)\;\left(\mathrm{mod}\;8\cdot k_{L}\right),$$

where

$$\rho \left(\pi \left(x\right)-7\right)\;\left(\mathrm{mod}\;8\cdot k_{L}\right)=x+\rho \left(-7\right)-7\cdot \pi \left(x\right)\cdot (2\rho_{4}\cdot \left(2\pi^{2}\left(x\right)+2\cdot 7^{2}-3\cdot 7\cdot \pi \left(x\right)\right)+$$

$$+3\rho_{3}\cdot \left(\pi \left(x\right)-7\right)+2\rho_{2})\;\left(\mathrm{mod}\;8\cdot k_{L}\right)=x+\rho \left(-7\right)-28\rho_{4}\cdot \pi^{3}\left(x\right)+21\cdot \left(14\rho_{4}-\rho_{3}\right)\cdot \pi^{2}\left(x\right)+$$

(84) $$-7\cdot \left(196\rho_{4}-21\rho_{3}+2\rho_{2}\right)\cdot \pi \left(x\right)\;\left(\mathrm{mod}\;8\cdot k_{L}\right).$$

For x=0, x=1, and x=3, Equation (84) becomes

$$14\cdot \rho^{3}\left(-7\right)+\left(147+21k_{3,f}\right)\cdot \rho^{2}\left(-7\right)+$$

(85) $$+\left(686+147k_{3,f}+7\cdot \left(2k_{2,f}\cdot k_{L}-1\right)\right)\cdot \rho \left(-7\right)=0\;\left(\mathrm{mod}\;8\cdot k_{L}\right),$$

$$14\cdot \rho^{3}\left(\pi \left(1\right)-7\right)+\left(147+21k_{3,f}\right)\cdot \rho^{2}\left(\pi \left(1\right)-7\right)+$$

$$+\left(686+147k_{3,f}+7\cdot \left(2k_{2,f}\cdot k_{L}-1\right)\right)\cdot \rho \left(\pi \left(1\right)-7\right)=$$

(86) $$=847+168\cdot k_{3,f}+7\cdot \left(2k_{2,f}\cdot k_{L}-1\right)\;\left(\mathrm{mod}\;8\cdot k_{L}\right),$$

and

$$14\cdot \rho^{3}\left(\pi \left(3\right)-7\right)+\left(147+21k_{3,f}\right)\cdot \rho^{2}\left(\pi \left(3\right)-7\right)+$$

$$+\left(686+147k_{3,f}+7\cdot \left(2k_{2,f}\cdot k_{L}-1\right)\right)\cdot \rho \left(\pi \left(3\right)-7\right)=$$

(87) $$=3759+630\cdot k_{3,f}+21\cdot \left(2k_{2,f}\cdot k_{L}-1\right)\;\left(\mathrm{mod}\;8\cdot k_{L}\right),$$

respectively.

For $k_{L}=1$, $k_{2,f}^{\prime }=2k_{2,f}-1$, and $k_{2,\rho }^{\prime }=2k_{2,\rho }-1$, Equation (84) becomes

$$\rho \left(\pi \left(x\right)-7\right)\;\left(\mathrm{mod}\;8\right)=x+\rho \left(1\right)+4\rho_{4}\cdot \pi^{3}\left(x\right)+3\cdot \left(2\rho_{4}+\rho_{3}\right)\cdot \pi^{2}\left(x\right)+$$

$$+\left(4\rho_{4}+3\rho_{3}+2\rho_{2}\right)\cdot \pi \left(x\right)\;\left(\mathrm{mod}\;8\right)=$$

$$=x+\rho_{1}+k_{\Psi }\cdot \left(2k_{3,\rho }+k_{2,\rho }^{\prime }+1\right)+4k_{\Psi }\cdot \pi^{3}\left(x\right)+6k_{\Psi }\cdot \left(k_{3,\rho }+1\right)\cdot \pi^{2}\left(x\right)+$$

(88) $$+2k_{\Psi }\cdot \left(3k_{3,\rho }+k_{2,\rho }^{\prime }+2\right)\cdot \pi \left(x\right)\;\left(\mathrm{mod}\;8\right),$$

and Equations (85)–(87) become

(89) $$6\cdot \rho^{3}\left(1\right)+\left(5k_{3,f}+3\right)\cdot \rho^{2}\left(1\right)+\left(3k_{3,f}+7\cdot k_{2,f}^{\prime }+6\right)\cdot \rho \left(1\right)=0\;\left(\mathrm{mod}\;8\right),$$

where

(90) $$\rho \left(1\right)\;\left(\mathrm{mod}\;8\right)=\rho_{1}+k_{\Psi }\cdot \left(2k_{3,\rho }+k_{2,\rho }+1\right)\;\left(\mathrm{mod}\;8\right),$$

$$6\cdot \rho^{3}\left(\pi \left(1\right)-7\right)+\left(5k_{3,f}+3\right)\cdot \rho^{2}\left(\pi \left(1\right)-7\right)+$$

(91) $$+\left(3k_{3,f}+7\cdot k_{2,f}^{\prime }+6\right)\cdot \rho \left(\pi \left(1\right)-7\right)+k_{2,f}^{\prime }+1=0\;\left(\mathrm{mod}\;8\right),$$

where

$$\rho \left(\pi \left(1\right)-7\right)\;\left(\mathrm{mod}\;8\right)=1+\rho_{1}+k_{2,\rho }k_{\Psi }+k_{3,\rho }\cdot 2k_{\Psi }+k_{\Psi }+4k_{\Psi }\cdot {\left(f_{1}+k_{2,f}^{\prime }k_{\Psi }+k_{3,f}\cdot 2k_{\Psi }+k_{\Psi }\right)}^{3}+$$

$$+6k_{\Psi }\cdot \left(k_{3,\rho }+1\right)\cdot {\left(f_{1}+k_{2,f}^{\prime }k_{\Psi }+k_{3,f}\cdot 2k_{\Psi }+k_{\Psi }\right)}^{2}+$$

(92) $$+2k_{\Psi }\cdot \left(3k_{3,\rho }+k_{2,\rho }+2\right)\cdot \left(f_{1}+k_{2,f}^{\prime }k_{\Psi }+k_{3,f}\cdot 2k_{\Psi }+k_{\Psi }\right)\;\left(\mathrm{mod}\;8\right),$$

and

$$6\cdot \rho^{3}\left(\pi \left(3\right)-7\right)+\left(5k_{3,f}+3\right)\cdot \rho^{2}\left(\pi \left(3\right)-7\right)+\left(3k_{3,f}+7\cdot k_{2,f}^{\prime }+6\right)\cdot \rho \left(\pi \left(3\right)-7\right)+$$

(93) $$+2k_{3,f}+3k_{2,f}^{\prime }+1=0\;\left(\mathrm{mod}\;8\right),$$

where

$$\rho \left(\pi \left(3\right)-7\right)\;\left(\mathrm{mod}\;8\right)=3+\rho_{1}+k_{\Psi }\cdot \left(2k_{3,\rho }+k_{2,\rho }+1\right)+4k_{\Psi }\cdot {\left(f_{1}+3k_{2,f}^{\prime }k_{\Psi }+k_{3,f}\cdot 2k_{\Psi }+3k_{\Psi }\right)}^{3}+$$

$$+6k_{\Psi }\cdot \left(k_{3,\rho }+1\right)\cdot {\left(f_{1}+3k_{2,f}^{\prime }k_{\Psi }+k_{3,f}\cdot 2k_{\Psi }+3k_{\Psi }\right)}^{2}+$$

(94) $$+2k_{\Psi }\cdot \left(3k_{3,\rho }+k_{2,\rho }+2\right)\cdot \left(f_{1}+3k_{2,f}^{\prime }k_{\Psi }+k_{3,f}\cdot 2k_{\Psi }+3k_{\Psi }\right)\;\left(\mathrm{mod}\;8\right),$$

respectively.

For $k_{L}=3$, Equation (84) becomes

$$\rho \left(\pi \left(x\right)-7\right)\;\left(\mathrm{mod}\;24\right)=x+\rho \left(-7\right)+20\rho_{4}\cdot \pi^{3}\left(x\right)+3\cdot \left(2\rho_{4}+\rho_{3}\right)\cdot \pi^{2}\left(x\right)+$$

$$+\left(20\rho_{4}+3\rho_{3}+10\rho_{2}\right)\cdot \pi \left(x\right)\;\left(\mathrm{mod}\;24\right)=x+17\cdot \left(\rho_{1}+6k_{2,\rho }k_{\Psi }+10k_{3,\rho }k_{\Psi }\right)+$$

(95) $$+20k_{\Psi }\cdot \pi^{3}\left(x\right)+6k_{\Psi }\cdot \left(k_{3,\rho }+1\right)\cdot \pi^{2}\left(x\right)+2k_{\Psi }\cdot \left(3k_{3,\rho }+6k_{2,\rho }+5\right)\cdot \pi \left(x\right)\;\left(\mathrm{mod}\;24\right),$$

and Equations (85)–(87) become

$$14\cdot \rho^{3}\left(-7\right)+3\cdot \left(7k_{3,f}+1\right)\cdot \rho^{2}\left(-7\right)+$$

(96) $$+\left(3k_{3,f}+18k_{2,f}+7\right)\cdot \rho \left(-7\right)=0\;\left(\mathrm{mod}\;24\right),$$

where

(97) $$\rho \left(-7\right)\;\left(\mathrm{mod}\;24\right)=17\cdot \left(\rho_{1}+6k_{2,\rho }k_{\Psi }+10k_{3,\rho }k_{\Psi }\right)\;\left(\mathrm{mod}\;24\right),$$

$$14\cdot \rho^{3}\left(\pi \left(1\right)-7\right)+3\cdot \left(7k_{3,f}+1\right)\cdot \rho^{2}\left(\pi \left(1\right)-7\right)+$$

(98) $$+\left(3k_{3,f}+18k_{2,f}+7\right)\cdot \rho \left(\pi \left(1\right)-7\right)+6k_{2,f}=0\;\left(\mathrm{mod}\;24\right),$$

where

$$\rho \left(\pi \left(1\right)-7\right)\;\left(\mathrm{mod}\;24\right)=1+17\cdot \left(\rho_{1}+6k_{2,\rho }k_{\Psi }+10k_{3,\rho }k_{\Psi }\right)+$$

$$+20k_{\Psi }\cdot {\left(f_{1}+6k_{2,f}k_{\Psi }+2k_{3,f}k_{\Psi }\right)}^{3}+6k_{\Psi }\cdot \left(k_{3,\rho }+1\right)\cdot {\left(f_{1}+6k_{2,f}k_{\Psi }+2k_{3,f}k_{\Psi }\right)}^{2}+$$

(99) $$+2k_{\Psi }\cdot \left(3k_{3,\rho }+6k_{2,\rho }+5\right)\cdot \left(f_{1}+6k_{2,f}k_{\Psi }+2k_{3,f}k_{\Psi }\right)\;\left(\mathrm{mod}\;24\right),$$

and

$$14\cdot \rho^{3}\left(\pi \left(3\right)-7\right)+3\cdot \left(7k_{3,f}+1\right)\cdot \rho^{2}\left(\pi \left(3\right)-7\right)+$$

(100) $$+\left(3k_{3,f}+18k_{2,f}+7\right)\cdot \rho \left(\pi \left(3\right)-7\right)+6\cdot \left(3k_{3,f}+3k_{2,f}+1\right)=0\;\left(\mathrm{mod}\;24\right),$$

where

$$\rho \left(\pi \left(3\right)-7\right)\;\left(\mathrm{mod}\;24\right)=3+17\cdot \left(\rho_{1}+6k_{2,\rho }k_{\Psi }+10k_{3,\rho }k_{\Psi }\right)+$$

$$+12k_{\Psi }\cdot {\left(f_{1}+2k_{2,f}k_{\Psi }+2k_{3,f}k_{\Psi }\right)}^{3}+6k_{\Psi }\cdot \left(k_{3,\rho }+1\right)\cdot {\left(f_{1}+2k_{2,f}k_{\Psi }+2k_{3,f}k_{\Psi }\right)}^{2}+$$

(101) $$+6k_{\Psi }\cdot \left(3k_{3,\rho }+6k_{2,\rho }+5\right)\cdot \left(f_{1}+2k_{2,f}k_{\Psi }+2k_{3,f}k_{\Psi }\right)\;\left(\mathrm{mod}\;24\right),$$

respectively.

Solutions of Equations (68), (70), (72), (89), (91), and (93) for variables $k_{3,f}$, $k_{2,f}^{\prime }$, and $f_{1}\;\left(\mathrm{mod}\;8\right)$, which fulfill the results from Lemma 2, are given in Table 9 and Table 10. It can be observed that they can be summarized as in Table 11.

Solutions of equations (75), (77), (79), (96), (98), and (100) in variables $k_{3,f}$, $k_{2,f}$, $f_{1}\;\left(\mathrm{mod}\;48\right)$, which fulfill the results from Lemma 2, are given in Table 12 and Table 13. It can be observed that they can be summarized as in Table 14.

From Table 11 and Table 14, it results that an interleaver pattern as in Figure 2 or Figure 3 always appears for $x_{1}=0$ or $x_{1}=1$ or $x_{1}=3$, when $k_{3,f}\in \left\{0,2\right\}$ and $k_{2,f}\in \left\{1,2,3,4\right\}$ or when $k_{3,f}\in \left\{1,3\right\}$ and $k_{2,f}\in \left\{2,4\right\}$. For an interleaver pattern as in Figure 2 or Figure 3, the weight of the codeword for classical nominal 1/3 rate turbo codes with two RSC codes having generator matrix G=[1,15/13], is equal to 4+4·6=28, because each of the four error patterns with weight of 2 lead to parity weight of 6. Because an interleaver pattern as in Figure 2 or Figure 3 always appears in the previous conditions, it results that the minimum distance is upper bounded by the value of 28. □

Combining the results from Theorems 1 and 2, it results that the upper bound of 36 is reached only for $k_{3,f}\in \left\{1,3\right\}$ and $k_{2,f}\in \left\{1,3\right\}$, $\forall k_{L}\in \left\{1,3\right\}$. Thus, the task for finding good 4-PPs is facilitated with this result, because coefficients $f_{4}$, $f_{3}$, and $f_{2}$ have only four possible combinations.

We note that from the LTE interleaver lengths [13], there exist 25 lengths of the form (5); namely 48, 80, 112, 176, 208, 240, 272, 304, 336, 368, 464, 496, 528, 560, 592, 624, 656, 688, 752, 816, 848, 880, 912, 944, and 976. From these, for the lengths 40, 208, 304, 496, 592, 624, 688, 912, and 976, restriction conditions (6) on coefficients are not required, and thus, the result in the paper is fully general. Examples of 4-PP interleavers that reach the upper bound of 36 are those from [17] for the interleaver lengths 368, 464, and 496, when dual trellis termination [31] is used.

## 4. Remarks and Examples

### 4.1. Remarks

In this subsection, we make some remarks regarding the upper bounds on the minimum distance derived in [19] and those on the minimum distance derived in this paper. From Lemma 3.2 and Table 2 in [19], it results that an upper bound on minimum distance for turbo codes with any degree PP interleavers is equal to 36 in the following conditions:

(1) The PPs can be represented by a parallel linear PP (PLPP) with the minimum number of linear PPs (LPPs) from the PLPP representation equal to two or 14.
(2) The coefficients of the first degree term of LPPs from the PLPP representation are all equal to each other. We denote by $D_{eq}$ the minimum number of LPPs from the PLPP representation fulfilling this condition.

In the following, we prove that 4-PPs fulfilling the conditions from Theorem 1 can be represented by PLPPs with the value of $D_{eq}$ greater than two. For this task, it is enough to prove that these 4-PPs do not allow a PLPP representation with $D_{eq}=2$ LPPs. A 4-PP allows a PLPP representation with $D_{eq}$ component LPPs if and only if the following condition is fulfilled

$$f\left(D_{eq}\cdot y+D_{eq}+i\right)-f\left(D_{eq}\cdot y+i\right)=f\left(D_{eq}+i\right)-f\left(i\right)\;\left(\mathrm{mod}\;L\right),$$

(102) $$\forall i\in \left\{0,1,\ldots ,D_{eq}-1\right\},\forall y\in \left\{1,2,\ldots ,L/D_{eq}-2\right\}.$$

With f(x) from (1) fulfilling conditions (6) when $3∤(p_{i}-1)$ and with *L* as in (5), Equation (102) is equivalent to

$$\Psi \cdot \left(2D_{eq}^{4}y\cdot \left(2y^{2}+3y+2\right)+12D_{eq}^{3}iy\cdot \left(y+1\right)+12D_{eq}^{2}i^{2}y\right)+2k_{3,f}\Psi \cdot \left(3D_{eq}^{3}y\cdot \left(y+1\right)+6D_{eq}^{2}iy\right)+$$

(103) $$+k_{2,f}^{\prime }\Psi \cdot D_{eq}^{2}\cdot 2y=0\;\left(\mathrm{mod}\;16k_{L}\Psi \right),\forall i\in \left\{0,1,\ldots ,D_{eq}-1\right\},\forall y\in \left\{1,2,\ldots ,16k_{L}\Psi /D_{eq}-2\right\},$$

or

$$D_{eq}^{4}y\cdot \left(2y^{2}+3y+2\right)+6D_{eq}^{3}iy\cdot \left(y+1\right)+6D_{eq}^{2}i^{2}y+k_{3,f}\cdot 3D_{eq}^{2}y\cdot \left(D_{eq}\cdot \left(y+1\right)+2i\right)+k_{2,f}^{\prime }D_{eq}^{2}\cdot y=$$

(104) $$=0\;\left(\mathrm{mod}\;8k_{L}\right),\forall i\in \left\{0,1,\ldots ,D_{eq}-1\right\},\forall y\in \left\{1,2,\ldots ,8k_{L}-1\right\}.$$

Because $k_{L}\in \left\{1,3\right\}$, we can write $3=k_{L}\cdot \left(k_{L}-1\right)$. Thus, Equation (104) is equivalent to

$$D_{eq}^{4}y\cdot \left(2y^{2}+3y+2\right)+2\cdot k_{L}\cdot \left(k_{L}-1\right)\cdot D_{eq}^{3}iy\cdot \left(y+1\right)+2\cdot k_{L}\cdot \left(k_{L}-1\right)\cdot D_{eq}^{2}i^{2}y+$$

$$+k_{3,f}\cdot k_{L}\cdot \left(k_{L}-1\right)\cdot D_{eq}^{2}y\cdot \left(D_{eq}\cdot \left(y+1\right)+2i\right)+k_{2,f}^{\prime }D_{eq}^{2}\cdot y=0\;\left(\mathrm{mod}\;8k_{L}\right),$$

(105) $$\forall i\in \left\{0,1,\ldots ,D_{eq}-1\right\},\forall y\in \left\{1,2,\ldots ,8k_{L}-1\right\}.$$

For $D_{eq}=2$, Equation (105) is equivalent to

(106) $$8y^{3}+4\cdot \left(k_{2,f}^{\prime }+2\right)\cdot y=0\;\left(\mathrm{mod}\;8k_{L}\right),\forall y\in \left\{1,2,\ldots ,8k_{L}-1\right\},$$

or

(107) $$2y^{3}+\left(k_{2,f}^{\prime }+2\right)\cdot y=0\;\left(\mathrm{mod}\;2k_{L}\right),\forall y\in \left\{1,\ldots ,2k_{L}-1\right\}.$$

For the coefficients of 4-PPs given in Theorem 1 we have $k_{2,f}^{\prime }\;\left(\mathrm{mod}\;2\right)=1$ when $k_{L}=1$ and $k_{2,f}^{\prime }\;\left(\mathrm{mod}\;6\right)=5$ when $k_{L}=3$. Thus (107) is equivalent to

(108) y=0(mod2),fory=1,

when $k_{L}=1$, and to

(109) $$2y^{3}+y=0\;\left(\mathrm{mod}\;6\right),\forall y\in \left\{1,2,\ldots ,5\right\},$$

when $k_{L}=3$.

It is clear that equalities (108) and (109) are not fulfilled for y=1. Therefore, it results that the 4-PPs given in Theorem 1 do not allow a PLPP representation with $D_{eq}=2$ component LPPs.

We can have $D_{eq}=3$ only when $k_{L}=3$, because 3∤L for $k_{L}=1$. For $D_{eq}=3$ and $k_{L}=3$, Equation (105) is equivalent to

$$18y^{3}+3y^{2}\cdot \left(1+6i+2i^{2}+3k_{3,f}\right)+3y\cdot \left(6+6i+3k_{3,f}+2ik_{3,f}+3k_{2,f}^{\prime }\right)=0\;\left(\mathrm{mod}\;24\right),$$

(110) ∀i∈{0,1,2},∀y∈{1,2,…,23},

or

$$6y^{3}+y^{2}\cdot \left(1+6i+2i^{2}+3k_{3,f}\right)+y\cdot \left(6+6i+3k_{3,f}+2ik_{3,f}+3k_{2,f}^{\prime }\right)=0\;\left(\mathrm{mod}\;8\right),$$

(111) ∀i∈{0,1,2},∀y∈{1,2,…,7}.

Because there is no cubic null polynomial modulo 8 with the coefficient of the third term degree equal to six, it results that the 4-PPs from Theorem 1 can not be represented by a PLPP with three component LPPs.

For $D_{eq}=4$, Equation (105) is equivalent to

(112) $$8y^{3}+8\cdot \left(2k_{2,f}^{\prime }+1\right)\cdot y=0\;\left(\mathrm{mod}\;8k_{L}\right),\forall y\in \left\{1,2,\ldots ,8k_{L}-1\right\},$$

or

(113) $$y^{3}+\left(2k_{2,f}^{\prime }+1\right)\cdot y=0\;\left(\mathrm{mod}\;k_{L}\right),\forall y\in \left\{1,\ldots ,k_{L}-1\right\}.$$

For $k_{L}=1$, Equation (113) is, obviously, fulfilled. For $k_{L}=3$, because $k_{2,f}^{\prime }\;\left(\mathrm{mod}\;3\right)=2$, Equation (113) becomes

(114) $$y^{3}+2y=0\;\left(\mathrm{mod}\;3\right),\forall y\in \left\{1,2\right\}.$$

It can be easily verified that the equality from (114) is fulfilled for y∈{1,2}.

To show that the 4-PPs established in Theorem 1 can be represented by a PLPP with $D_{eq}=4$ LPPs, we still have to prove that all the coefficients of the first term degree of the four LPPs are equal to each other. For that, we have to show that

(115) f(y+4)−f(y)=f(4)−f(0)(modL),∀y∈{1,2,3}.

Equation (115) is equivalent to

(116) $$f_{4}\cdot \left(4\cdot y^{3}\cdot 4+6\cdot y^{2}\cdot 4^{2}+4\cdot y\cdot 4^{3}\right)+f_{3}\cdot \left(3\cdot y^{2}\cdot 4+3\cdot y\cdot 4^{2}\right)+f_{2}\cdot 2\cdot y\cdot 4=0\;\left(\mathrm{mod}\;L\right),\forall y\in \left\{1,2,3\right\};$$

or, with $f_{4}=\Psi$, $f_{3}=k_{3,f}\cdot 2\Psi$, $f_{2}=k_{2,f}^{\prime }\cdot \Psi$, and $L=16k_{L}\Psi$,

(117) $$2y^{3}+3k_{3,f}y^{2}+\left(k_{2,f}^{\prime }+2\right)\cdot y=0\;\left(\mathrm{mod}\;2k_{L}\right),\forall y\in \left\{1,2,3\right\}.$$

For the 4-PPs established in Theorem 1, we have $3k_{3,f}\;\left(\mathrm{mod}\;2k_{L}\right)=k_{L}$ and $\left(k_{2,f}^{\prime }+2\right)\;\left(\mathrm{mod}\;2k_{L}\right)=1$, $\forall k_{L}\in \left\{1,3\right\}$. Then, for $k_{L}=1$ and $k_{L}=3$, Equation (117) becomes

(118) $$y^{2}+y=0\;\left(\mathrm{mod}\;2\right),\forall y\in \left\{1,2,3\right\}$$

and

(119) $$2y^{3}+3y^{2}+y=0\;\left(\mathrm{mod}\;6\right),\forall y\in \left\{1,2,3\right\},$$

respectively. It can be easily verified that the equalities from (118) and (119) are fulfilled for y∈{1,2,3}. Thus, the 4-PPs established in Theorem 1 always allow a PLPP representation with $D_{eq}=4$ LPPs. Therefore, from Table 2 in [19] it results that the tightest upper bound derived in [19] is equal to 52. Thus, the upper bound of 36, derived in Theorem 1, is much tighter. The examples of 4-PPs given in the next subsection show that this upper bound can be reached.

### 4.2. Examples

Table 15 shows some CPPs and 4-PPs with optimum minimum distance for several LTE interleaver lengths of the form given in (5). We note that for all these 4-PPs we have $D_{eq}=4$, and thus, the best upper bound derived in [19] is equal to 52. Minimum distances ($d_{min}$) and corresponding multiplicities ($N_{d_{min}}$), spread factors (*D*), nonlinearity degrees (ζ), and refined nonlinearity degrees ($\zeta^{\prime }$) for each CPP and each 4-PP are also given in Table 15. As it can be observed, CPPs have optimum distances greater than those of 4-PPs (38 compared to 36) and the corresponding multiplicities for CPPs are equal to about a half of those for 4-PPs. These relation between the multiplicities for CPPs and 4-PPs with optimum distances is explained by means of nonlinearity degrees. In [21], it was proven that CPPs with optimum distance have the nonlinearity degree equal to $\zeta_{CPP,d_{min}-opt}=8$. In Appendix A, it is proven that the nonlinearity degree of 4-PPs for interleaver lengths of the form (5), fulfilling conditions (6) when $3∤(p_{i}-1)$, is equal to

(120) $$\zeta_{4-PP}=\left\{\begin{array}{c} 4\;\mathrm{when}\;k_{3,f}\in \left\{1,3\right\}\\ 8\;\mathrm{when}\;k_{3,f}\in \left\{0,2\right\}, \end{array}\right.$$

where the coefficient of the third term of 4-PP is $f_{3}=k_{3,f}\cdot 2\Psi$. Because 4-PPs with optimum distance have $k_{3,f}\in \left\{1,3\right\}$, it results that their nonlinearity degree is equal to $\zeta_{4-PP_{d_{min}-opt}}=4=\zeta_{CPP_{d_{min}-opt}}/2$. Thus, the result for the multiplicities is explained.

We also note that the good QPPs reported in Table XIII from [21] have the minimum distance equal to 38 and the corresponding multiplicities are approximately equal to those for 4-PPs from Table 15 in this paper. The results for multiplicities are explained by the fact that QPPs given in [21] have the nonlinearity degree $\zeta_{QPP_{d_{min}-opt}}=4=\zeta_{4-PP_{d_{min}-opt}}$.

Taking into account the above, it is expected that CPPs and QPPs for these interleaver lengths to lead to better error rate performances compared to 4-PPs.

An estimation of asymptotic improvement in terms of the error rate for CPP and QPP interleavers compared to 4-PP interleavers can be given if we compare the upper bounds on error rates for distance spectra of the turbo codes truncated at the first term. For an additive white Gaussian noise (AWGN) channel with the signal to noise ratio SNR, the frame error rate (FER) for a block code with coding rate $R_{c}$, minimum distance $d_{\min}$, and the corresponding multiplicity $N_{d_{\min}}$, is upper bounded by

(121) $$FER\leq TUB_{erfc}\left(FER\right)<TUB_{exp}\left(FER\right),$$

where

(122) $$TUB_{erfc}\left(FER\right)=0.5\cdot N_{d_{\min}}\cdot erfc\left(\sqrt{R_{c}\cdot d_{\min}\cdot SNR}\right)=N_{d_{\min}}\cdot \frac{1}{\sqrt{\pi }}\cdot \int_{\sqrt{R_{c}\cdot d_{\min}\cdot SNR}}^{+\infty }e^{-t^{2}}dt$$

and

(123) $$TUB_{exp}\left(FER\right)=0.5\cdot N_{d_{\min}}\cdot e^{-R_{c}\cdot d_{\min}\cdot SNR}.$$

From Table 15 it results that the multiplicity of the codewords of weight $d_{\min}$ is approximately equal to *L* for CPP interlevears and to 2L for 4-PP interleavers. From the QPPs reported in Table XIII from [21], it results that for QPPs, the best minimum distance is equal to 38 and the corresponding multiplicity is approximately equal to 2L. Thus, if we use the upper bounds with $TUB_{exp}\left(FER\right)$ from (123), the FER for QPP, CPP, and 4-PP interleavers, is approximately upper bounded by

(124) $$FER_{QPP}<0.5\cdot 2L\cdot e^{-R_{c}\cdot 38\cdot SNR},$$

(125) $$FER_{CPP}<0.5\cdot L\cdot e^{-R_{c}\cdot 38\cdot SNR},$$

and

(126) $$FER_{4-PP}<0.5\cdot 2L\cdot e^{-R_{c}\cdot 36\cdot SNR},$$

respectively.

From (124)–(126), it results that when considering the interleaver lengths of the form given in (5) and turbo codes of nominal 1/3 coding rate with RSC component codes with generator matrix G=[1,15/13], the asymptotic coding gain for QPPs compared to 4-PPs, is equal to

(127) $$G_{c_{QPP,4-PP}}\left(TUB_{exp}\left(FER\right)\right)=10\cdot \log_{10}\left(\frac{38}{36}\right)≅0.235\;\mathrm{dB}$$

and the asymptotic coding gain for CPPs compared to 4-PPs, for a given FER value, is equal to

(128) $$G_{c_{CPP,4-PP}}\left(TUB_{exp}\left(FER\right)\right)=10\cdot \log_{10}\left(\frac{38}{36}\right)-10\cdot \log_{10}\left(1+\frac{\log_{10}\left(2\right)}{\log_{10}\left(FER/L\right)}\right).$$

For example, for a target $FER=3\cdot 10^{-6}$ and for interleaver length L=656, the coding gain from (128) becomes $G_{c_{CPP,4-PP}}\left(TUB_{exp}\left(FER\right)\right)≅0.395$ dB. Increasing the interleaver length, $G_{c_{CPP,4-PP}}\left(TUB_{exp}\left(FER\right)\right)$ resulting from (128) decreases easily. For an increase of interleaver length with a factor of approximately 25 compared to 656, the coding gain from (128) decreases with about 0.023 dB.

**Figure 4:**
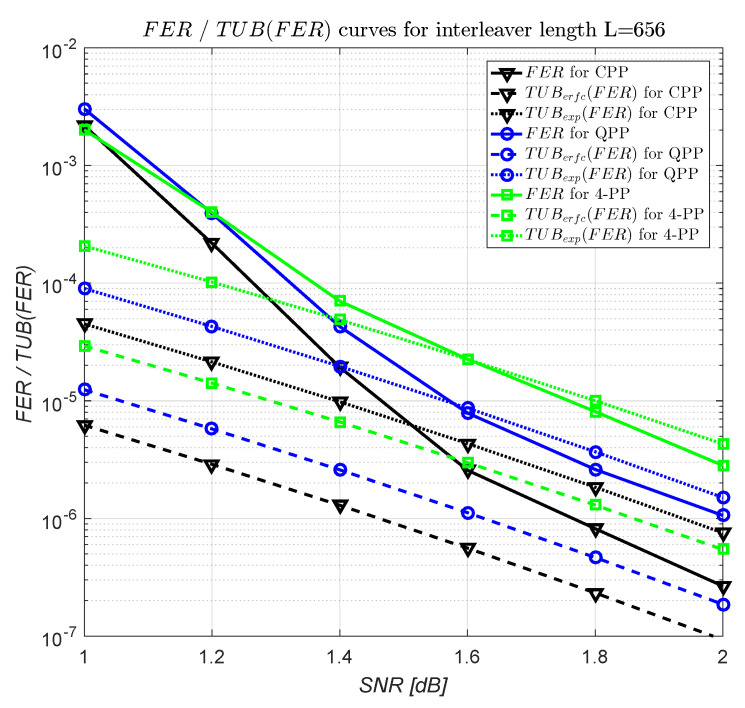
Frame error rate (FER) and truncated upper bound of FER (TUB(FER)) curves for interleaver length L = 656.

In Figure 4, the FER, $TUB_{erfc}\left(FER\right)$, and $TUB_{exp}\left(FER\right)$ curves for 4-PP, CPP, and QPP of interleaver length L = 656 are shown. The 4-PP and the CPP are those from Table 15, and the QPP is $246x^{2}+21x\;\left(\mathrm{mod}\;656\right)$ given in [21]. For FER curves, the Max-Log-MAP algorithm with a scaling coefficient of the extrinsec information of 0.75 was used. We note that the considered multiplicities for $TUB_{erfc}\left(FER\right)$ and $TUB_{exp}\left(FER\right)$ curves are the estimated ones; i.e., 2L, *L*, and 2L, for 4-PP, CPP, and QPP, respectively. For $FER=3\cdot 10^{-6}$, from Figure 4, it results that

(1) The coding gains resulting from FER curves are $G_{c_{CPP,4-PP}}\left(FER\right)=0.393$ dB and $G_{c_{QPP,4-PP}}\left(FER\right)=0.229$ dB and
(2) The coding gains resulting from $TUB_{erfc}\left(FER\right)$ curves are $G_{c_{CPP,4-PP}}\left(TUB_{erfc}\left(FER\right)\right)=0.409$ dB and $G_{c_{QPP,4-PP}}\left(TUB_{erfc}\left(FER\right)\right)=0.235$ dB.

We observe that these coding gains are very close to those previously estimated by the $TUB_{exp}\left(FER\right)$ upper bounds.

## 5. Conclusions

In this paper, we obtained the upper bounds of the minimum distance for turbo codes when using 4-PP interleavers. The component RSC codes were those from the LTE standard and 1/3 nominal coding rate. The interleaver lengths in question were of the form (5), and condition (6) was applied for 4-PP coefficients when for a prime $p_{i}$, $3∤(p_{i}-1)$. The two obtained upper bounds have the values of 28 and 36 for different classes of 4-PP coefficients. The result obtained in this paper has theoretical importance. The highest upper bound for 4-PPs (i.e., 36) is smaller than that for CPPs or QPPs (i.e., 38), while the corresponding multiplicities are about twice as high as those for CPPs and approximately equal to those for QPPs. Thus, it is expected that CPPs and QPPs for the interleaver lengths in question are better compared to 4-PPs. 

## Figures and Tables

**Table 1:** Conditions for coefficients $f_{1},f_{2},f_{3},$ and $f_{4}$ so that π(x) in (1) is a fourth degree permutation polynomial (4-PP) modulo *L* of the form (2) ($p_{i}$ is a prime number so that $p_{i}|L$).

(1)	$p_{i}=2$	$n_{L,2}>1$	$f_{1}\neq 0,\left(f_{2}+f_{4}\right)=0,f_{3}=0\;\left(\mathrm{mod}\;2\right)$
(2)	$p_{i}=3$	$n_{L,3}=1$	$\left(f_{1}+f_{3}\right)\neq 0,\left(f_{2}+f_{4}\right)=0\;\left(\mathrm{mod}\;3\right)$
(3)	$3|(p_{i}-1)$	$n_{L,p_{i}}=1$	$f_{1}\neq 0,f_{2}=0,f_{3}=0,f_{4}=0\;\left(\mathrm{mod}\;p_{i}\right)$
	($p_{i}>7$)		
(4)	$3∤(p_{i}-1)$	$n_{L,p_{i}}=1$	$f_{1}\neq 0,f_{2}=0,f_{3}=0,f_{4}=0\;\left(\mathrm{mod}\;p_{i}\right)$ or
			$f_{2}^{2}=3f_{1}f_{3}\;\left(\mathrm{mod}\;p_{i}\right)$, $f_{3}\neq 0,f_{4}=0\;\left(\mathrm{mod}\;p_{i}\right)$

**Table 2:** Possible values for coefficients $f_{4}$, $f_{3}$, and $f_{2}$ so that π(x) in (1) is a true 4-PP modulo L of the form (5).

*L*	$f_{4}$	$f_{3}$	$f_{2}$
16Ψ	Ψ	0 or 2Ψ or 4Ψ or 6Ψ	Ψ or 3Ψ or 5Ψ or 7Ψ
48Ψ	Ψ	0 or 2Ψ or 4Ψ or 6Ψ	5Ψ or 11Ψ or 17Ψ or 23Ψ

**Table 3:** Coefficients of an inverse 4-PP for a 4-PP (mod16Ψ) (Part I) ($k_{2,f}=\left(k_{2,f}^{\prime }+1\right)/2$ and $k_{2,\rho }=\left(k_{2,\rho }^{\prime }+1\right)/2$). For $f_{1}\;\left(\mathrm{mod}\;16\right)=f_{1,8}+8$, $\rho_{1}\;\left(\mathrm{mod}\;16\right)=\left(\rho_{1,f_{1,8}}+8\right)\;\left(\mathrm{mod}\;16\right)$.

$k_{3,f}$	$k_{2,f}^{\prime }$	$f_{1,8}$	$k_{3,\rho }$	$k_{2,\rho }^{\prime }$	$\rho_{1,f_{1,8}}$	*k* for	$\rho_{1,f_{1,8}}$	*k* for
					for $k_{\Psi ,4}=1$	$k_{\Psi ,4}=1$	for $k_{\Psi ,4}=3$	$k_{\Psi ,4}=3$
0	1	1	0	1	13	12	13	4
		3	2	5	3	8	11	0
		5	0	1	9	12	9	4
		7	2	5	15	8	7	0
	3	1	2	3	13	12	5	12
		3	0	3	11	0	11	0
		5	2	3	1	4	9	4
		7	0	3	15	8	15	8
	5	1	0	5	13	12	13	4
		3	2	1	3	8	11	0
		5	0	5	9	12	9	4
		7	2	1	15	8	7	0
	7	1	2	7	13	12	5	12
		3	0	7	3	8	3	8
		5	2	7	1	4	9	4
		7	0	7	7	0	7	0
1	1	1	1	5	5	4	13	4
		3	3	1	3	8	3	8
		5	1	5	1	4	9	4
		7	3	1	15	8	15	8
	3	1	3	3	5	4	13	4
		3	1	3	3	8	3	8
		5	3	3	9	12	1	12
		7	1	3	7	0	7	0
	5	1	1	1	5	4	13	4
		3	3	5	3	8	3	8
		5	1	1	1	4	9	4
		7	3	5	15	8	15	8
	7	1	3	7	13	12	5	12
		3	1	7	3	8	3	8
		5	3	7	1	4	9	4
		7	1	7	7	0	7	0

**Table 4:** Coefficients of an inverse 4-PP for a 4-PP (mod16Ψ) (Part II) ($k_{2,f}=\left(k_{2,f}^{\prime }+1\right)/2$ and $k_{2,\rho }=\left(k_{2,\rho }^{\prime }+1\right)/2$). For $f_{1}\;\left(\mathrm{mod}\;16\right)=f_{1,8}+8$, $\rho_{1}\;\left(\mathrm{mod}\;16\right)=\left(\rho_{1,f_{1,8}}+8\right)\;\left(\mathrm{mod}\;16\right)$.

$k_{3,f}$	$k_{2,f}^{\prime }$	$f_{1,8}$	$k_{3,\rho }$	$k_{2,\rho }^{\prime }$	$\rho_{1,f_{1,8}}$	*k* for	$\rho_{1,f_{1,8}}$	*k* for
					for $k_{\Psi ,4}=1$	$k_{\Psi ,4}=1$	for $k_{\Psi ,4}=3$	$k_{\Psi ,4}=3$
2	1	1	2	1	5	4	5	12
		3	0	5	3	8	11	0
		5	2	1	1	4	1	12
		7	0	5	15	8	7	0
	3	1	0	3	5	4	13	4
		3	2	3	11	0	11	0
		5	0	3	9	12	1	12
		7	2	3	15	8	15	8
	5	1	2	5	5	4	5	12
		3	0	1	3	8	11	0
		5	2	5	1	4	1	12
		7	0	1	15	8	7	0
	7	1	0	7	5	4	13	4
		3	2	7	3	8	3	8
		5	0	7	9	12	1	12
		7	2	7	7	0	7	0
3	1	1	3	5	13	12	5	12
		3	1	1	3	8	3	8
		5	3	5	9	12	1	12
		7	1	1	15	8	15	8
	3	1	1	3	13	12	5	12
		3	3	3	3	8	3	8
		5	1	3	1	4	9	4
		7	3	3	7	0	7	0
	5	1	3	1	13	12	5	12
		3	1	5	3	8	3	8
		5	3	1	9	12	1	12
		7	1	5	15	8	15	8
	7	1	1	7	5	4	13	4
		3	3	7	3	8	3	8
		5	1	7	9	12	1	12
		7	3	7	7	0	7	0

**Table 5:** Coefficients of an inverse 4-PP for a 4-PP (mod48Ψ) (Part I). For $f_{1}\;\left(\mathrm{mod}\;48\right)=f_{1,24}+24$, $\rho_{1}\;\left(\mathrm{mod}\;48\right)=\left(\rho_{1,f_{1,24}}+24\right)\;\left(\mathrm{mod}\;48\right)$.

$k_{3,f}$	$k_{2,f}$	$f_{1,24}$	$k_{3,\rho }$	$k_{2,\rho }$	$\rho_{1,f_{1,24}}$ for	*k* for	$\rho_{1,f_{1,24}}$ for	*k* for
					$k_{\Psi ,12}=1$	$k_{\Psi ,12}=1$	$k_{\Psi ,12}=7$	$k_{\Psi ,12}=7$
					($\rho_{1,1}$)	($k_{1}$)		
0	1	1/13	0	1	13/1	12/12	$\rho_{1,1}$	$\left(k_{1}+24\right)$
		5/17	0	1	41/29	12/12	(mod48)	(mod48)
		7/19	2	3	15/3	8/8	$\left(\rho_{1,1}+24\right)$	
		11/23	2	3	43/31	40/40	(mod48)	
	2	1/13	2	2	45/9	44/20	$\left(\rho_{1,1}+24\right)$	$k_{1}$
		5/17	2	2	1/13	4/28	(mod48)	(mod48)
		7/19	0	2	31/43	24/0	$\rho_{1,1}$	
		11/23	0	2	35/47	0/24	(mod48)	
	3	1/13	0	3	13/1	12/12	$\rho_{1,1}$	$\left(k_{1}+24\right)$
		5/17	0	3	41/29	12/12	(mod48)	(mod48)
		7/19	2	1	15/3	8/8	$\left(\rho_{1,1}+24\right)$	
		11/23	2	1	43/31	40/40	(mod48)	
	4	1/13	2	4	45/9	44/20	$\left(\rho_{1,1}+24\right)$	$k_{1}$
		5/17	2	4	1/13	4/28	(mod48)	(mod48)
		7/19	0	4	7/19	0/24	$\rho_{1,1}$	
		11/23	0	4	11/23	24/0	(mod48)	
1	1	3/15	3	1	35/23	8/8	$\rho_{1,1}$	$k_{1}$
		5/17	1	3	17/5	36/36	$\left(\rho_{1,1}+24\right)$	(mod48)
		9/21	1	3	45/33	20/20	(mod48)	
		11/23	3	1	43/31	40/40	$\rho_{1,1}$	
	2	3/15	1	2	3/15	8/32	$\rho_{1,1}$	$k_{1}$
		5/17	3	2	25/37	28/4	$\left(\rho_{1,1}+24\right)$	(mod48)
		9/21	3	2	29/41	20/44	(mod48)	
		11/23	1	2	11/23	24/0	$\rho_{1,1}$	
	3	3/15	3	3	35/23	8/8	$\rho_{1,1}$	$k_{1}$
		5/17	1	1	17/5	36/36	$\left(\rho_{1,1}+24\right)$	(mod48)
		9/21	1	1	45/33	20/20	(mod48)	
		11/23	3	3	43/31	40/40	$\rho_{1,1}$	
	4	3/15	1	4	3/15	8/32	$\rho_{1,1}$	$k_{1}$
		5/17	3	4	1/13	4/28	$\left(\rho_{1,1}+24\right)$	(mod48)
		9/21	3	4	5/17	44/20	(mod48)	
		11/23	1	4	11/23	24/0	$\rho_{1,1}$	

**Table 6:** Coefficients of an inverse 4-PP for a 4-PP (mod48Ψ) (Part II). For $f_{1}\;\left(\mathrm{mod}\;48\right)=f_{1,24}+24$, $\rho_{1}\;\left(\mathrm{mod}\;48\right)=\left(\rho_{1,f_{1,24}}+24\right)\;\left(\mathrm{mod}\;48\right)$.

$k_{3,f}$	$k_{2,f}$	$f_{1,24}$	$k_{3,\rho }$	$k_{2,\rho }$	$\rho_{1,f_{1,24}}$ for	*k* for	$\rho_{1,f_{1,24}}$ for	*k* for
					$k_{\Psi ,12}=1$	$k_{\Psi ,12}=1$	$k_{\Psi ,12}=7$	$k_{\Psi ,12}=7$
					($\rho_{1,1}$)	($k_{1}$)		
2	1	1/13	2	1	37/25	36/36	$\rho_{1,1}$	$\left(k_{1}+24\right)$
		3/15	0	3	19/7	8/8	$\left(\rho_{1,1}+24\right)$	(mod48)
		7/19	0	3	47/35	40/40	(mod48)	
		9/21	2	1	45/33	20/20	$\rho_{1,1}$	
	2	1/13	0	2	5/17	4/28	$\rho_{1,1}+24$	$k_{1}$
		3/15	2	2	27/39	32/8	$\rho_{1,1}$	(mod48)
		7/19	2	2	31/43	24/0	(mod48)	
		9/21	0	2	13/25	20/44	$\rho_{1,1}+24$	
	3	1/13	2	3	37/25	36/36	$\rho_{1,1}$	$\left(k_{1}+24\right)$
		3/15	0	1	19/7	8/8	$\left(\rho_{1,1}+24\right)$	(mod48)
		7/19	0	1	47/35	40/40	(mod48)	
		9/21	2	3	45/33	20/20	$\rho_{1,1}$	
	4	1/13	0	4	5/17	4/28	$\rho_{1,1}+24$	$k_{1}$
		3/15	2	4	3/15	8/32	$\rho_{1,1}$	(mod48)
		7/19	2	4	7/19	0/24	(mod48)	
		9/21	0	4	13/25	20/44	$\rho_{1,1}+24$	
3	1	1/13	3	3	13/1	12/12	$\left(\rho_{1,1}+24\right)$	$k_{1}$
		5/17	3	3	41/29	12/12	(mod48)	(mod48)
		7/19	1	1	47/35	40/40	$\rho_{1,1}$	
		11/23	1	1	27/15	8/8	(mod48)	
	2	1/13	1	2	29/41	28/4	$\left(\rho_{1,1}+24\right)$	$k_{1}$
		5/17	1	2	33/45	20/44	(mod48)	(mod48)
		7/19	3	2	7/19	0/24	$\rho_{1,1}$	
		11/23	3	2	11/23	24/0	(mod48)	
	3	1/13	3	1	13/1	12/12	$\left(\rho_{1,1}+24\right)$	$k_{1}$
		5/17	3	1	41/29	12/12	(mod48)	(mod48)
		7/19	1	3	47/35	40/40	$\rho_{1,1}$	
		11/23	1	3	27/15	8/8	(mod48)	
	4	1/13	1	4	5/17	4/28	$\left(\rho_{1,1}+24\right)$	$k_{1}$
		5/17	1	4	9/21	44/20	(mod48)	(mod48)
		7/19	3	4	7/19	0/24	$\rho_{1,1}$	
		11/23	3	4	11/23	24/0	(mod48)	

**Table 7:** Coefficients of an inverse 4-PP for a 4-PP (mod48Ψ) (Part III). For $f_{1}\;\left(\mathrm{mod}\;48\right)=f_{1,24}+24$, $\rho_{1}\;\left(\mathrm{mod}\;48\right)=\left(\rho_{1,f_{1,24}}+24\right)\;\left(\mathrm{mod}\;48\right)$.

$k_{3,f}$	$k_{2,f}$	$f_{1,24}$	$k_{3,\rho }$	$k_{2,\rho }$	$\rho_{1,f_{1,24}}$ for	*k* for	$\rho_{1,f_{1,24}}$ for	*k* for
					$k_{\Psi ,12}=5$	$k_{\Psi ,12}=5$	$k_{\Psi ,12}=11$	$k_{\Psi ,12}=11$
					($\rho_{1,5}$)	($k_{5}$)		
0	1	1/13	0	1	13/1	12/12	$\rho_{1,5}$	$\left(k_{5}+24\right)$
		5/17	0	1	41/29	12/12	(mod48)	(mod48)
		7/19	2	3	47/35	8/8	$\left(\rho_{1,5}+24\right)$	
		11/23	2	3	27/15	40/40	(mod48)	
	2	1/13	2	2	29/41	44/20	$\left(\rho_{1,5}+24\right)$	$k_{5}$
		5/17	2	2	33/45	4/28	(mod48)	(mod48)
		7/19	0	2	31/43	24/0	$\rho_{1,5}$	
		11/23	0	2	35/47	0/24	(mod48)	
	3	1/13	0	3	13/1	12/12	$\rho_{1,5}$	$\left(k_{5}+24\right)$
		5/17	0	3	41/29	12/12	(mod48)	(mod48)
		7/19	2	1	47/35	8/8	$\left(\rho_{1,5}+24\right)$	
		11/23	2	1	27/15	40/40	(mod48)	
	4	1/13	2	4	29/41	44/20	$\left(\rho_{1,5}+24\right)$	$k_{5}$
		5/17	2	4	33/ 45	4/28	(mod48)	(mod48)
		7/19	0	4	7/19	0/24	$\rho_{1,5}$	
		11/23	0	4	11/23	24/0	(mod48)	
1	1	1/13	1	3	37/25	36/36	$\rho_{1,5}+24$	$k_{5}$
		3/15	3	1	19/7	40/40	$\rho_{1,5}$	(mod48)
		7/19	3	1	47/35	8/8	(mod48)	
		9/21	1	3	45/33	4/4	$\rho_{1,5}+24$	
	2	1/13	3	2	5/17	20/44	$\rho_{1,5}+24$	$k_{5}$
		3/15	1	2	3/15	40/16	$\rho_{1,5}$	(mod48)
		7/19	1	2	7/19	0/24	(mod48)	
		9/21	3	2	13/25	4/28	$\rho_{1,5}+24$	
	3	1/13	1	1	37/25	36/36	$\rho_{1,5}+24$	$k_{5}$
		3/15	3	3	19/7	40/40	$\rho_{1,5}$	(mod48)
		7/19	3	3	47/35	8/8	(mod48)	
		9/21	1	1	45/33	4/4	$\rho_{1,5}+24$	
	4	1/13	3	4	29/41	44/20	$\rho_{1,5}+24$	$k_{5}$
		3/15	1	4	3/15	40/16	$\rho_{1,5}$	(mod48)
		7/19	1	4	7/19	0/24	(mod48)	
		9/21	3	4	37/1	28/4	$\rho_{1,5}+24$	

**Table 8:** Coefficients of an inverse 4-PP for a 4-PP (mod48Ψ) (Part IV). For $f_{1}\;\left(\mathrm{mod}\;48\right)=f_{1,24}+24$, $\rho_{1}\;\left(\mathrm{mod}\;48\right)=\left(\rho_{1,f_{1,24}}+24\right)\;\left(\mathrm{mod}\;48\right)$.

$k_{3,f}$	$k_{2,f}$	$f_{1,24}$	$k_{3,\rho }$	$k_{2,\rho }$	$\rho_{1,f_{1,24}}$ for	*k* for	$\rho_{1,f_{1,24}}$ for	*k* for
					$k_{\Psi ,12}=5$	$k_{\Psi ,12}=5$	$k_{\Psi ,12}=11$	$k_{\Psi ,12}=11$
					($\rho_{1,5}$)	($k_{5}$)		
2	1	3/15	0	3	35/23	40/40	$\rho_{1,5}+24$	$\left(k_{5}+24\right)$
		5/17	2	1	17/5	36/36	$\rho_{1,5}$	(mod48)
		9/21	2	1	45/33	4/4	(mod48)	
		11/23	0	3	43/31	8/8	$\rho_{1,5}+24$	
	2	3/15	2	2	27/39	16/40	$\rho_{1,5}$	$k_{5}$
		5/17	0	2	25/37	44/20	$\left(\rho_{1,5}+24\right)$	(mod48)
		9/21	0	2	29/41	4/28	(mod48)	
		11/23	2	2	35/47	0/24	$\rho_{1,5}$	
	3	3/15	0	1	35/23	40/40	$\rho_{1,5}+24$	$\left(k_{5}+24\right)$
		5/17	2	3	17/5	36/36	$\rho_{1,5}$	(mod48)
		9/21	2	3	45/33	4/4	(mod48)	
		11/23	0	1	43/31	8/8	$\rho_{1,5}+24$	
	4	3/15	2	4	3/15	40/16	$\rho_{1,5}$	$k_{5}$
		5/17	0	4	25/37	44/20	$\left(\rho_{1,5}+24\right)$	(mod48)
		9/21	0	4	29/41	4/28	(mod48)	
		11/23	2	4	11/23	24/0	$\rho_{1,5}$	
3	1	1/13	3	3	13/1	12/12	$\left(\rho_{1,5}+24\right)$	$k_{5}$
		5/17	3	3	41/29	12/12	(mod48)	(mod48)
		7/19	1	1	15/3	40/40	$\rho_{1,5}$	
		11/23	1	1	43/31	8/8	(mod48)	
	2	1/13	1	2	45/9	28/4	$\left(\rho_{1,5}+24\right)$	$k_{5}$
		5/17	1	2	1/13	20/44	(mod48)	(mod48)
		7/19	3	2	7/19	0/24	$\rho_{1,5}$	
		11/23	3	2	11/23	24/0	(mod48)	
	3	1/13	3	1	13/1	12/12	$\left(\rho_{1,5}+24\right)$	$k_{5}$
		5/17	3	1	41/29	12/12	(mod48)	(mod48)
		7/19	1	3	15/3	40/40	$\rho_{1,5}$	
		11/23	1	3	43/31	8/8	(mod48)	
	4	1/13	1	4	21/33	4/28	$\left(\rho_{1,5}+24\right)$	$k_{5}$
		5/17	1	4	25/37	44/20	(mod48)	(mod48)
		7/19	3	4	7/19	0/24	$\rho_{1,5}$	
		11/23	3	4	11/23	24/0	(mod48)	

**Table 9:** Solutions of Equations (68), (70), (72), (89), (91), and (93) for $k_{3,f}\in \left\{0,2\right\}$.

Equation	$k_{\Psi }$	$k_{3,f}$	$k_{2,f}^{\prime }$	$f_{1}\;\left(\mathrm{mod}\;8\right)$
(68)	1	0	1 or 5	3
		0	3 or 7	7
		2	1 or 3 or 5 or 7	3
	3	0	1 or 3 or 5 or 7	7
		2	1 or 5	7
		2	3 or 7	3
(70)	1	0	1 or 5	5
		0	3 or 7	1
		2	1 or 3 or 5 or 7	5
	3	0	1 or 3 or 5 or 7	1
		2	1 or 5	1
		2	3 or 7	5
(89)	1	0	1 or 3 or 5 or 7	1
		2	1 or 5	1
		2	3 or 7	5
	3	0	1 or 5	5
		0	3 or 7	1
		2	1 or 3 or 5 or 7	5
(91)	1	0	1 or 3 or 5 or 7	7
		2	1 or 5	7
		2	3 or 7	3
	3	0	1 or 5	3
		0	3 or 7	7
		2	1 or 3 or 5 or 7	3
(93)	1	0	1 or 3 or 5 or 7	3
		2	1 or 5	3
		2	3 or 7	7
	3	0	1 or 5	7
		0	3 or 7	3
		2	1 or 3 or 5 or 7	1

**Table 10:** Solutions of Equations (68), (70), (72), (89), (91), and (93) for $k_{3,f}\in \left\{1,3\right\}$.

Equation	$k_{\Psi }$	$k_{3,f}$	$k_{2,f}^{\prime }$	$f_{1}\;\left(\mathrm{mod}\;8\right)$
(68)	1 or 3	1	7	1 or 3 or 5 or 7
		3	3	3 or 7
		3	7	1 or 5
(70)	1 or 3	1	3	1 or 5
		1	7	3 or 7
		3	7	1 or 3 or 5 or 7
(89)	1 or 3	1	7	1 or 3 or 5 or 7
		3	3	1 or 5
		3	7	3 or 7
(91)	1 or 3	1	3	3 or 7
		1	7	1 or 5
		3	7	1 or 3 or 5 or 7
(93)	1 or 3	1	3	1 or 5
		1	7	3 or 7
		3	3	1 or 3 or 5 or 7

**Table 11:** Solutions of Equations (68), (70), (72), (89), (91), and (93) summarized from Table 9 and Table 10.

$k_{3,f}$	$k_{2,f}^{\prime }$	$f_{1}\;\left(\mathrm{mod}\;8\right)$
0 or 2	1 or 3 or 5 or 7	1 or 3 or 5 or 7
1 or 3	3 or 7	1 or 3 or 5 or 7

**Table 12:** Solutions of Equations (75), (77), and (79).

Equation	$k_{\Psi }\in$	$k_{3,f}$	$k_{2,f}$	$f_{1}\;\left(\mathrm{mod}\;48\right)\in$
(75)	{1,5,	0	1 or 3	{11,19} for $k_{\Psi }\in \left\{1,5\right\}$, {7,23} for $k_{\Psi }\in \left\{7,11\right\}$
	7,11}		2 or 4	{7,23}
		1	4	{3,5,9,11,15,17,21,23} for $k_{\Psi }\in \left\{1,7\right\}$,
				{1,3,7,9,13,15,19,21} for $k_{\Psi }\in \left\{5,11\right\}$
		2	1 or 3	{3,19} for $k_{\Psi }=1$, {3,11} for $k_{\Psi }=5$,
				{7,15} for $k_{\Psi }=7$, {15,23} for $k_{\Psi }=11$
			2 or 4	{3,19} for $k_{\Psi }\in \left\{1,7\right\}$, {3,11} for $k_{\Psi }\in \left\{5,11\right\}$
		3	2	{7,11,19,23}
			4	{1,5,13,17}
(77)	{1,5,	0	1 or 3	{5,13} for $k_{\Psi }\in \left\{1,5\right\}$, {1,17} for $k_{\Psi }\in \left\{7,11\right\}$
	7,11}		2 or 4	{1,17}
		1	2	{5,9,17,21} for $k_{\Psi }\in \left\{1,7\right\}$,
				{1,9,13,21} for $k_{\Psi }\in \left\{5,11\right\}$,
			4	{3,11,15,23} for $k_{\Psi }\in \left\{1,7\right\}$,
				{3,7,15,19} for $k_{\Psi }\in \left\{5,11\right\}$
		2	1 or 3	{13,21} for $k_{\Psi }=1$, {5,21} for $k_{\Psi }=5$,
				{1,9} for $k_{\Psi }=7$, {9,17} for $k_{\Psi }=11$
			2 or 4	{13,21} for $k_{\Psi }\in \left\{1,7\right\}$, {5,21} for $k_{\Psi }\in \left\{5,11\right\}$
		3	4	{1,5,7,11,13,17,19,23}
(79)	{1,5,	0	1 or 3	{1,17} for $k_{\Psi }\in \left\{1,5\right\}$, {5,13} for $k_{\Psi }\in \left\{7,11\right\}$
	7,11}		2 or 4	{5,13}
		1	2	{3,11,15,23} for $k_{\Psi }\in \left\{1,7\right\}$,
				{3,7,15,19} for $k_{\Psi }\in \left\{5,11\right\}$,
			4	{5,9,17,21} for $k_{\Psi }\in \left\{1,7\right\}$,
				{1,9,13,21} for $k_{\Psi }\in \left\{5,11\right\}$
		2	1 or 3	{1,9} for $k_{\Psi }=1$, {9,17} for $k_{\Psi }=5$,
				{13,21} for $k_{\Psi }=7$, {5,21} for $k_{\Psi }=11$
			2 or 4	{1,9} for $k_{\Psi }\in \left\{1,7\right\}$, {9,17} for $k_{\Psi }\in \left\{5,11\right\}$
		3	2	{1,5,7,11,13,17,19,23}

**Table 13:** Solutions of Equations (96), (98), and (100).

Equation	$k_{\Psi }\in$	$k_{3,f}$	$k_{2,f}$	$f_{1}\;\left(\mathrm{mod}\;48\right)\in$
(96)	{1,5,	0	1 or 3	{1,17} for $k_{\Psi }\in \left\{1,5\right\}$, {5,13} for $k_{\Psi }\in \left\{7,11\right\}$
	7,11}		2 or 4	{5,13}
		1	4	{3,5,9,11,15,17,21,23} for $k_{\Psi }\in \left\{1,7\right\}$,
				{1,3,7,9,13,15,19,21} for $k_{\Psi }\in \left\{5,11\right\}$
		2	1 or 3	{13,21} for $k_{\Psi }=1$, {5,21} for $k_{\Psi }=5$,
				{1,9} for $k_{\Psi }=7$, {9,17} for $k_{\Psi }=11$
			2 or 4	{13,21} for $k_{\Psi }\in \left\{1,7\right\}$, {5,21} for $k_{\Psi }\in \left\{5,11\right\}$
		3	2	{7,11,19,23}
			4	{1,5,13,17}
(98)	{1,5,	0	1 or 3	{7,23} for $k_{\Psi }\in \left\{1,5\right\}$, {11,19} for $k_{\Psi }\in \left\{7,11\right\}$
	7,11}		2 or 4	{7,23}
		1	2	{3,11,15,23} for $k_{\Psi }\in \left\{1,7\right\}$,
				{3,7,15,19} for $k_{\Psi }\in \left\{5,11\right\}$,
			4	{5,9,17,21} for $k_{\Psi }\in \left\{1,7\right\}$,
				{1,9,13,21} for $k_{\Psi }\in \left\{5,11\right\}$
		2	1 or 3	{7,15} for $k_{\Psi }=1$, {15,23} for $k_{\Psi }=5$,
				{3,19} for $k_{\Psi }=7$, {3,11} for $k_{\Psi }=11$
			2 or 4	{3,19} for $k_{\Psi }\in \left\{1,7\right\}$, {3,11} for $k_{\Psi }\in \left\{5,11\right\}$
		3	4	{1,5,7,11,13,17,19,23}
(100)	{1,5,	0	1 or 3	{11,19} for $k_{\Psi }\in \left\{1,5\right\}$, {7,23} for $k_{\Psi }\in \left\{7,11\right\}$
	7,11}		2 or 4	{11,19}
		1	2	{5,9,17,21} for $k_{\Psi }\in \left\{1,7\right\}$,
				{1,9,13,21} for $k_{\Psi }\in \left\{5,11\right\}$,
			4	{3,11,15,23} for $k_{\Psi }\in \left\{1,7\right\}$,
				{3,7,15,19} for $k_{\Psi }\in \left\{5,11\right\}$
		2	1 or 3	{3,19} for $k_{\Psi }=1$, {3,11} for $k_{\Psi }=5$,
				{7,15} for $k_{\Psi }=7$, {15,23} for $k_{\Psi }=11$
			2 or 4	{7,15} for $k_{\Psi }\in \left\{1,7\right\}$, {15,23} for $k_{\Psi }\in \left\{5,11\right\}$
		3	2	{1,5,7,11,13,17,19,23}

**Table 14:** Solutions of Equations (75), (77), (79), (96), (98), and (100) summarized from Table 12 and Table 13.

$k_{3,f}$	$k_{2,f}$	$f_{1}\;\left(\mathrm{mod}\;48\right)\in$
0	1 or 2 or 3 or 4	{1,5,7,11,13,17,19,23}
2	1 or 2 or 3 or 4	{1,3,7,9,13,15,19,21} for $k_{\Psi }\in \left\{1,7\right\}$, {3,5,9,11,15,17,21,23} for $k_{\Psi }\in \left\{5,11\right\}$
1	2 or 4	{3,5,9,11,15,17,21,23} for $k_{\Psi }\in \left\{1,7\right\}$, {1,3,7,9,13,15,19,21} for $k_{\Psi }\in \left\{5,11\right\}$
3	2 or 4	{1,5,7,11,13,17,19,23}

**Table 15:** Minimum distances ($d_{min}$) and corresponding multiplicities ($N_{d_{min}}$), spread factors (*D*), nonlinearity degrees (ζ), and refined nonlinearity degrees ($\zeta^{\prime }$) for cubic permutation polynomials (CPPs) and for fourth degree permutation polynomials (4-PPs) with optimum minimum distance.

*L*	CPP	$d_{\min}$	$N_{d_{\min}}$	ζ	$\zeta^{\prime }$	*D*	4-PP	$d_{\min}$	$N_{d_{\min}}$	ζ	$\zeta^{\prime }$	*D*
592	$222x^{3}+148x^{2}+39x$	38	625	8	5	20	$37x^{4}+222x^{3}+$	36	1102	4	4	30
							$+37x^{2}+393x$					
656	$82x^{3}+164x^{2}+185x$	38	620	8	5	22	$41x^{4}+246x^{3}+$	36	1230	4	4	32
	(from [18])						$+41x^{2}+217x$					
688	$86x^{3}+0x^{2}+21x$	38	652	8	5	24	$43x^{4}+258x^{3}+$	36	1294	4	4	32
							$+43x^{2}+137x$					
752	$94x^{3}+188x^{2}+541x$	38	716	8	5	26	$47x^{4}+282x^{3}+$	36	1422	4	4	32
	(from [18])						$+47x^{2}+249x$					
816	$34x^{3}+0x^{2}+19x$	38	782	8	7	28	$17x^{4}+34x^{3}+$	36	1556	4	4	30
							$+85x^{2}+9x$					
848	$318x^{3}+212x^{2}+157x$	38	812	8	5	28	$53x^{4}+318x^{3}+$	36	1614	4	4	32
	(from [18])						$+53x^{2}+169x$					
912	$114x^{3}+114x^{2}+287x$	38	878	8	4	30	$19x^{4}+38x^{3}+$	36	1748	4	4	18
	(from [18])						$+95x^{2}+5x$					
944	$354x^{3}+0x^{2}+179x$	38	910	8	5	32	$59x^{4}+354x^{3}+$	36	1806	4	4	38
	(from [18])						$+59x^{2}+317x$					
976	$122x^{3}+0x^{2}+307x$	38	942	8	5	32	$61x^{4}+366x^{3}+$	36	1870	4	4	38
	(from [18])						$+61x^{2}+389x$					

## Appendix A

In [32], an efficient algorithm for the computing nonlinearity degree of a 4-PP was given. We remember that for interleaver lengths of the form (5), the coefficients of 4-PPs fulfilling conditions (6) when $3∤(p_{i}-1)$, are equivalent to the following ones: $f_{4}=\Psi$, $f_{3}=k_{3,f}\cdot 2\Psi$, with $k_{3,f}\in \left\{0,1,2,3\right\}$, $f_{2}=\left(2k_{L}k_{2,f}-1\right)\cdot \Psi$, with $k_{2,f}\in \left\{1,2,3,4\right\}$ and $k_{L}\in \left\{1,3\right\}$. Then, we have

(A1) $$\gcd(4f_{4},L)=4\Psi ,$$

(A2) $$\gcd\left(6,L\right)=2k_{L},$$

(A3) gcd(2,L)=2.

$$k_{0,f_{4}}={\left(\frac{4f_{4}}{\gcd(4f_{4},L}\right)}^{-1}\cdot \frac{L/\gcd\left(6,L\right)\cdot \tau_{3}}{\gcd(4f_{4},L)}\;\left(\mathrm{mod}\;\frac{L}{\gcd(4f_{4},L)}\right)=$$

(A4) $$={\left(\frac{4\Psi }{4\Psi }\right)}^{-1}\cdot \frac{8\Psi \cdot \tau_{3}}{4\Psi }\;\left(\mathrm{mod}\;\frac{16k_{L}\Psi }{4\Psi }\right)=2\tau_{3}\;\left(\mathrm{mod}\;4k_{L}\right)=2\tau_{3}.$$

For

(A5) $$k_{0}=k_{0,f_{4}}+L/\gcd\left(4f_{4},L\right)\cdot i=2\tau_{3}+4k_{L}\cdot i,$$

we have

$$3f_{3}k_{0}-6f_{4}k_{0}^{2}\;\;\left(\mathrm{mod}\;L\right)=3k_{3,f}\cdot 2\Psi \cdot \left(2\tau_{3}+4k_{L}\cdot i\right)-6\Psi \cdot {\left(2\tau_{3}+4k_{L}\cdot i\right)}^{2}\;\left(\mathrm{mod}\;16k_{L}\Psi \right)=$$

(A6) $$=12\Psi \cdot \left(\tau_{3}+2k_{L}\cdot i\right)\cdot \left(k_{3,f}-2\tau_{3}-4k_{L}\cdot i\right)\;\left(\mathrm{mod}\;16k_{L}\Psi \right),$$

(A7) $$L/\gcd\left(2,L\right)\cdot \tau_{2}\;\;\left(\mathrm{mod}\;L\right)=8k_{L}\Psi \cdot \tau_{2}\;\left(\mathrm{mod}\;16k_{L}\Psi \right),$$

$$2f_{2}k_{0}-3f_{3}k_{0}^{2}+4f_{4}k_{0}^{3}\;\;\left(\mathrm{mod}\;L\right)=2\cdot \left(2k_{L}k_{2,f}-1\right)\cdot \Psi \cdot \left(2\tau_{3}+4k_{L}\cdot i\right)-3k_{3,f}\cdot 2\Psi \cdot {\left(2\tau_{3}+4k_{L}\cdot i\right)}^{2}+$$

$$+4\Psi \cdot {\left(2\tau_{3}+4k_{L}\cdot i\right)}^{3}\;\left(\mathrm{mod}\;16k_{L}\Psi \right)=4\Psi \cdot \left(\tau_{3}+2k_{L}\cdot i\right)\cdot$$

(A8) $$\cdot \left(2k_{L}k_{2,f}-1-3k_{3,f}\cdot \left(2\tau_{3}+4k_{L}\cdot i\right)+{\left(2\tau_{3}+4k_{L}\cdot i\right)}^{2}\right)\;\left(\mathrm{mod}\;16k_{L}\Psi \right)$$

$$(-L/\gcd\left(6,L\right)\cdot \tau_{3}-L/\gcd\left(2,L\right)\cdot \tau_{2})\;\;\left(\mathrm{mod}\;L\right)=$$

(A9) $$=-8\Psi \cdot \left(\tau_{3}+k_{L}\cdot \tau_{2}\right)\;\left(\mathrm{mod}\;16k_{L}\Psi \right).$$

Then condition $3f_{3}k_{0}-6f_{4}k_{0}^{2}\;\;\left(\mathrm{mod}\;L\right)=L/\gcd\left(2,L\right)\cdot \tau_{2}\;\;\left(\mathrm{mod}\;L\right)$ is equivalent to

$$12\Psi \cdot \left(\tau_{3}+2k_{L}\cdot i\right)\cdot \left(k_{3,f}-2\tau_{3}-4k_{L}\cdot i\right)\;\left(\mathrm{mod}\;16k_{L}\Psi \right)=8k_{L}\Psi \cdot \tau_{2}\;\left(\mathrm{mod}\;16k_{L}\Psi \right)$$

or

$$3\cdot \left(\tau_{3}+2k_{L}\cdot i\right)\cdot \left(k_{3,f}-2\tau_{3}-4k_{L}\cdot i\right)\;\left(\mathrm{mod}\;4k_{L}\right)=2k_{L}\cdot \tau_{2}\;\left(\mathrm{mod}\;4k_{L}\right)$$

or

(A10) $$3\cdot \left(\tau_{3}+2k_{L}\cdot i\right)\cdot \left(k_{3,f}-2\tau_{3}\right)\;\left(\mathrm{mod}\;4k_{L}\right)=2k_{L}\cdot \tau_{2}\;\left(\mathrm{mod}\;4k_{L}\right);$$

and condition $2f_{2}k_{0}-3f_{3}k_{0}^{2}+4f_{4}k_{0}^{3}\;\;\left(\mathrm{mod}\;L\right)=\left(-L/\gcd\left(6,L\right)\cdot \tau_{3}-L/\gcd\left(2,L\right)\cdot \tau_{2}\right)\;\;\left(\mathrm{mod}\;L\right)$ is equivalent to

$$4\Psi \cdot \left(\tau_{3}+2k_{L}\cdot i\right)\cdot \left(2k_{L}k_{2,f}-1-3k_{3,f}\cdot \left(2\tau_{3}+4k_{L}\cdot i\right)+{\left(2\tau_{3}+4k_{L}\cdot i\right)}^{2}\right)\;\left(\mathrm{mod}\;16k_{L}\Psi \right)=$$

$$=-8\Psi \cdot \left(\tau_{3}+k_{L}\cdot \tau_{2}\right)\;\left(\mathrm{mod}\;16k_{L}\Psi \right)$$

or

$$\left(\tau_{3}+2k_{L}\cdot i\right)\cdot \left(2k_{L}k_{2,f}-1-3k_{3,f}\cdot \left(2\tau_{3}+4k_{L}\cdot i\right)+{\left(2\tau_{3}+4k_{L}\cdot i\right)}^{2}\right)\;\left(\mathrm{mod}\;4k_{L}\right)=$$

$$=-2\cdot \left(\tau_{3}+k_{L}\cdot \tau_{2}\right)\;\left(\mathrm{mod}\;4k_{L}\right)$$

or

(A11) $$\left(\tau_{3}+2k_{L}\cdot i\right)\cdot \left(2k_{L}k_{2,f}-1-6k_{3,f}\cdot \tau_{3}+8\tau_{3}^{2}\right)\;\left(\mathrm{mod}\;4k_{L}\right)=-2\cdot \left(\tau_{3}+k_{L}\cdot \tau_{2}\right)\;\left(\mathrm{mod}\;4k_{L}\right).$$

Because $L/\gcd\left(6,L\right)\cdot \tau_{3}=8\Psi \cdot \tau_{3}$, we have $\gcd\left(4f_{4},L\right)|L/\gcd\left(6,L\right)\cdot \tau_{3}$, $\forall \tau_{3}\in \left\{0,1,\ldots ,2k_{L}-1\right\}$. Then, with Equations (A1)–(A11), Algorithm 2 from [32] becomes Algorithm A1 in this paper. Run Algorithm A1 for every $k_{3,f}\in \left\{0,1,2,3\right\}$, $k_{2,f}\in \left\{1,2,3,4\right\}$, and $k_{L}\in \left\{1,3\right\}$, (120) result.

**Algorithm 1:** Algorithm for computing the nonlinearity degree ζ for a 4-PP for interleaver lengths of the form (5) and the coefficients of 4-PP fulfilling conditions (6) when $3∤(p_{i}-1)$.
**input**: Values $k_{L}$ for the interleaver length, and $k_{3,f}$, $k_{2,f}$ for the 4-PP. **output**: Nonlinearity degree ζ for the 4-PP. $\zeta \leftarrow 16k_{L}/N_{k_{0}}$;
